# Predicting regulatory variants using a dense epigenomic mapped CNN model elucidated the molecular basis of trait-tissue associations

**DOI:** 10.1093/nar/gkaa1137

**Published:** 2020-12-09

**Authors:** Guangsheng Pei, Ruifeng Hu, Yulin Dai, Astrid Marilyn Manuel, Zhongming Zhao, Peilin Jia

**Affiliations:** Center for Precision Health, School of Biomedical Informatics, The University of Texas Health Science Center at Houston, Houston, TX 77030, USA; Center for Precision Health, School of Biomedical Informatics, The University of Texas Health Science Center at Houston, Houston, TX 77030, USA; Center for Precision Health, School of Biomedical Informatics, The University of Texas Health Science Center at Houston, Houston, TX 77030, USA; Center for Precision Health, School of Biomedical Informatics, The University of Texas Health Science Center at Houston, Houston, TX 77030, USA; Center for Precision Health, School of Biomedical Informatics, The University of Texas Health Science Center at Houston, Houston, TX 77030, USA; Human Genetics Center, School of Public Health, The University of Texas Health Science Center at Houston, Houston, TX 77030, USA; MD Anderson Cancer Center UTHealth Graduate School of Biomedical Sciences, Houston, TX 77030, USA; Department of Biomedical Informatics, Vanderbilt University Medical Center, Nashville, TN 37203, USA; Center for Precision Health, School of Biomedical Informatics, The University of Texas Health Science Center at Houston, Houston, TX 77030, USA

## Abstract

Assessing the causal tissues of human complex diseases is important for the prioritization of trait-associated genetic variants. Yet, the biological underpinnings of trait-associated variants are extremely difficult to infer due to statistical noise in genome-wide association studies (GWAS), and because >90% of genetic variants from GWAS are located in non-coding regions. Here, we collected the largest human epigenomic map from ENCODE and Roadmap consortia and implemented a deep-learning-based convolutional neural network (CNN) model to predict the regulatory roles of genetic variants across a comprehensive list of epigenomic modifications. Our model, called DeepFun, was built on DNA accessibility maps, histone modification marks, and transcription factors. DeepFun can systematically assess the impact of non-coding variants in the most functional elements with tissue or cell-type specificity, even for rare variants or de novo mutations. By applying this model, we prioritized trait-associated loci for 51 publicly-available GWAS studies. We demonstrated that CNN-based analyses on dense and high-resolution epigenomic annotations can refine important GWAS associations in order to identify regulatory loci from background signals, which yield novel insights for better understanding the molecular basis of human complex disease. We anticipate our approaches will become routine in GWAS downstream analysis and non-coding variant evaluation.

## INTRODUCTION

In the past 15 years, genome-wide association studies (GWAS) have identified thousands of susceptibility variants associated with human complex diseases and traits (1,2). It remains an open challenge to identify true functional variants (e.g. causal variants) from background signals (3). In addition, >90% of the genetic variants identified from GWAS lie outside of protein-coding regions (4) and some are in gene deserts (5), implicating that they influence disease risk through transcriptional regulation mechanisms (6). However, the distinct transcription regulatory functions across different tissues and cell types have aggravated the challenge of variant prioritization and interpretation of variant effects on regulatory elements (7). Increasing evidence shows that disease-associated variants are likely to perturb genes and regulatory modules within specific disease-relevant tissues or cell types (8). Previous studies have revealed that risk variants of psychiatric diseases tend to be in neuron-specific regulatory regions (9–11). For example, schizophrenia (SCZ) risk variants rs12293670 and rs2514218 were attributed to two genes specifically expressed in brain: *NRGN* and *DRD2* (12); ADHD index variants rs28452470 and rs2243638 were related to two brain-specifically-expressed genes: *CADPS2* and *RNF219-AS1* (13). Furthermore, variants of immune-related traits, such as inflammatory bowel disease (IBD) risk variant rs653178 has trans-eQTL effects on two genes specifically expressed in immune cells: *TAGAP* and *STAT1* (14). To elucidate the potential molecular functions of non-coding variants and to derive biological insights from a vast array of GWAS signals, there is a pressing need to prioritize variants in a tissue or cell-type specific manner (15).

To help annotate non-coding regions of the genome, large-scale experimental mapping of epigenomic modifications have been conducted by several large consortia, including the ENCyclopedia of DNA Elements (ENCODE) (16) and Roadmap Epigenomics Project (17). These epigenomic maps provide valuable resources for prioritizing disease-associated variants by considering their overlap with regulatory elements enriched in disease-associated signals (18,19). Moreover, convolutional neural network (CNN) models for studying epigenomic features are arising as a dominant approach to investigate regulatory motifs within the genomic context. A multilayer CNN network is well suited to capture high-level information (20). Currently, there are lots of CNN-based frameworks for prioritizing non-coding genomic variants, such as DeepBind (20), DeepSEA (21), Basset (22), DanQ (23), Basenji (24), DeFine (7), ExPecto (25) and Seqweaver (26). CNN models offer computational predictions of the likely regulatory effects of genomic variation based on disruption or creation of regulatory motifs discovered by the convolutional filters. These computational predictions thereby facilitate the downstream prediction of chromatin accessibility and regulatory modifications (21,22).

While CNN models provide an attractive framework for variant prioritization, there are currently still several limitations to improve upon. Firstly, their models are still based on incomplete epigenomic annotations, where many marks miss the annotation of greatly important disease-relevant tissues (21–23). Secondly, they underestimated the complexity of chromatin features in epigenome. It is reported that 80% of the genome is comprised of biochemically functional regions (16), while most previous models only take 30% genomic regions as epigenomic active sites, due to limited chromatin features collection (21–23). Thirdly, the quality of reference epigenomes is highly variable. Reference epigenomes of previous models typically consider only a small number of replicates, making them lack statistical power and sensitive to experimental noise.

To address these fundamental issues, we overcome these limitations and present a dense epigenomic map of the human epigenome by incorporating 7879 datasets across 225 tissues/cell lines and 322 marks (including transcription factors (TFs)) from ENCODE (16) and Roadmap (17) consortia. These chromatin feature annotations greatly surpass previous epigenomic maps in scope, scale, and coverage of biological space. Moreover, we trained a CNN-based deep learning model, DeepFun, on a broad collection of genome-wide epigenomic profiles to capture chromatin regulatory features. We use different genetic variant datasets to assess the performance at single-base resolution, demonstrating that DeepFun can systematically study the impact of a variant with tissue or cell-type specificity. Lastly, we apply the DeepFun model to systematically prioritize trait-associated regulatory loci from 51 publicly-available GWAS studies. Then we recognized and compared their potential associated tissues (27). Our results demonstrated that refined GWAS regulatory loci can provide a lot of novel insights into trait-tissue relationships. We anticipate the CNN model on dense epigenomic maps will be a valuable approach for both gene-regulatory studies and disease studies seeking to elucidate the molecular basis of complex disorders.

## MATERIALS AND METHODS

### Primary chromatin feature collection and processing

The DeepFun framework expanded 7879 chromatin features from ENCODE Project Consortium and Roadmap Epigenomics Consortium (6 May 2019), including 1548 DNase-seq datasets and 6331 epigenomic ChIP-seq datasets. These ChIP-seq datasets included 1536 histone modification marks and 4795 TFs binding profiles. According to their functional category and completeness, we classified these assays into two tiers. Tier 1 assays (18 marks, 3451 total experiments): DNase-seq, H3K14ac, H3K18ac, H3K23ac, H3K23me2, H3K27ac, H3K27me3, H3K36me3, H3K4ac, H3K4me1, H3K4me2, H3K4me3, H3K79me1, H3K79me2, H3K9ac, H3K9me2, H3K9me3 and CTCF; Tier 2 assays (305 marks, 4428 total experiments): POLR2A, H2AFZ, EP300, RAD21 and all others transcription factors. We removed poor quality chromatin profiles and kept only profiles with appropriately matched ChIP-seq controls. For all datasets, the uniformly processed them with the same computational pipelines using HotSpot algorithm with 1% false-discovery rate (22). We used the coordinates of the optimal peaks sets produced with the Irreproducible Discovery Rate (IDR) procedure for further analysis. Any peaks within 1000 bp to rRNA, snRNA, snoRNA and tRNA genes were removed to avoid confusion based on GENCODE annotation (26). Each dataset was assigned a unique accession ID (Supplementary Table S1).

### DeepFun input feature encoding

The training, validation and test datasets for the convolutional neural network training were created analogously to the approach used in Basset (22). Briefly, we created 1000 bp genomic intervals to all narrow peaks by extending 500 bp on each side of the midpoint of the peak. We then greedily merged peaks based on their distance to an adjacent peak, until no peaks overlapped by >200 bp. The center of the merged peak was determined as a weighted average of the midpoints of the merged peaks from individual profiles, these peaks were regarded as potential epigenomic active sites. Finally, we use BEDTools software (v2.26.0) (28) mapping coordinates of epigenomic active sites against the human reference genome build version hg19.

We classified these assays into two separately model according to their functional category and completeness, these resulted in 2 298 761 epigenomic active sites with assigned presence (positive dataset) or absence (negative dataset) across 3451 features in model A, and 2 400 512 epigenomic active sites with 4428 features in model B. Subsequently, we encoded these epigenomic active sites as one-hot code position weight matrix (PWM), and mapping these sequences into a four-row binary matrix, corresponding A, C, G and T at each position. Moreover, for each 1000 bp sequence, we created an accompanying binary vector denoting which of these chromatin features showed a signal peak overlapping with sequence. To investigate histone marks and TFs specificity on human epigenome, we conducted Uniform Manifold Approximation and Projection (UMAP) dimension reduction analysis (29) on both epigenomic active sites binary vector matrix.

### CNN model training

Deep convolutional network is a type of multilayer neural network that is specifically parameterized to take advantage of known spatial structure (30). We applied an extended version of the Basset model (22) with default three-layer architecture to learn parameters, implemented in the Torch7 framework (http://torch.ch). We trained the CNN model with different hyperparameter settings. To reach the appropriate performance of the model, we applied stochastic gradient descent to learn all model parameters, including those representing the number of convolutional filters, dimension of convolutional filters, dimension of pool size and learning rate, by using RMSprop updates on minibatches (31). Simply, the network computes predictions for small batches of sequences during training. After we compare the difference between predicted and real experiment measurement (loss function), then, model parameters will be updated through back propagation algorithm. Model with the smallest loss value in the validation set was saved as best model.

After hyperparameter optimization, we applied 300 convolutional filters (width 19) scans across all sequences PWM. After convolving the PWM of the sequence, the rectified linear (ReLU) nonlinearity active function was applied connected by a maximum ‘pool’ layer (pool width 3, pad width 18) were incorporated to DeepFun model. The second and third convolutional layers were operated on the output of the prior layer. Therefore, they were capable of capturing more complex patterns in larger spatial ranges. After three convolutional layers, two fully connected artificial neural network hidden layers with 30% dropout rate were applied to avoid over fitting. Finally, a fully connected sigmoid transformation layer is applied to represent the predicted accessibility probability. Since DNase-seq and ChIP-seq are not strand specific-assays, the reverse complement sequence gives the same epigenetic signal as the original sequence. We augmented the dataset by including the reverse complement of each example doubles the number of sequence-signal pairs (32).

From total epigenomic active sites in both models, we randomly selected 80% for training and another 10% for validation, leaving 10% remain epigenomic active sites for testing. Training and testing sets were split strictly without any overlapping. We trained these sequence features across all chromatin profile predictors with a multitask model. We used the area under receiver operating characteristic (AUC) to evaluate the performance on validation and testing sets. The predicted accessible probability for each profile was computed separately. The network training was stopped until the loss in the validation set did not decrease within 12 successive epochs of Bayesian optimization. Each epoch of training takes about 8 h under NVIDIA Tesla V100 32GB GPU computing accelerator with Intel (R) Xeon (R) Platinum 8180 CPU.

### Capture first layer convolutional filters to functional motifs

CNN model could recognize specific sequence motifs to project this recognition through after iterating over many batches of training data. To assess the impact and contribution of each filter, we investigated each filter information content (IC) base on previous studies (22,25). Simply, we convert initial convolutional layer learned filters into probabilistic PWMs. Then, we apply TomTom (v4.12.0) software (33) mapping learned filters to potential human TF binding motifs download from CIS-BP database (34). The information content for a motif was defined as IC = $ - \mathop \sum \limits_{i,j} p\,{\rm{lo}}{{\rm{g}}_2}\ ({p_j}) + \mathop \sum \limits_{i,j} {m_j}{\rm{lo}}{{\rm{g}}_2}\ ({m_{ij}})$, where *m* is the 19 × 4 matrix of nucleotide probabilities for the motif, and *p* is the length four array of background nucleotide probabilities. We use FDR value 0.1 as threshold. When a given filter showed high similarity to multiple motifs, only the best match motif was selected for downstream analysis. We visualized motifs using *ggseqlogo* package (35).

To quantify each filter's influence in the initial convolution layer, we nullified each filter from the model by setting all output from the filter to its mean output over all nucleotides in the test set. Thereby, all information from an initial filter was obstructed when passing forward through the network. The new predicted accessibility in each profile was compared to the originally predicted accessibility to represent the influence of the filter. As disagreement influence of the same filter across different profiles, we calculated each filter's global influence as this vector's sum of squares (22). These procedures were repeated 10 times and the average values were calculated for downstream analysis.

To uncover the correlation of each filter's influence across different measurements, we collected matched RNA-seq expression profiles from ENCODE and consortia (16), resulting in a total of 138 RNA-seq samples across 44 tissues. For 108 filters in model A were captured by CIS-BP database TF binding motifs, we calculate the *Pearson correlation coefficient* (PCC) between each convolution filter's influence score and corresponding filter captured gene expression level across different measurements. We used two thresholds grouping filters information content for a motif: high confident captured filters, FDR_TomTom_ < 10^−4^, and low confident captured filters, 10^−4^ < FDR_TomTom_ < 0.1.

### Application of CNN model to prioritize regulatory variants

The DeepFun model is designed to predict the functional impacts of sequence alterations at single-nucleotide resolution. For each variant, DeepFun will consider variant nearby 1000 bp region context information, and then predict the ‘activity’ probability of sequences contain reference allele or alternative allele. Here, the ‘activity’ means the accessibility or binding affinity for DNase-seq or histone modifications and TFs, respectively. To evaluate the impact of variant, we implemented previous methods defined by SNP Activity Difference (SAD): *Alt* – *Ref*, where *Ref* and *Alt* represent the predicted activity probability for the reference allele/original sequence and the alternative allele/mutated sequence, respectively. Both bases predict activity probability range from 0 to 1. Variants have a higher positive SAD indicates that the alternative allele increases the epigenetic signal compared to the reference allele, while negative value indicates decrease the epigenetic signal. Although DeepFun models are trained jointly across a large dataset, each variant predicted functional score is independent as it is based on a single experiment assay.

### Functional validation of regulatory variants

We used two independent test datasets to evaluate the non-coding variants functional differentiating (pathogenic vs. benign variants, functional vs. nonfunctional), included 442 778 variants from the ClinVar database (36) downloaded on 15 May 2019, and 135 435 Autism *de novo* mutations from Simons Simplex Collection (SSC) cohort (37) downloaded on 18 November 2019. For ClinVar test variants, we grouped all non-coding variants into benign (true negatives, variants labeled as ‘benign’ or ‘likely benign’), pathogenic (true positives, variants labeled as ‘pathogenic’ or ‘likely pathogenic’) and uncertain significance (control). For SSC cohort test variants, we grouped all non-coding variants into unaffected and affected siblings. All test variants were submitted to DeepFun model to calculate their SAD score under individual profile. Variants were then stratified based on variant consequence (i.e. intergenic, 5′ and 3′ UTR, etc.). Based on research purpose difference, their average absolute SAD scores on target profiles were firstly calculated, followed by one side Wilcoxon rank-sum test.

### Canonical correspondence analysis

Canonical correspondence analysis (CCA) is a multivariate technique to illuminate the relationship between two sets of explanatory variables (38). The CCA results presented in this work is conducted by the R package *CCA* (39). Specifically, CCA projects the two variables onto a low-dimensional space where these variables are maximally correlated. In our case, we used CCA to investigate schizophrenia associated variants of functional impact scores versus tissue specificity.

Let *X* be a *N × P* matrix of SAD scores of *N* variants over *P* chromatin profiles. Similarly, let *Y* denote an *N × T* matrix recording the source of each profile over *T* tissues. Let ${{\boldsymbol a}^{\boldsymbol 1}} = {({\boldsymbol a}_{\boldsymbol 1}^{\boldsymbol 1}; \cdot \cdot \cdot ;{\boldsymbol a}_{\boldsymbol p}^{\boldsymbol 1})^{\boldsymbol T}}{\rm and}\,{{\boldsymbol b}^{\boldsymbol 1}} = {({\boldsymbol b}_{\boldsymbol 1}^{\boldsymbol 1}; \cdot \cdot \cdot ;{\boldsymbol b}_{\boldsymbol t}^{\boldsymbol 1})^{\boldsymbol T}}$ denote the two basis vectors. Then the projections of the two explanatory variables onto these basis vectors are given by:

$$\begin{eqnarray*} && {U^1} = X{a^1} = a_1^1{X^{[,1]}} + a_2^1{X^{[,2]}} + \cdot \cdot \cdot + a_p^1{X^p} \nonumber \\ && {\rm and}\,{V^1} = Y{b^1} = b_1^1{Y^1} + b_2^1{Y^2} + \cdot \cdot \cdot + b_t^1{Y^t}. \end{eqnarray*}$$

CCA seeks to find two vectors (*a* and *b*) to maximize the correlation $\rho = {\rm{cor}}( {{a^T}X,{\rm{\ }}{b^T}Y} )$. Thus, the correlations between two projections are mutually maximized as follows:

$$\begin{equation*}{\rho _1} = {\rm{cor}}\left( {{U^1},{\rm{\ }}{V^1}} \right) = \mathop {\max }\limits_{a,\ b} \left[ {cor\left( {Xa,\ Yb} \right)} \right]\end{equation*}$$

where the derived linear projections *U*^1^ and *V*^1^ are the first canonical components and ${\rho _1}$ refers to the canonical correlation between the first components. Note that the successively computed canonical correlations decrease by nature, i.e. ${\rho _1} \ge {\rho _2} \ge \ldots \ge {\rho _{{\rm{min}}\ ( {C,R} )}}$.

### Compiling trait-associated loci from GWAS data

We expanded 51 publicly-available GWAS studies based on our previous study (40). These GWAS studies span a wide range of phenotype measurements and can be categorized into several groups. For each GWAS trait, we filtered significant associated SNPs with chi-squared *P*-value < 10^−3^, defined as lead causal SNPs. Additionally, we applied DeepFun model to predict their potential regulatory effects. Since the mean and median SAD across all assays were very close to zero, we defined those SNPs with a maximum SAD score greater than 0.1 (or less than –0.1) as regulatory loci. To better examine these SNPs with genetic association, we employed Pascal software (41) mapped them to gene level, if these SNPs were located by the location within a range of 50 kb upstream or downstream of corresponding gene transcription start sites by taking into account of LD, gene length, and SNP density information. Any genes with at least one regulatory loci (max SAD > 0.1) were regarded as regulatory trait-associated genes (TAGs), while TAGs without regulatory loci were regarded as non-regulatory TAGs.

For both regulatory and non-regulatory TAGs, we compared their pLI scores, which downloaded from the Exome Aggregation Consortium (ExAC) project (42). Simply, the ExAC pLI score indicates the probability that a gene is intolerant to a loss of function (LoF) mutation. Genes with high pLI scores are LoF intolerant, whereby genes with low pLI scores are LoF tolerant. To assess whether regulatory or non-regulatory TAGs with higher chance overlap with LoF intolerant genes (ExAC pLI > 0.9) than expectation, we build a dichotomous 2 × 2 contingency table, followed by Fisher's exact test analysis.

Based on our previous study, we found most TAGs show strong tissue specific associations (15,27). However, there are still many traits that could not recapture biological associated tissue (15). To further investigate the association between TAGs containing regulatory loci and tissue-specific expressed genes, we conduct tissue specific enrichment analysis (TSEA) for regulatory and non-regulatory TAGs by using *deTS* software GTEx panel (27). Genes with top 5% highest t-statistics were regarded as tissue-specific expressed genes.

## RESULTS

### Overview of chromatin features in epigenome compendium

**Figure 1.**
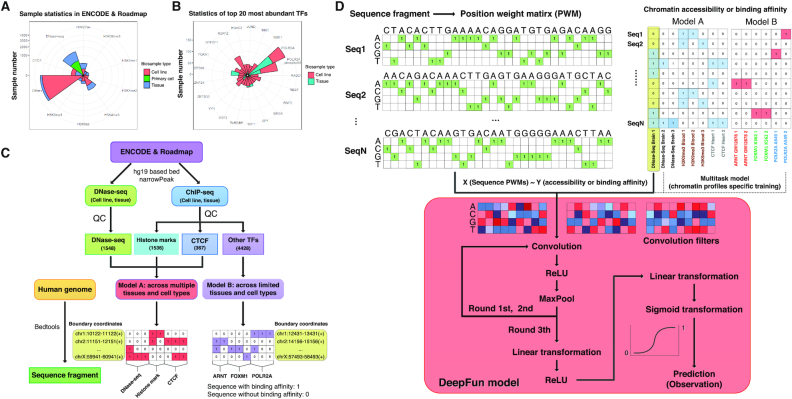
Data process and framework for DeepFun model construction. (**A**) Summary of epigenomic features: DNA accessibility, histone marks and transcription factors. (**B**) Summary of epigenomic features of the top 20 most abundant transcription factors. (**C**) Data process pipeline. (**D**) Framework of DeepFun, a tissue and cell type specific, sequence-based convolutional neural network (CNN) model.

With the increasing availability of epigenetics measurements, we curated comprehensive chromatin features from ENCODE and Roadmap (Supplementary Table S1), spanning 1548 DNase-seq (accessible chromatin), 1536 histone modification marks and 4795 TFs binding profiles. Among these features, 2371 were in different tissue types, while the remaining 5508 were in different primary cells or cell lines. These features were summarized in Figure 1A, B. According to the functional category and completeness, we classified these assays into two tiers (Figure 1C). Tier 1 assays (model A) included 3451 profiles [DNase-seq (1548), all histone marks (1536) and TF CTCF (367)]. Most marks in model A have at least 50 epigenomic measurements across different tissues or cell lines, which provides the user with the opportunity to further study the impact of genetic variants in a tissue-specific or cell-specific manner. Tier 2 assays (model B) consisted of 4428 measurements across 305 TFs in 66 cell lines, providing the opportunity to extensively investigate the impact of variants to TF binding affinity in a cell-specific fashion. After the removal of technical or biological replicates, DeepFun incorporates a total of 117 DNase-seq, 360 histone modification, and 795 TF binding profiles, representing the true diversity of functional predictions. More detailed information for tier 1 and 2 assays can be found in material and methods.

### DeepFun achieved broader biological space and better performance

For both datasets in tier 1 and 2, the downloaded annotation for peaks were created as 1000 bp genomic intervals to all narrow peaks by extending 500 bp on each side of the midpoint of the peak (Details in methods), according to *basset* configuration, respectively (22). These genomic intervals were regarded as epigenomic active sites for downstream analysis. Overall, models A (model B) produced a set of 2 298 761 (2 400 512) epigenomic active sites with a median of 3.3% (10.2%) peaks present in all measurements. A total of 73.7% (model A) and 76.9% (model B) of human genome regions are bounded by at least one chromatin measurement (Supplementary Table S2). Notably, results from both model A and B are very close to the ENCODE reported estimation, which states that 80% of the human genome is comprised of biochemically active regions (16).

As illustrated in Figure 1D, our framework and the architecture of DeepFun models epigenomic active sites as present (label ‘1’) versus absent (label ‘0’). For each epigenomic active site, we firstly transformed the corresponding sequence fragments into a position weight matrix (PWM), along with binary vectors representing presence (1) or absence (0) of the site in each chromatin feature. Both datasets were regarded as input data of the DeepFun model. Then, the CNN model applies hundreds of convolutional filters to search for motifs along the sequence of epigenomic active sites. This is followed by nonlinear rectifier operation and maximum pooling at multiple resolutions to predict the probability of sequence accessibility in a given profile. All convolutional filters are initialized randomly and then optimized along with the training progress. We trained our models using different parameters in order to improve robustness. For each of them, we applied early stop training to avoid overfitting in the case when the loss in the validation set did not decrease within 12 successive epochs. Overall, these models were terminated between 18 to 30 epochs. Our results showed that the genomic interval in 1000 bp gave the best performance. However, changing the number of filters or changing the filter width did not produce a substantial change in prediction accuracy. Therefore, we applied the same hyper-parameters of the original Basset application (22) for the final model in DeepFun.

**Figure 2.**
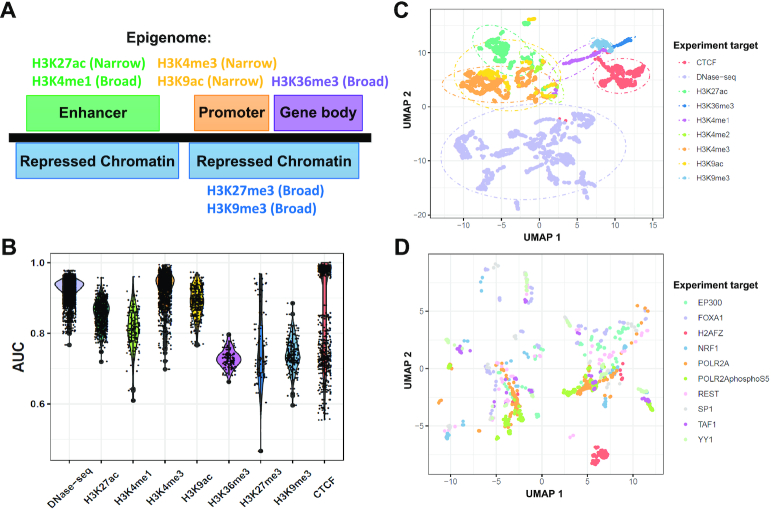
Evaluation of DeepFun model across different experimental targets. (**A**) Overview of histone mark associated regions. (**B**) Evaluaton of model performance. Each violin represents the AUC value of the evaluation for a specific DNase-seq or histone mark feature. (C, D). Uniform manifold approximation and projection (UMAP) analysis of tissue and cell type specificity for the epigenomic profiles for (**C**) DNA accessibility and histone mark, and (**D**) transcription factor.

To synthesize model sensitivity and specificity, we assessed DeepFun performance by using the area under receiver operating characteristic (AUC), which plots the false-positive rate versus the true-positive rate. By this measurement, we show DeepFun is more accurate, achieving a median AUC of 0.933 over all DNase-seq assays (Figure 2B, details AUC for each feature were listed in Supplementary Table S1), compared to 0.895 for original Basset result (22). Simultaneously, our model achieves a mean AUC of 0.872 over all histone mark assays, compared to 0.856 for DeepSEA (21). Although DeepSEA and DeepFun models are different in a number of ways, e.g. not exact same data, the improved AUC suggested that DeepFun may benefit from a dense epigenomic map of the human epigenome (22). On the other hand, we observed that the performance varied depending on the predicted features (Figure 2A). We anticipated it may be correlated with evolutionarily conservative regulatory sequences and experiment quality (7). For example, two promoter region associated histone marks, H3K4me3 and H3K9ac, achieved the highest median AUC at 0.932 and 0.901, followed by two enhancer region associated histone marks, H3K27ac and H3K4me1, with AUC at 0.865 and 0.828. However, for histone marks associated with gene body and repressed chromatin regions, H3K36me3, H3K27me3 and H3K9me3, the median AUC is less than 0.8. Moreover, DeepFun model achieved a median AUC at 0.80 for all TFs assays, ranging from 0.64 (*ZC3H11A*) to 0.98 (*SP4*).

### Marks and TFs specificity on human epigenome

The landscape of epigenomic features in enhancer and promoter regions exhibited stronger mark specificity than tissue specificity in our previous study (19). To get a global landscape of the human epigenome, we used UMAP dimension reduction analysis to visualize the binary vector matrix of epigenomic active sites, mainly including DNase-seq, histone marks, and TF CTCF (insulation, chromatin looping) binding profiles among different tissues or cell lines (Figure 2C). Consistent with our previous study (19), we observed clusters of DNase-seq, and most histone marks were clearly segregated, suggesting there were obvious disagreements in terms of epigenomic modification. The UMAP plot also revealed uniform pattern for those functionally similar histone marks. For example, promoter [H3K4me3 and H3K9ac], enhancer [H3K27ac (active enhancers/promoters) and H3K4me1 (poised enhancers)], gene body and repressed chromatin region enriched marks [H3K36me3 (transcribed), H3K27me3 (polycomb repression), H3K9me3 (heterochromatin)] tended to be clustered together, respectively. Moreover, we conducted UMAP analysis analogously on model B to investigate TF and tissue specificity on human epigenome. As Figure 2D showed, although some TFs, e.g. H2AFZ, formed notable clusters, most other TFs binding profiles were not segregated clearly, indicating most TFs chromatin state tissue specificity are stronger than histone marks.

### Influence of convolutional filter correlates with captured gene expression abundance

The convolution model can automatically extract predictive features from signal sequences through filter scanning (43). We hypothesized these filters would correspond to binding motifs of different TFs, therefore, after converting convolutional filters into PWMs as motifs, we mapped these PWMs to well-known protein binding motifs by using TomTom software (33). Under FDR threshold of 0.1, both model A and B’s 300 convolutional filters captured 108 (36%) and 113 (37.7%) known DNA binding protein in CIS-BP database (Supplementary Figures S1, S2 and Table S3) (33,44). This is slightly lower than the proportion reported in previous studies (i.e. 45%) (22). The higher proportion of unrecognized filters in DeepFun model implied more novel sequence motifs that are not currently represented in CIS-BP database.

**Figure 3.**
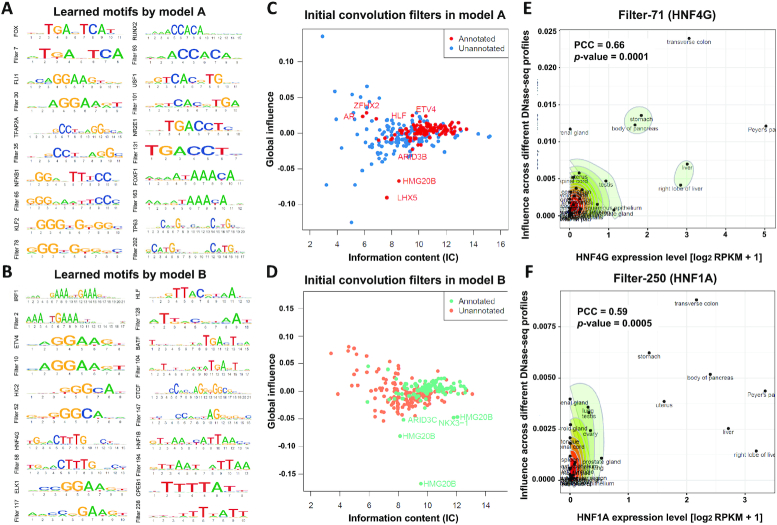
Transcription factor binding motifs learned by DeepFun through its initial convolutional layer learning. (**A, B**) Canonical transcription factor binding motifs identified by DeepFun models A and B. (**C, D**) The initial convolutional layer of DeepFun models A and B identified known and novel sequence motifs. Each dot represents a convolution filter in the initial convolutional layer. The *x* and *y* axes are the information content (IC) and global influence (details in methods). (E, F) Correlation between convolution filter influence score and TF motif captured gene expression levels. Two typical (**E**) filter-71 (HNF4G) and (**F**) filter-250 (HNF4G). The *x* axis is motif analysis (mapping filter-71 to HNF, details in methods) captured TF gene expression level (from RNA-seq data of ENCODE matched tissues). The *y* axis is influence of the same filter across different profiles (only DNA accessibility profiles across different tissues were presented).

As shown in Figure 3A, B, a summary of alignments between first layer convolutional filters and CIS-BP captured motifs. Both models dedicated most filters to comprehensively represent CTCF’s 19-bp-long DNA recognition site, followed by IRF1’s 21-bp-long DNA recognition site (Supplementary Table S3). In addition, as shown in Supplementary Figures S1 and S2, many filters only captured partial coverage of known motifs. To explore each filter's influence score, we nullified each filter on the downstream accessibility or binding activity predictions over all epigenetic signals, which was used to emphasize the importance of the local sequence context of binding motifs that can affect their function (22). From filter information content (IC) and global influence score plots in Figure 3C, D, we note there are a lot of un-annotated filters with a higher influence score.

As the influence score of filter across different features are different, we further investigated the correlation between filter influence score and corresponding motif captured gene expression level in DNase-seq profiles. For example, as shown in Figure 3E, F, filter-71 and filter-250 in colon tissue, stomach and pancreas tissue are predicted with the highest influence score. Interestingly, we observed a significant positive correlation between genes expression levels and influence score: *Pearson correlation coefficient* (PCC) = 0.66 for HNF4G and filter 71 (*P*-value = 1 × 10^−4^) and PCC = 0.59 for HNF1A and filter 250 (*P*-value = 5 × 10^−4^). We conjecture most TF genes are known to regulate development to their active tissue types. To validate our hypothesis, for all 108 captured filters by TomTom software (33) in model A, we calculated the PCC between the influence score of filter and corresponding motif captured gene expression level. Interestingly, one side t-test (*P*-value: 5.9 × 10^−3^) showed the PCC value of high confidence captured filters (FDR_TomTom_ < 10^−4^) are significantly higher than low confidence captured filters (10^−4^ < FDR_TomTom_ < 0.1), indicating the impact of variant across different tissues is correlated variant affected gene expression level.

### DeepFun models identify causal variants in disease-associated tissues

**Figure 4.**
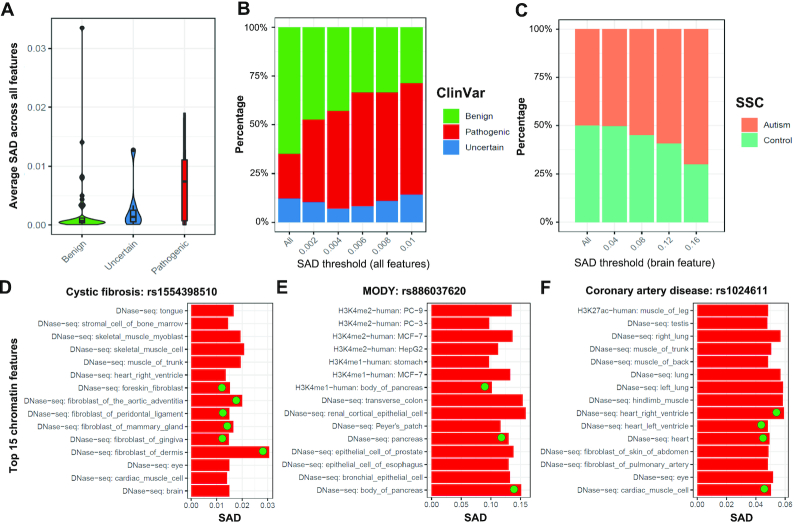
GWAS causal SNPs validation. (**A**) Comparison of the average absolute SAD values among three sets of intergenic variants (benign, uncertain and pathogenic) as annotated in ClinVar database. (**B**) The proportion of ClinVar benign, uncertain and pathogenic variants by the SAD threshold, showing the improvement proportion of pathogenic variants along with SAD threshold improvement. (**C**) The proportion of *de novo* mutations in autism spectrum disorder (ASD) health and patient siblings comparison, by different SAD threshold. Of note, different from the ClinVar variants that were associated with various diseases or phenotypes, here, we only compared the average SAD scores of the non-coding variants over all brain tissues. (D–F) The top 15 chromatin features for three non-coding variants in ClinVar database with the highest SAD values were presented: (**D**) rs1554398510 associated with cystic fibrosis, (**E**) rs886037620 associated with maturity-onset diabetes of the young (MODY), and (**F**) rs1024611 associated with coronary artery disease. We labeled a circle on chromatin features consistent with their disease symptoms.

We applied DeepFun model to evaluate the genetic variants that had been labeled by the ClinVar database (36) with benign, pathogenic, or uncertain functions. For each variant, we defined a SNP Activity Difference (SAD) score (details in methods) to represent its functional impact based on all epigenetic features. Consistent with previous reports (32), the average SAD of most variants were close to zero when clustering all epigenetic features together. Nevertheless, we compared their absolute average SAD scores over DNase-seq signals across all tissues and cell types. As shown in Figure 4A, the median absolute SAD of pathogenic variants (7.4 × 10^−3^) is ten times higher than benign variants (6.5 × 10^−4^), indicating they are more likely to be deleterious mutations than the benign group (Figure 4A). The *P*-value 0.028 from the one side Wilcoxon rank-sum test revealed pathogenic variants had significantly higher average SAD scores than benign variants. Moreover, we found that the percentage of pathogenic variants increases as SAD score increases, while the percentage of benign variants decrease along with SAD threshold improve (Figure 4B).

Human tissues carry out common genetic information, however, tissue and cell-type specific gene expression are distinguished by distinct transcription regulatory programs (45). Variants impact prediction in tissue and cell type specific fashion remains a critical challenge (21,22). Therefore, we evaluated the predicted impact of variants in different features. Firstly, we started with the *de novo* mutations in autism spectrum disorder (ASD) cohort from the Simons Simplex Collection (SSC) in brain tissues (37). The SSC achieved its primary goal to establish a permanent repository of genetic samples from 2600 simplex families. Notably, each family has one child affected by ASD and unaffected parents and siblings. This cohort makes it possible to perform large-scale reliable non-coding *de novo* causal mutations evaluation. As shown in Figure 4C, along with groups of variants defined using increasing SAD thresholds, the percentage of variants in patient siblings increases, while the percentage in health siblings decreases.

We re-evaluated pathogenic variant effects from ClinVar across different epigenomic features (36), especially for 11 pathogenic variants located in intergenic regions (Supplementary Table S4, Supplementary Figure S3). These causal variants are associated with various kinds of diseases. For example, rs1554398510 (chr7: 117315915, C > T) is associated with cystic fibrosis, rs886037620 (chr8: 11331747, G > A) is associated with maturity-onset diabetes of the young, and rs1024611 (chr17: 32579788, A > G) is associated with coronary artery disease. Therefore, we examined their SAD score for different features and presented features ranked within top 15. As Figure 4D showed, most fibroblast tissues related DNase-seq profiles were associated with rs1554398510, especially in fibroblast of dermis. Figure 4E showed both DNase-seq and H3K4me1 profiles in pancreas tissue had strong association with rs886037620. In addition, the impact of rs1024611 was the strongest in heart and cardiac muscle tissue (Figure 4F). Together, the results are consistent with their disease symptoms, suggesting that our model can be insightful to prioritize non-coding causal variants in a tissue specific fashion.

### GWAS lead regulatory loci capture trait-tissue associations systematically

**Figure 5.**
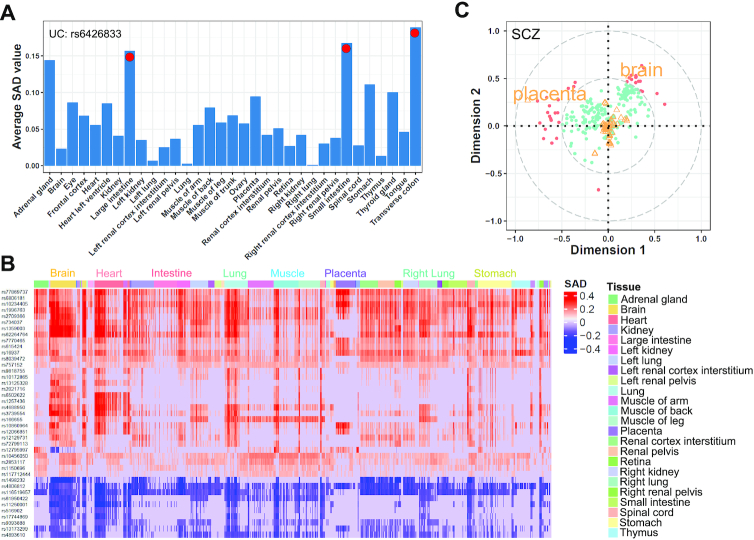
DeepFun captures tissue specific, functional SNPs. (**A**) The average absolute SAD values of DNA accessibility chromatin features for the most associated SNP (rs886037620) with ulcerative colitis (UC) (only tissues with at least eight replicates profiles were presented). (**B**) The SAD values of schizophrenia-associated SNPs (max SAD > 0.1) over different DNA accessibility profiles. (**C**) Canonical correspondence analysis (CCA) (details in methods) of schizophrenia-associated variants SAD scores across different DNase-seq chromatin profiles. As functional variants impact of SAD and source of each DNase-seq profiles (tissue) are assumed to be of unit variance, their projections on the plane reside within a circle of radius 1 centered at the origin. Distance to the center indicates the strength of the association. For clarity, one circle with radius of 0.5 is shown to indicate associations of variants and tissues. Variants (dots) located within or outside of circle with radius of 0.5 were labeled in green and red color, respectively. Different tissue profiles located within or outside of circle with radius of 0.5 were labeled in orange color text or marked as triangle.

To further understand the biological insights, we evaluated lead SNPs from GWAS raw statistics. We started from ulcerative colitis (UC) GWAS summary statistics. UC is a chronic inflammatory disease of the colon with symptoms such as diarrhea and gastrointestinal bleeding (46). A total of 8354 SNP with *P*-value < 10^−8^ were submitted to DeepFun model. We presented all SNPs with max absolute SAD score > 0.1. As Supplementary Figure S4 showed, most SNPs demonstrated tissue specific fashion. The SNP rs6426833, whose GWAS *P*-value = 4.86 × 10^−31^, exhibited the highest SAD value. We further investigated the SAD value of rs6426833 in different tissue chromatin features. As shown in Figure 5A, the top 3 tissues with the highest SAD were transverse colon (averaged SAD value = 0.19), small intestine (0.17) and large intestine (0.16). In addition, we observed several blocks (SNPs cluster) in Supplementary Figure S4 were specific to features in large intestine and small intestine tissue. These results suggested that DeepFun predicted strong functional impact of UC associated SNPs on colon and intestine tissues, which is consistent with the disease symptom associated tissues. Moreover, we found most SNPs clustered together are located in Linkage Disequilibrium regions.

Since SNPs in strong LD with the lead SNP may also achieve a very low p value, we next evaluated schizophrenia (SCZ) GWAS summary statistics by using a less stringent *P*-value < 10^−3^ as a threshold. As SCZ is a brain disorder (27), we only focus on those SNPs with absolute SAD score > 0.1 in brain tissue. In addition, we presented the SAD value of these variants over different tissues in Figure 5B, then applied canonical correspondence analysis (CCA) to explore their functional impact tissue specificity. CCA infers information from two matrices and projects data points into a single embedding space (detail in Materials and Methods). The distance from the center indicates the relation strength and data points that are close to each other show correspondence. For better visualization, we used two-dimensional scatter plots, also known as canonical loading plots, to exhibit the correspondence between variant SAD score and DNase-seq profiles. As Figure 5C shown, the greater the distance from the origin, the stronger the association between variants and tissues. For clarity, one circle with a radius of 0.5 is shown to indicate associations between SCZ associated variants and DNase-seq profiles in different tissues. Although the majority of variants located inside the circle with radius of 0.5, we found several variants are tightly coupled with brain tissues. This reinforced the notion that these polymorphic loci were likely specifically involved in brain functions, which demonstrated our model is capable to predict tissue or cell-specific regulatory loci. Moreover, some polymorphic loci are tightly coupled with ‘placenta’, a tissue derived from fetal cells (47,48). Interestingly, the previous study showed brain developmental stages involved in SCZ disease (49), therefore, we further investigated human brain spatiotemporal expression profiles analysis from BrainSpan (50). Interestingly, based on WGCNA results (details in supplementary material), we found brain prenatal stage-specific expression genes are enriched in ‘uterus’ tissue (Supplementary Figure S5). While previous study also reported SCZ related genes tend to highly express during prenatal development (49). Overall, our approach demonstrated non-coding variant evaluation on a comprehensive epigenetic features enable us to capture the trait-tissue associations systematically.

### CNN on dense epigenomic maps refine GWAS regulatory mechanism loci

**Figure 6.**
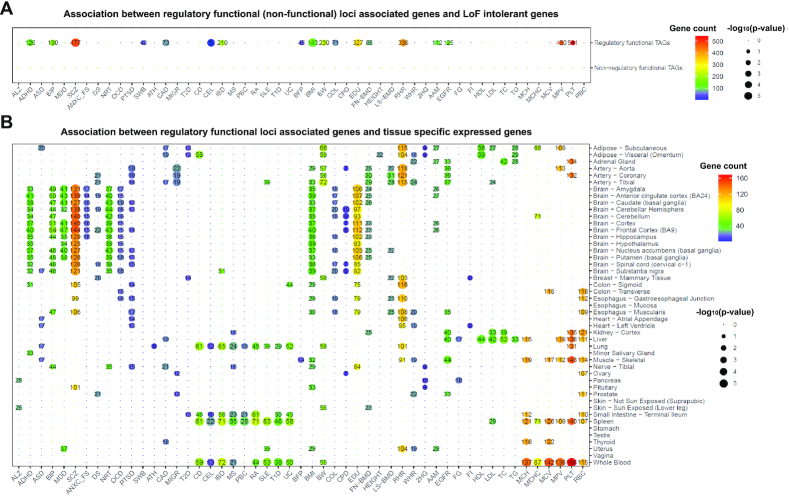
Comparison between 51 traits regulatory and non-regulatory loci associated genes. (**A**) Fisher's exact test of the association between regulatory (non-regulatory) TAGs and LoF intolerant genes (ExAC pLI > 0.9) for each trait. (**B**) Tissue-specific enrichment analysis of 51 TAGs containing regulatory loci. The heatmap only shows the significant trait-tissue associations by *P*-value < 0.05, the shared gene count between trait regulatory loci associated genes and tissue-specific expressed genes were labeled on thie figure.

Epigenetic features provide insights for complex traits interpretation. We downloaded 51 publicly available GWAS summary statistics (Details in Supplementary Table S5) and selected SNPs with GWAS *P*-value < 10^−3^, leading to a total of 2 039 160 variants (range from asthma: 1264 to mean red blood cell volume: 166,322) for potential regulatory loci prediction. After predicting the maximum SAD score across all chromatin features, we distinguish these loci as potential regulatory loci (max SAD > 0.1 or < –0.1), or non-regulatory loci. Due to linkage disequilibrium, DeepFun approach generally lacks the resolution to pinpoint real causal genomic variants. Therefore, we mapped regulatory loci to trait-associated genes (TAGs) through assigning SNPs to its up/down streams genes by Pascal (detail see methods) (41). On the other hand, we downloaded ExAC pLI score of genes, which indicates the probability of intolerant to a loss of function (LoF) from ExAC project (42). To accurately estimate the overlap between regulatory TAGs and LoF intolerant genes (ExAC pLI > 0.9), we used Fisher's exact test (FET) to investigate each trait separately. As shown in Figure 6A, in 18 of 51 TAGs containing regulatory loci are significantly overlapped with LoF intolerant genes at FET *P*-value < 0.05, especially for schizophrenia and education attainment with *P*-value < 1 × 10^−5^. However, all 51 traits show no significant associations between non-regulatory TAGs and LoF intolerant genes. For example, although previous study showed schizophrenia common alleles are enriched in mutation intolerant genes (51), our results demonstrated only SCZ associated genes containing regulatory loci (*P*-value = 5.5 × 10^−9^) are overlapped with LoF intolerant genes.

Identifying the tissue and cell type context is a critical step to interpret genetic variants and understand the insights of disease origin (15). We recently developed an *R* package *deTS* (27) and demonstrated its robustness on most TAGs that are enriched in the trait-related tissues. For example, most neuropsychiatric diseases were enriched in brain tissues, immune-related traits in blood and spleen, and metabolic traits in liver tissue (27). The genetic variants tend to manifest their impacts in the trait-related tissues. We conducted in house tissue-specific enrichment analysis to compare the enriched tissues of regulatory and non-regulatory TAGs for each trait (15,52). As shown in Figure 6B and Supplementary Figure S6, although most regulatory and non-regulatory TAGs show consistent association patterns, non-regulatory TAGs have a broad weak enrichment and tend to be enriched in non-traits-relevant tissue (e.g. brain tissue). More importantly, we observed a lot of novel trait-tissue associations for genes containing regulatory loci (Figure 6B), e.g. asthma genes associated with the lung (asthma associated genetic variants were found to be eQTLs in lung (53)); age at menarche genes associated with the uterus (menstruation is the process after estrogen and progestogen stimulate growth and vascularity of the endometrium in the uterus. Therefore, estrogen and progesterone receptor expression in the human uterus might be associated with the age at menarche (54)), body fat percentage genes associated with muscle (previous study shows that muscle sympathetic nerve activity is related to the body fat distribution (55)), fasting glucose associated with liver (the liver can store and manufacture glucose and helps to keep your circulating blood sugar levels and other body fuels steady and constant (56)), and 2-h glucose associated with pancreas tissue (pancreatic islet could generate the hormone insulin, which could maintain the glucose homeostasis (57)). However, these associations could not be captured by non-regulatory TAGs. Therefore, our study demonstrated that interpreting GWAS functional consequence of genetic variants through CNN model cannot only improve the possibility to prioritize real causal variants, but also can provide novel insights for better decoding disease-relevant tissues and etiology.

## DISCUSSION

In the past decade, genome-wide association studies (GWAS) and whole-genome sequencing (WGS) analysis of family trios have generated rich resources of genetic variants and *de novo* mutations associated with monogenic or complex diseases (37,40). However, more than 90% of genetic variants reported are located in non-coding regions (4). Although several deep learning-based models have exhibited remarkable advantages (21,22), interpreting the genetic susceptibility of these variants remains a big challenge due to the distinct transcription regulatory programs (7). In this study, we present the most comprehensive chromatin maps of human epigenome, encompassing 7879 datasets, including both DNase-seq and ChIP-seq data for different histone marks and TFs, which greatly expand the biological space covered by previous reference epigenome maps. Although many of these are simply replicates (58), they can also help users distinguish reproducible results from accidental results. Our broader biological space provides valuable implications for both capturing gene-regulatory elements of an increased set of tissue-specific measurements, and for annotating gene-regulatory variants across a broader biological spectrum for traits and disease phenotypes, which was previously uncaptured (59). With DeepFun, researchers can also perform *in silico* saturated mutagenesis analysis in their interested cell type and simultaneously learn the influence of every mutation on chromatin accessibility or TF binding activity (Supplementary Materials and Figure S7).

The application of deep learning methods to characterize the regulatory potential of non-coding variants has been a subject of interest in recent years (6,21,22). Deep learning models exhibited great advantage when dealing with larger data set (60). In this work, based on dense epigenomic maps, we presented a tissue and cell type specific CNN model, which can be widely used for prioritizing variants in non-coding regions. By applying our DeepFun model, we observed potential casual variants can be well distinguished from multiple examples, such as ClinVar (36) and Simons Simplex Collection (SSC) cohort (37). Moreover, CNN-prioritized variants provide a powerful way for dissecting causal variants in a tissue- or cell-specific manner. Therefore, we anticipate our work will be a valuable approach for the further refinement of GWAS association signals. To our knowledge, DeepFun model is superior to previous models in three ways. Firstly, DeepFun greatly expanded the biological space covered by previous reference epigenomic maps, which surpasses previous reference maps in scope, scale, and coverage of biological space. Moreover, training on this broader biological space reveals that DeepFun exhibits better performance than previous Basset model (22). Secondly, as chromatin states are dynamic across different tissue types, the assessment of the impact of variants under specific tissue type is necessary for downstream functional investigation (7). The extended profiles collected for the DeepFun model not only facilitate the systematical assessment of the impact of variants in a specific tissue or cell types (tier 1), but also provide the opportunity to extensively interpreted potential target genes affected by functional variants (tier 2). Finally, we keep redundant epigenomic profiles rather than merged technical or biological replicates of one profile, which significantly improved prediction robustness of noise signals.

Trait-associated tissues serve as promising gauges for identification and interpretation of causal variants. We apply DeepFun to decipher non-coding variant effects on complex disease tissue. To do so, we systematically evaluated 51 GWAS lead SNPs. We filtered ‘hitchhiker’ SNPs with low absolute SAD scores. Then we classified TAGs into two categories: regulatory and non-regulatory TAGs. Interestingly, tissue specific enrichment analysis of regulatory TAGs revealed numerous novel associations, e.g. asthma enriched in lung, age at menarche enriched in uterus, body fat percentage enriched in muscle tissue. In most complex diseases, the association between traits and tissues is not always straightforward because in some cases, multiple tissues may be implicated in the etiology of the disease (6,59). In this study, we show several attractive applications for DeepFun. We demonstrate the interpretation of causal variants. We show how a deep-learning-based model trained on dense, rich, and high-resolution epigenomic annotations can provide an important basis for studying the common and distinct components of disease-comorbidity relationships. We believe the focus on regulatory loci will greatly prompt the establishment of the trait-tissue association map, which is of utmost importance to understand the insights of disease etiology and to advance post GWAS analyses (61,62).

There are several ways in which we can further improve on our methods in the future. First, we may integrate quantitative epigenomic signals instead of binary vector, thereby upgrading DeepFun to predict more accurate quantitative signals (24). However, appropriate normalization of the data across different samples is necessary for eliminating technical bias due to experiment design. Second, we expect to collect more epigenomic annotations or use computational methodology, such as tensor-based imputation, to complete the epigenomic data in diverse missing experiments (63). So far, the ChIP-seq data of TF over different cell type measurements remain highly incomplete. Therefore, interpreting the impact of a functional variant on a TF still remains challenging in the case when suitable tissue chromatin feature is missing. Thirdly, to improve the model structure, we will explore novel architecture algorithms for more effective deconvolution of sequence signals. For example, we may initialize half of convolutional filters with known binding motifs (7), or use dilated convolution filters strategy, thereby capturing distinct sequence motifs that would not be identified by regular convolution filters (24). Fourth, our approach did not take the genotype of different individuals into consideration (32). Lastly, we expect to analyze non-human datasets, as training models on multiple species, e.g. mouse or primate data, is an alternative way to improve prediction accuracy (25). On the other hand, in this work, we utilized Pascal to aggregate the GWAS signals to the nearby genes and conducted the tissue-specific enrichment analysis to further explore the tissue-specificity of these GWAS surrogate genes, which might not fully represent the long-range regulations. There are several alternative methods that could directly obtain the traits-associated tissues utilizing the tissue-specific eQTL information (64,65). Moreover, we can further compare the performance of DeepFun with some machine learning based model, such as DIVAN (58). Despite these challenges, we demonstrated that a CNN-based model trained with dense, rich, and high-resolution epigenomic annotations is very effective at prioritizing non-coding regulatory variants from GWAS data. Moreover, for most complex diseases, a focus on true regulatory variants against background signals would be an alternative approach to dissect the map of trait-tissue associations.

## DATA AVAILABILITY

All the data generated or analyzed in this study is available from the authors upon request. We deposit DeepFun pre-trained models at Github https://github.com/bsml320/DeepFun.

## Supplementary Material

gkaa1137_Supplemental_Files
