# Supplementary material for: Predicting regulatory variants using a dense epigenomic mapped CNN model elucidated the molecular basis of trait-tissue associations
Source: Nucleic Acids Res. 2020 Dec 9;49(1):53–66. doi: 10.1093/nar/gkaa1137 (PMC7797043; doi:10.1093/nar/gkaa1137)
Supplement: gkaa1137_Supplemental_Files [file gkaa1137_supplemental_files.zip › Supplementary Figure S3.pdf]

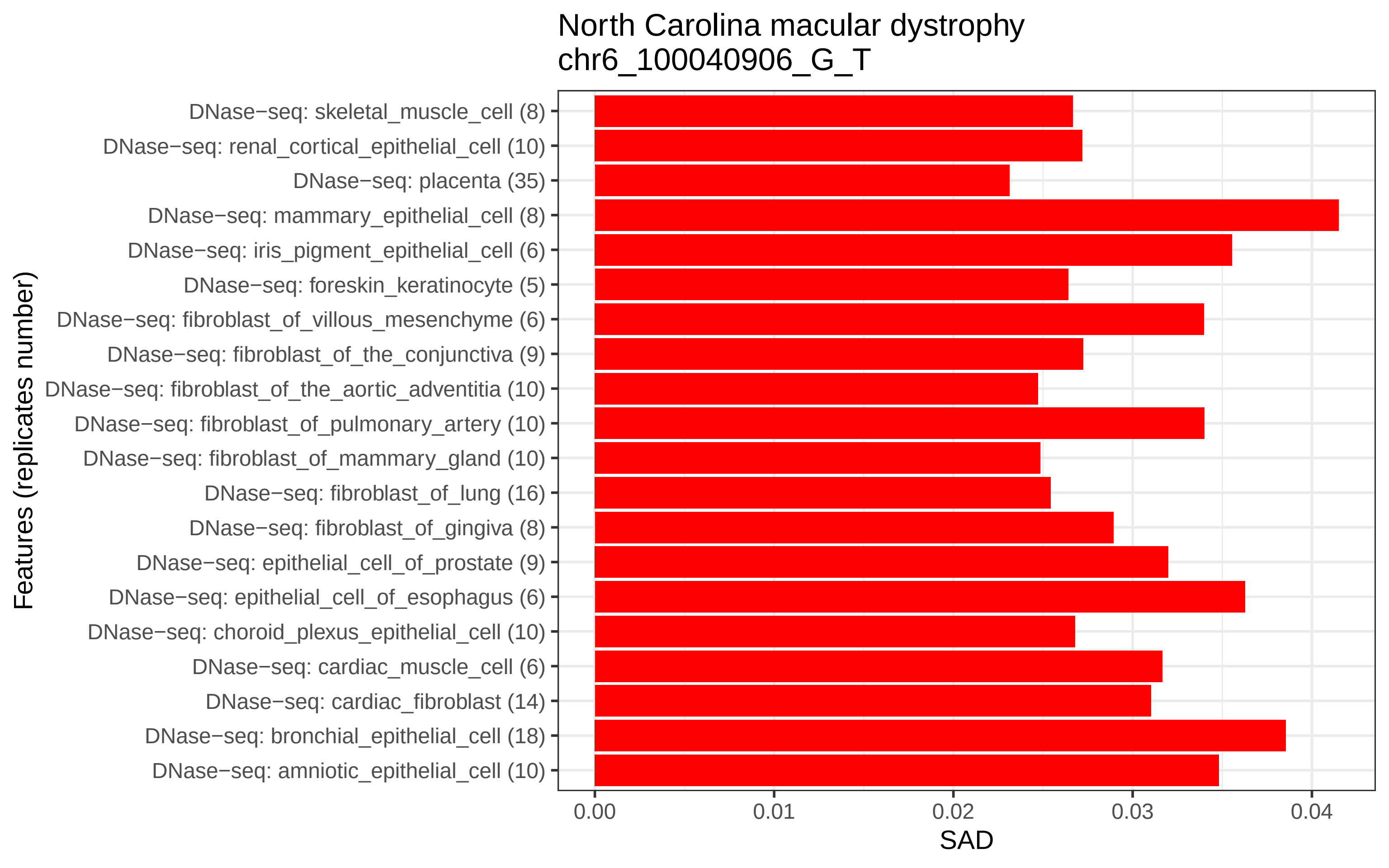
North Carolina macular dystrophy
chr6_100040906_G_T
DNase−seq: skeletal_muscle_cell (8)
DNase−seq: renal_cortical_epithelial_cell (10)
DNase−seq: placenta (35)
DNase−seq: mammary_epithelial_cell (8)
DNase−seq: iris_pigment_epithelial_cell (6)
DNase−seq: foreskin_keratinocyte (5)
DNase−seq: fibroblast_of_villous_mesenchyme (6)
DNase−seq: fibroblast_of_the_conjunctiva (9)
DNase−seq: fibroblast_of_the_aortic_adventitia (10)
DNase−seq: fibroblast_of_pulmonary_artery (10)
DNase−seq: fibroblast_of_mammary_gland (10)
DNase−seq: fibroblast_of_lung (16)
DNase−seq: fibroblast_of_gingiva (8)
DNase−seq: epithelial_cell_of_prostate (9)
DNase−seq: epithelial_cell_of_esophagus (6)
DNase−seq: choroid_plexus_epithelial_cell (10)
DNase−seq: cardiac_muscle_cell (6)
DNase−seq: cardiac_fibroblast (14)
DNase−seq: bronchial_epithelial_cell (18)
DNase−seq: amniotic_epithelial_cell (10)
Features (replicates number)
0.00
0.01
0.02
0.03
0.04
SAD

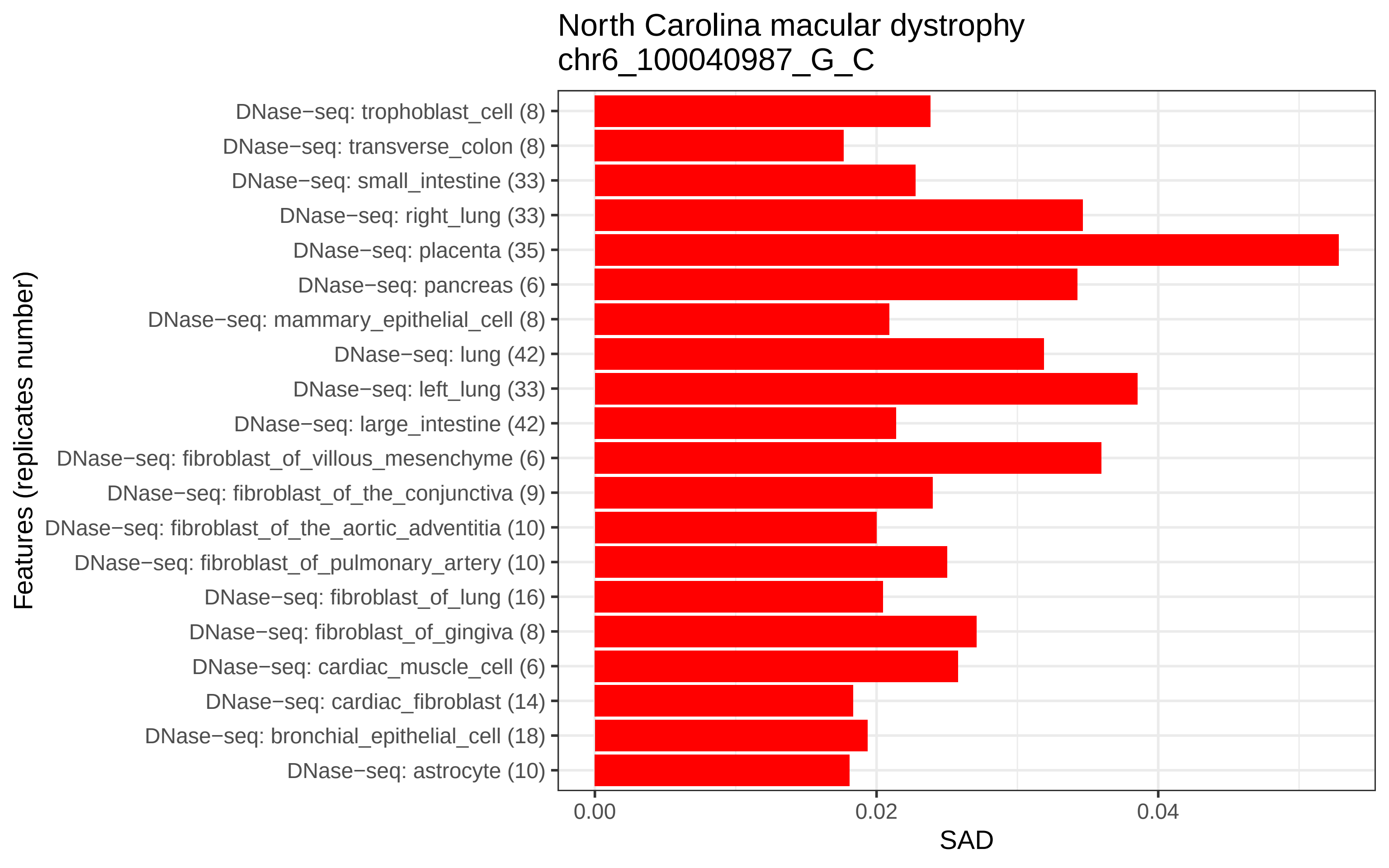
North Carolina macular dystrophy
chr6_100040987_G_C
Features (replicates number)
DNase−seq: trophoblast_cell (8)
DNase−seq: transverse_colon (8)
DNase−seq: small_intestine (33)
DNase−seq: right_lung (33)
DNase−seq: placenta (35)
DNase−seq: pancreas (6)
DNase−seq: mammary_epithelial_cell (8)
DNase−seq: lung (42)
DNase−seq: left_lung (33)
DNase−seq: large_intestine (42)
DNase−seq: fibroblast_of_villous_mesenchyme (6)
DNase−seq: fibroblast_of_the_conjunctiva (9)
DNase−seq: fibroblast_of_the_aortic_adventitia (10)
DNase−seq: fibroblast_of_pulmonary_artery (10)
DNase−seq: fibroblast_of_lung (16)
DNase−seq: fibroblast_of_gingiva (8)
DNase−seq: cardiac_muscle_cell (6)
DNase−seq: cardiac_fibroblast (14)
DNase−seq: bronchial_epithelial_cell (18)
DNase−seq: astrocyte (10)
0.00
0.02
0.04
SAD

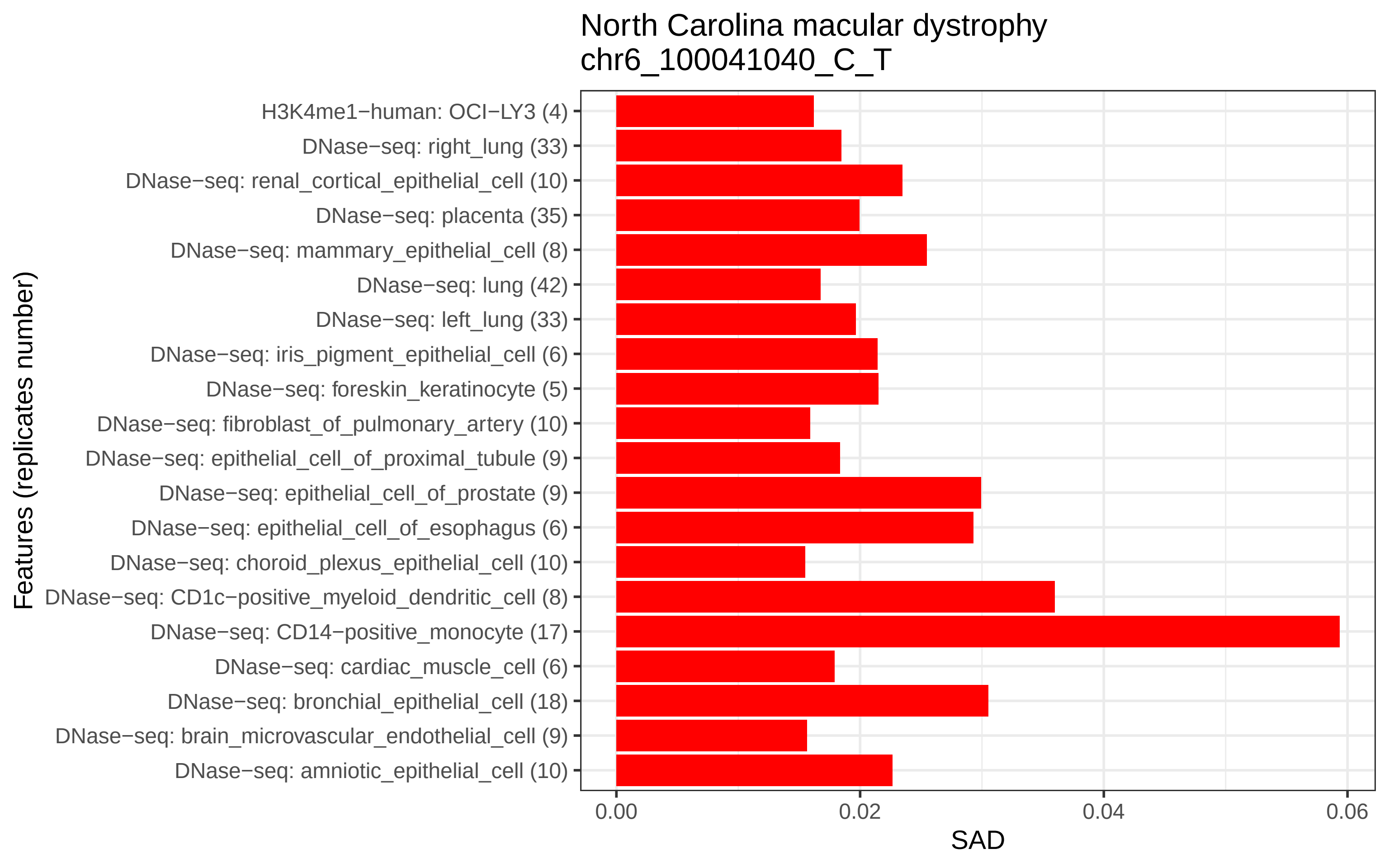

North Carolina macular dystrophy
chr6_100041040_C_T
H3K4me1−human: OCI−LY3 (4)
DNase−seq: right_lung (33)
DNase−seq: renal_cortical_epithelial_cell (10)
DNase−seq: placenta (35)
DNase−seq: mammary_epithelial_cell (8)
DNase−seq: lung (42)
DNase−seq: left_lung (33)
DNase−seq: iris_pigment_epithelial_cell (6)
DNase−seq: foreskin_keratinocyte (5)
DNase−seq: fibroblast_of_pulmonary_artery (10)
DNase−seq: epithelial_cell_of_proximal_tubule (9)
DNase−seq: epithelial_cell_of_prostate (9)
DNase−seq: epithelial_cell_of_esophagus (6)
DNase−seq: choroid_plexus_epithelial_cell (10)
DNase−seq: CD1c−positive_myeloid_dendritic_cell (8)
DNase−seq: CD14−positive_monocyte (17)
DNase−seq: cardiac_muscle_cell (6)
DNase−seq: bronchial_epithelial_cell (18)
DNase−seq: brain_microvascular_endothelial_cell (9)
DNase−seq: amniotic_epithelial_cell (10)
Features (replicates number)
0.00
0.02
0.04
0.06
SAD

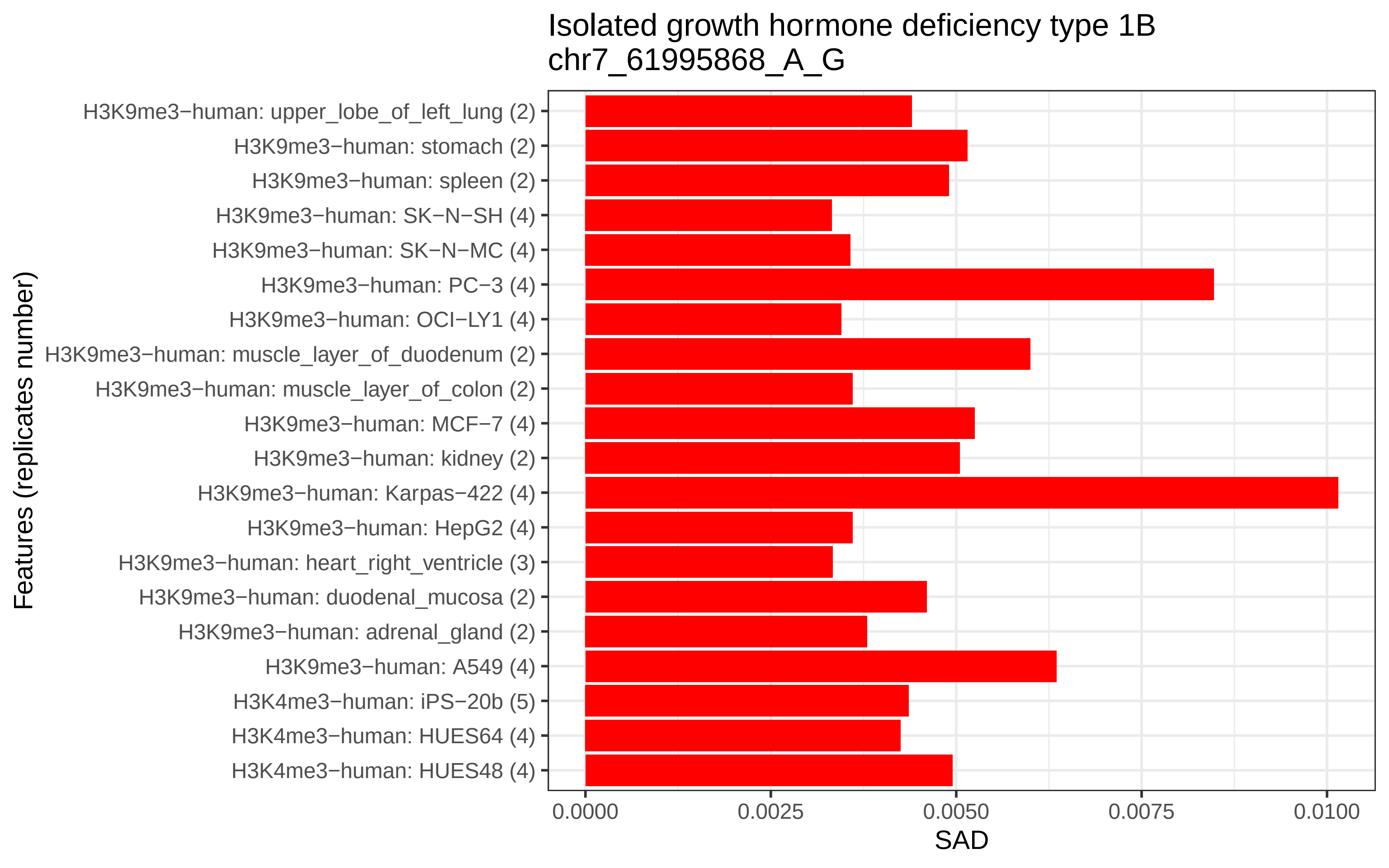
Isolated growth hormone deficiency type 1B
chr7_61995868_A_G
Features (replicates number)
H3K9me3−human: upper_lobe_of_left_lung (2)
H3K9me3−human: stomach (2)
H3K9me3−human: spleen (2)
H3K9me3−human: SK−N−SH (4)
H3K9me3−human: SK−N−MC (4)
H3K9me3−human: PC−3 (4)
H3K9me3−human: OCI−LY1 (4)
H3K9me3−human: muscle_layer_of_duodenum (2)
H3K9me3−human: muscle_layer_of_colon (2)
H3K9me3−human: MCF−7 (4)
H3K9me3−human: kidney (2)
H3K9me3−human: Karpas−422 (4)
H3K9me3−human: HepG2 (4)
H3K9me3−human: heart_right_ventricle (3)
H3K9me3−human: duodenal_mucosa (2)
H3K9me3−human: adrenal_gland (2)
H3K9me3−human: A549 (4)
H3K4me3−human: iPS−20b (5)
H3K4me3−human: HUES64 (4)
H3K4me3−human: HUES48 (4)
0.0000
0.0025
0.0050
0.0075
0.0100
SAD

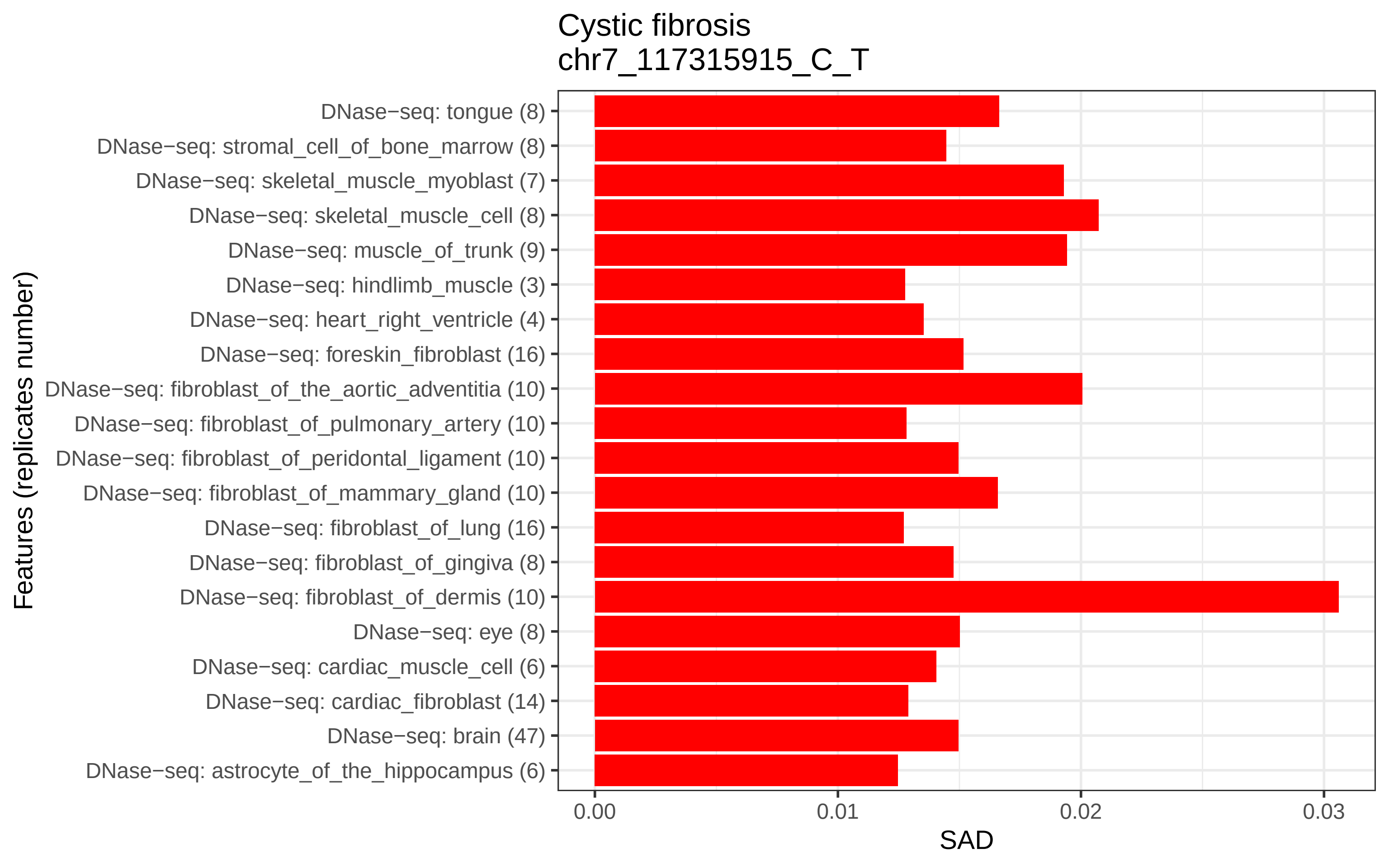
Cystic fibrosis
chr7_117315915_C_T
DNase−seq: tongue (8)
DNase−seq: stromal_cell_of_bone_marrow (8)
DNase−seq: skeletal_muscle_myoblast (7)
DNase−seq: skeletal_muscle_cell (8)
DNase−seq: muscle_of_trunk (9)
DNase−seq: hindlimb_muscle (3)
DNase−seq: heart_right_ventricle (4)
DNase−seq: foreskin_fibroblast (16)
DNase−seq: fibroblast_of_the_aortic_adventitia (10)
DNase−seq: fibroblast_of_pulmonary_artery (10)
DNase−seq: fibroblast_of_peridontal_ligament (10)
DNase−seq: fibroblast_of_mammary_gland (10)
DNase−seq: fibroblast_of_lung (16)
DNase−seq: fibroblast_of_gingiva (8)
DNase−seq: fibroblast_of_dermis (10)
DNase−seq: eye (8)
DNase−seq: cardiac_muscle_cell (6)
DNase−seq: cardiac_fibroblast (14)
DNase−seq: brain (47)
DNase−seq: astrocyte_of_the_hippocampus (6)
Features (replicates number)
0.00
0.01
0.02
0.03
SAD

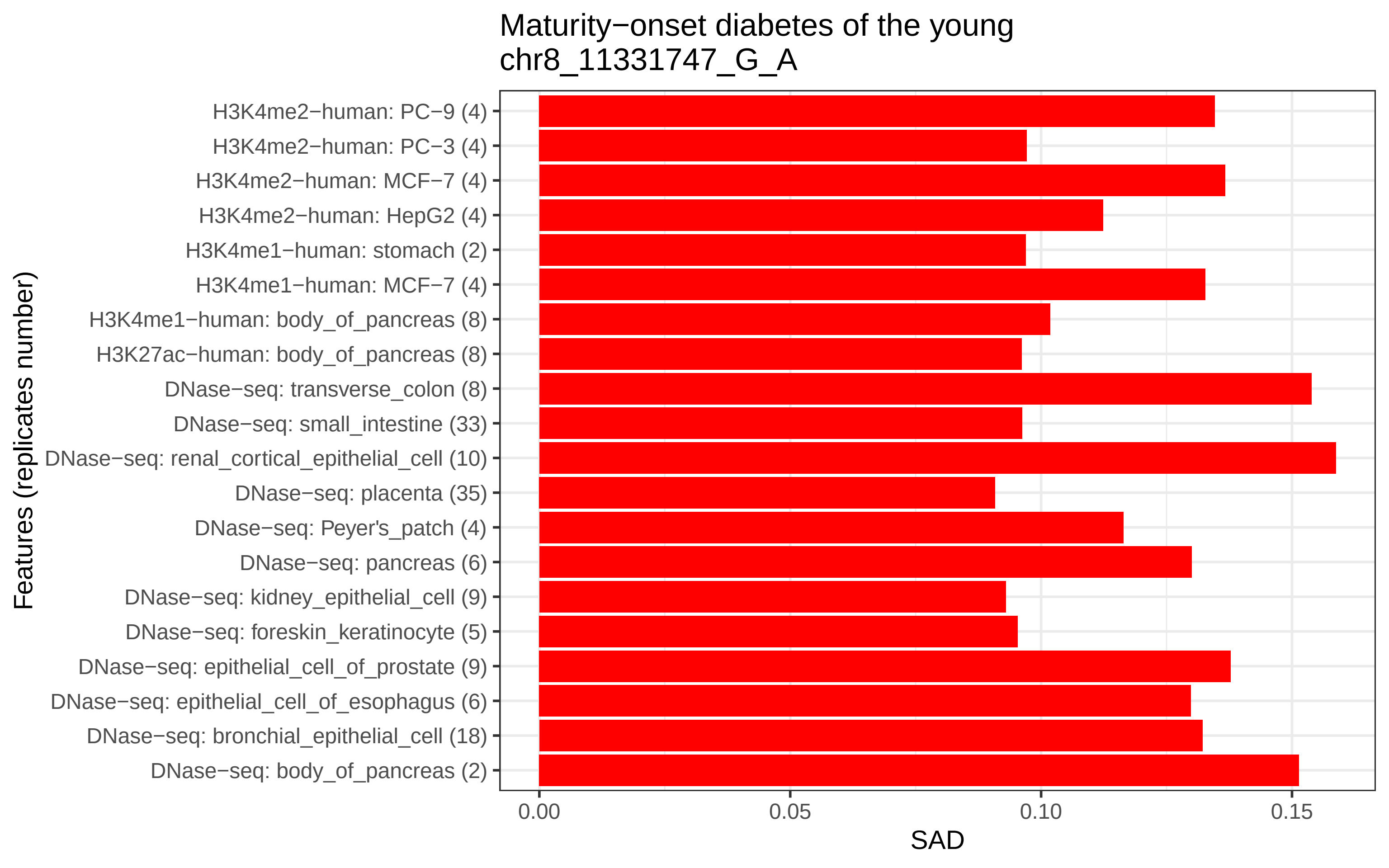
Maturity−onset diabetes of the young
chr8_11331747_G_A
H3K4me2−human: PC−9 (4)
H3K4me2−human: PC−3 (4)
H3K4me2−human: MCF−7 (4)
H3K4me2−human: HepG2 (4)
H3K4me1−human: stomach (2)
H3K4me1−human: MCF−7 (4)
H3K4me1−human: body_of_pancreas (8)
H3K27ac−human: body_of_pancreas (8)
DNase−seq: transverse_colon (8)
DNase−seq: small_intestine (33)
DNase−seq: renal_cortical_epithelial_cell (10)
DNase−seq: placenta (35)
DNase−seq: Peyer's_patch (4)
DNase−seq: pancreas (6)
DNase−seq: kidney_epithelial_cell (9)
DNase−seq: foreskin_keratinocyte (5)
DNase−seq: epithelial_cell_of_prostate (9)
DNase−seq: epithelial_cell_of_esophagus (6)
DNase−seq: bronchial_epithelial_cell (18)
DNase−seq: body_of_pancreas (2)
Features (replicates number)
0.00
0.05
0.10
0.15
SAD

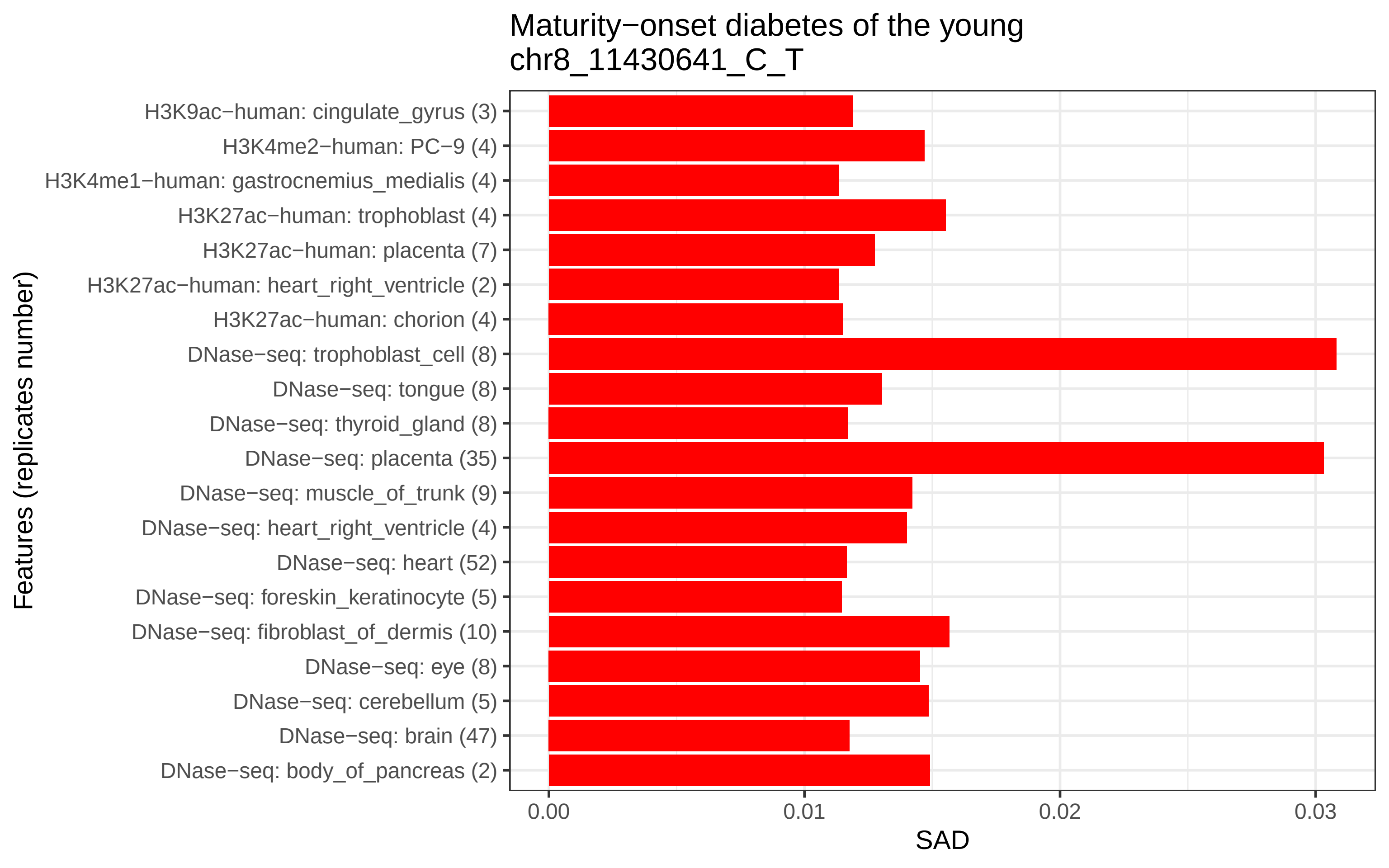

Maturity−onset diabetes of the young
chr8_11430641_C_T
Features (replicates number)
H3K9ac−human: cingulate_gyrus (3)
H3K4me2−human: PC−9 (4)
H3K4me1−human: gastrocnemius_medialis (4)
H3K27ac−human: trophoblast (4)
H3K27ac−human: placenta (7)
H3K27ac−human: heart_right_ventricle (2)
H3K27ac−human: chorion (4)
DNase−seq: trophoblast_cell (8)
DNase−seq: tongue (8)
DNase−seq: thyroid_gland (8)
DNase−seq: placenta (35)
DNase−seq: muscle_of_trunk (9)
DNase−seq: heart_right_ventricle (4)
DNase−seq: heart (52)
DNase−seq: foreskin_keratinocyte (5)
DNase−seq: fibroblast_of_dermis (10)
DNase−seq: eye (8)
DNase−seq: cerebellum (5)
DNase−seq: brain (47)
DNase−seq: body_of_pancreas (2)
0.00
0.01
0.02
0.03
SAD

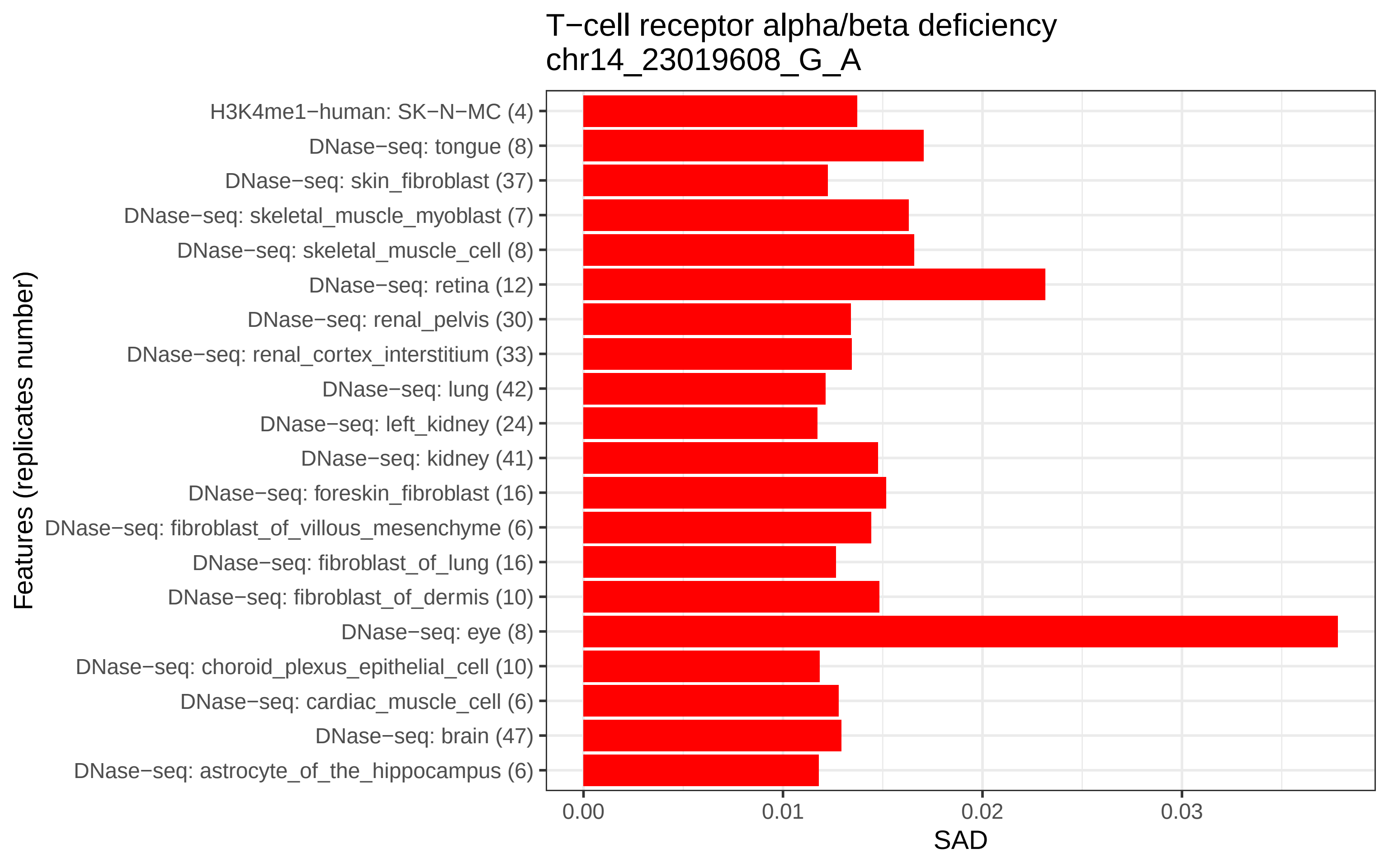
T−cell receptor alpha/beta deficiency
chr14_23019608_G_A
H3K4me1−human: SK−N−MC (4)
DNase−seq: tongue (8)
DNase−seq: skin_fibroblast (37)
DNase−seq: skeletal_muscle_myoblast (7)
DNase−seq: skeletal_muscle_cell (8)
DNase−seq: retina (12)
DNase−seq: renal_pelvis (30)
DNase−seq: renal_cortex_interstitium (33)
DNase−seq: lung (42)
DNase−seq: left_kidney (24)
DNase−seq: kidney (41)
DNase−seq: foreskin_fibroblast (16)
DNase−seq: fibroblast_of_villous_mesenchyme (6)
DNase−seq: fibroblast_of_lung (16)
DNase−seq: fibroblast_of_dermis (10)
DNase−seq: eye (8)
DNase−seq: choroid_plexus_epithelial_cell (10)
DNase−seq: cardiac_muscle_cell (6)
DNase−seq: brain (47)
DNase−seq: astrocyte_of_the_hippocampus (6)
Features (replicates number)
0.00
0.01
0.02
0.03
SAD

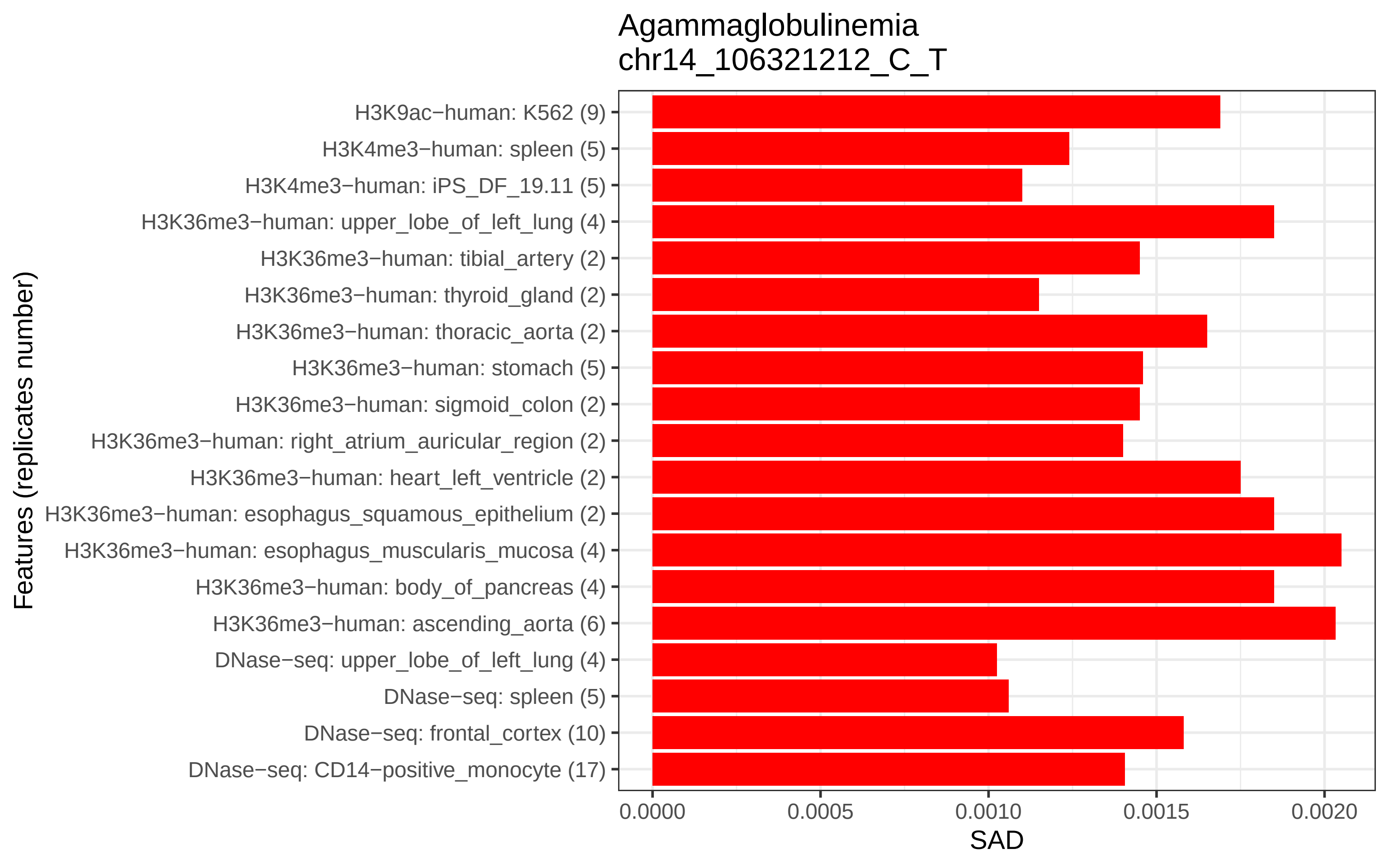

Agammaglobulinemia
chr14_106321212_C_T
Features (replicates number)
H3K9ac−human: K562 (9)
H3K4me3−human: spleen (5)
H3K4me3−human: iPS_DF_19.11 (5)
H3K36me3−human: upper_lobe_of_left_lung (4)
H3K36me3−human: tibial_artery (2)
H3K36me3−human: thyroid_gland (2)
H3K36me3−human: thoracic_aorta (2)
H3K36me3−human: stomach (5)
H3K36me3−human: sigmoid_colon (2)
H3K36me3−human: right_atrium_auricular_region (2)
H3K36me3−human: heart_left_ventricle (2)
H3K36me3−human: esophagus_squamous_epithelium (2)
H3K36me3−human: esophagus_muscularis_mucosa (4)
H3K36me3−human: body_of_pancreas (4)
H3K36me3−human: ascending_aorta (6)
DNase−seq: upper_lobe_of_left_lung (4)
DNase−seq: spleen (5)
DNase−seq: frontal_cortex (10)
DNase−seq: CD14−positive_monocyte (17)
0.0000
0.0005
0.0010
0.0015
0.0020
SAD

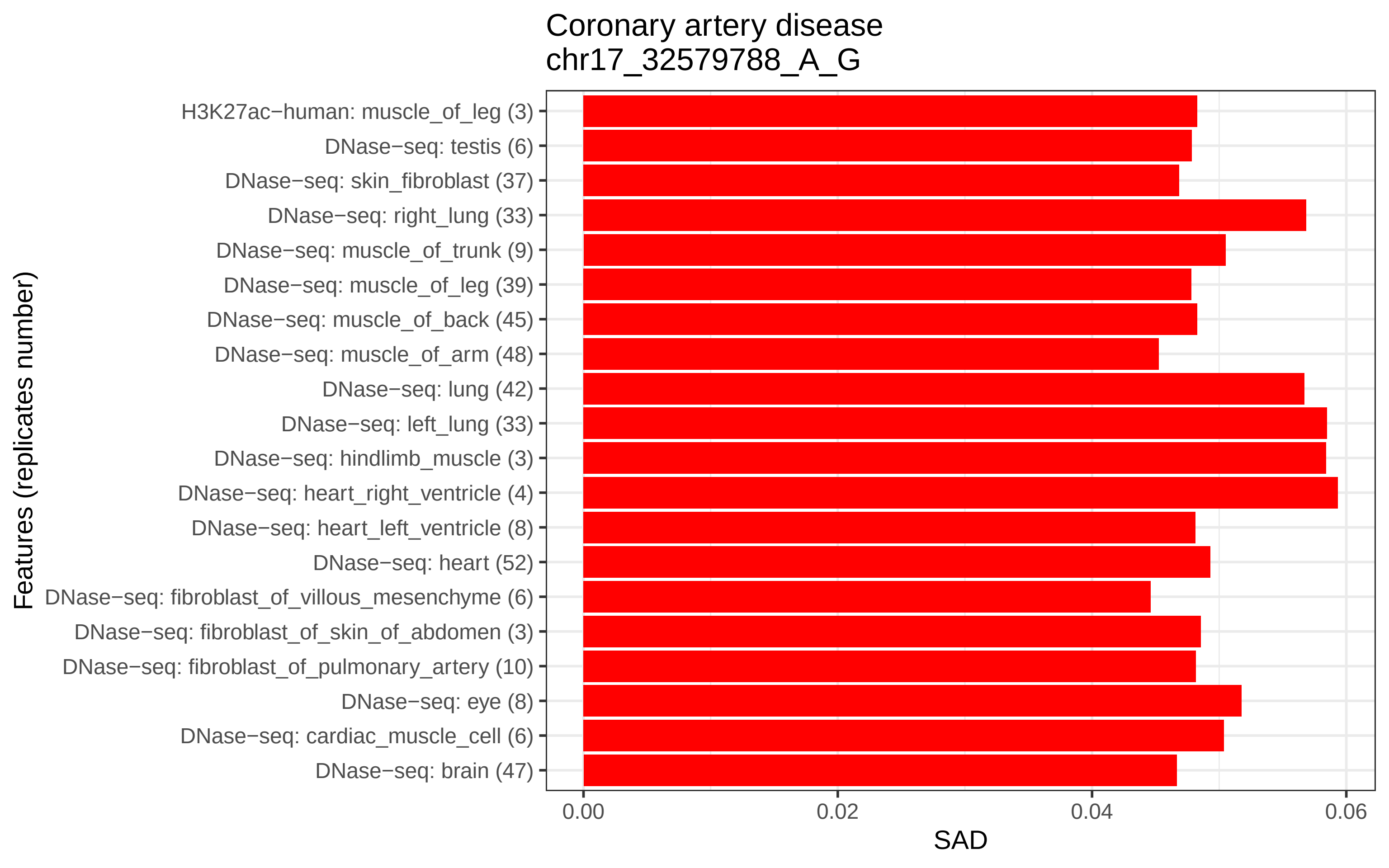
Coronary artery disease
chr17_32579788_A_G
H3K27ac−human: muscle_of_leg (3)
DNase−seq: testis (6)
DNase−seq: skin_fibroblast (37)
DNase−seq: right_lung (33)
DNase−seq: muscle_of_trunk (9)
DNase−seq: muscle_of_leg (39)
DNase−seq: muscle_of_back (45)
DNase−seq: muscle_of_arm (48)
DNase−seq: lung (42)
DNase−seq: left_lung (33)
DNase−seq: hindlimb_muscle (3)
DNase−seq: heart_right_ventricle (4)
DNase−seq: heart_left_ventricle (8)
DNase−seq: heart (52)
DNase−seq: fibroblast_of_villous_mesenchyme (6)
DNase−seq: fibroblast_of_skin_of_abdomen (3)
DNase−seq: fibroblast_of_pulmonary_artery (10)
DNase−seq: eye (8)
DNase−seq: cardiac_muscle_cell (6)
DNase−seq: brain (47)
Features (replicates number)
0.00
0.02
0.04
0.06
SAD

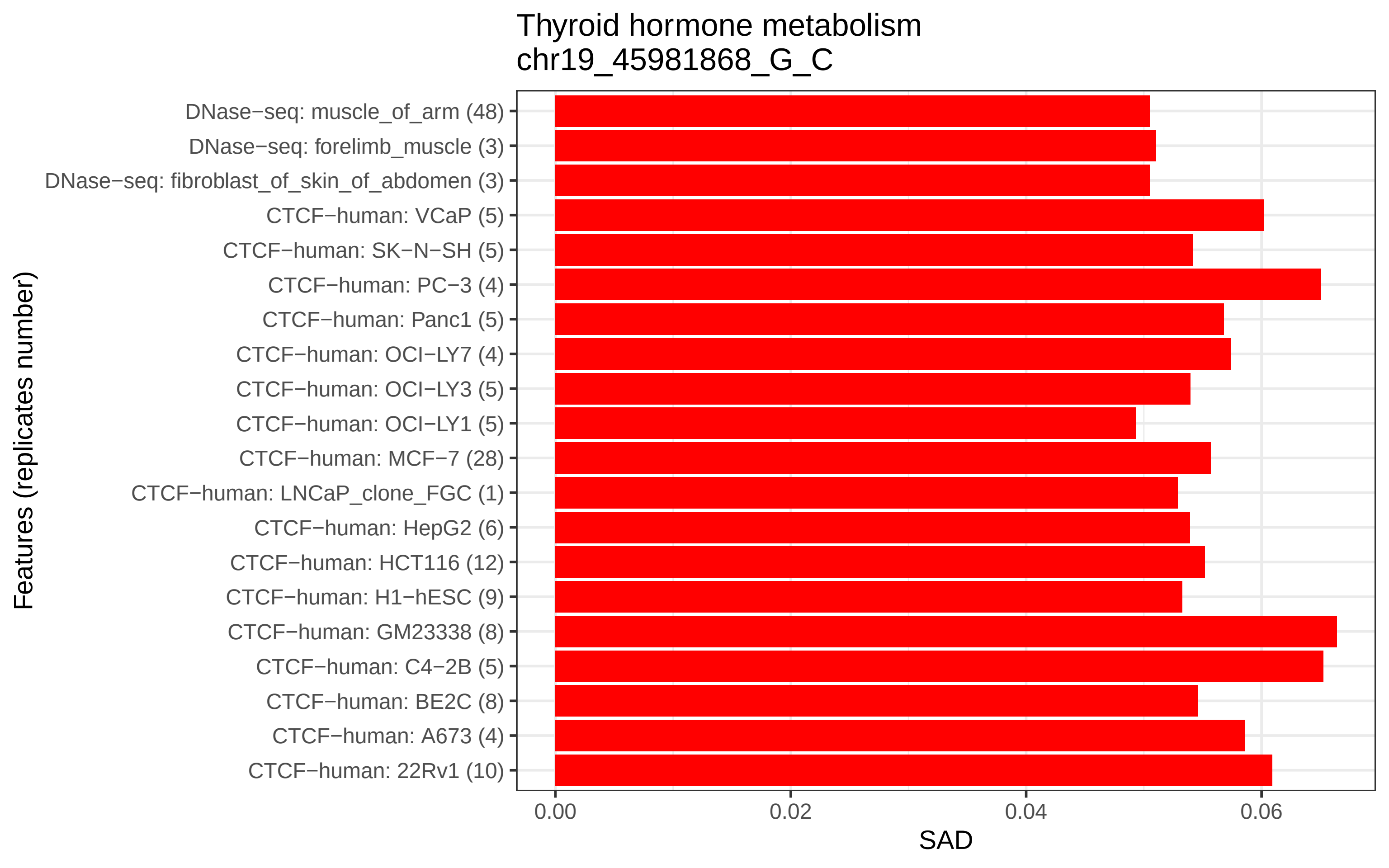
Thyroid hormone metabolism
chr19_45981868_G_C
DNase−seq: muscle_of_arm (48)
DNase−seq: forelimb_muscle (3)
DNase−seq: fibroblast_of_skin_of_abdomen (3)
CTCF−human: VCaP (5)
CTCF−human: SK−N−SH (5)
CTCF−human: PC−3 (4)
CTCF−human: Panc1 (5)
CTCF−human: OCI−LY7 (4)
CTCF−human: OCI−LY3 (5)
CTCF−human: OCI−LY1 (5)
CTCF−human: MCF−7 (28)
CTCF−human: LNCaP_clone_FGC (1)
CTCF−human: HepG2 (6)
CTCF−human: HCT116 (12)
CTCF−human: H1−hESC (9)
CTCF−human: GM23338 (8)
CTCF−human: C4−2B (5)
CTCF−human: BE2C (8)
CTCF−human: A673 (4)
CTCF−human: 22Rv1 (10)
Features (replicates number)
0.00
0.02
0.04
0.06
SAD
