# Supplementary material for: Predicting regulatory variants using a dense epigenomic mapped CNN model elucidated the molecular basis of trait-tissue associations
Source: Nucleic Acids Res. 2020 Dec 9;49(1):53–66. doi: 10.1093/nar/gkaa1137 (PMC7797043; doi:10.1093/nar/gkaa1137)
Supplement: gkaa1137_Supplemental_Files [file gkaa1137_supplemental_files.zip › Supplementary Figure S4.pdf]

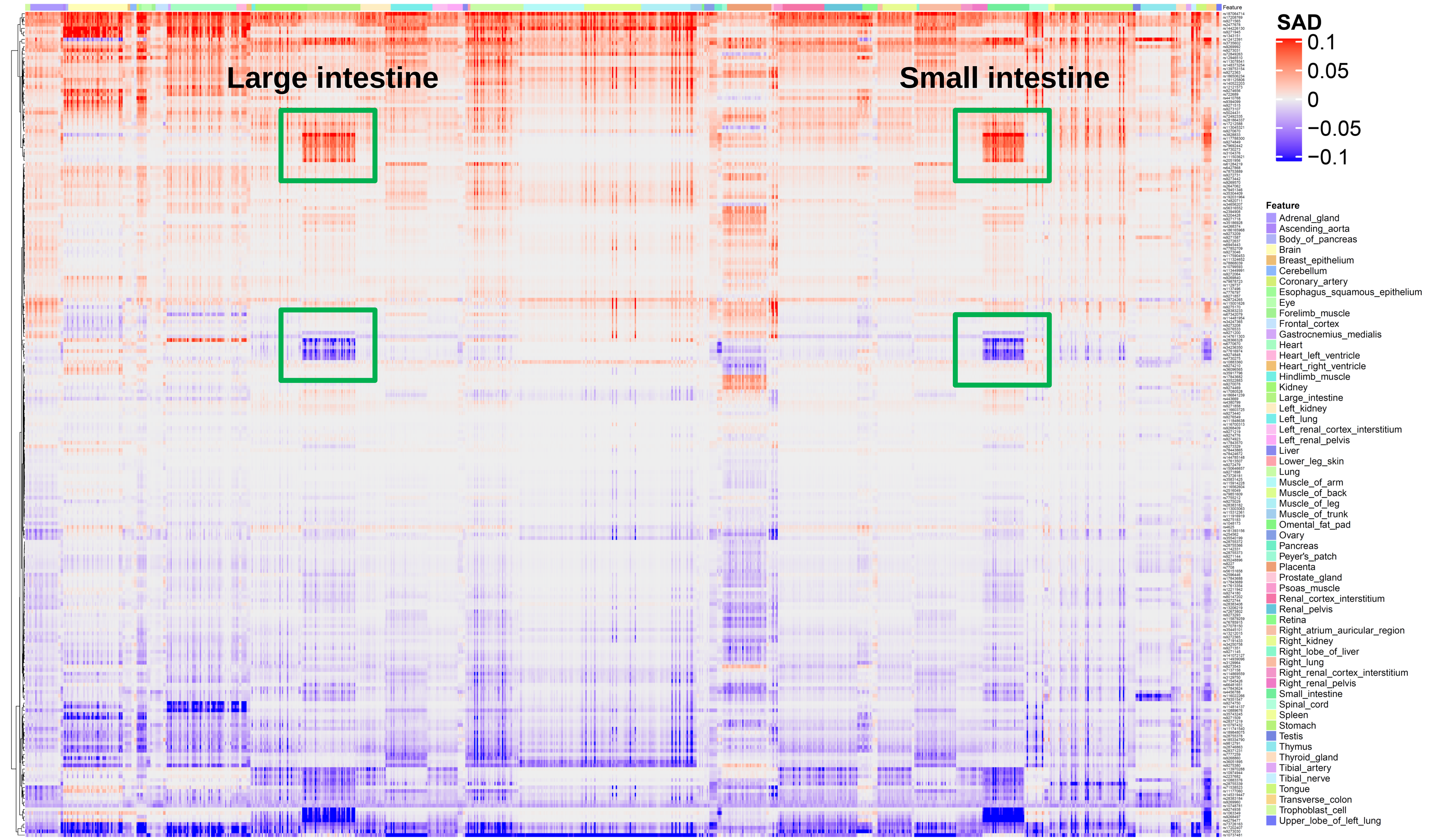
Large intestine
Small intestine
SAD
0.1
0.05
0
−0.05
−0.1
Feature
Adrenal_gland
Ascending_aorta
Body_of_pancreas
Brain
Breast_epithelium
Cerebellum
Coronary_artery
Esophagus_squamous_epithelium
Eye
Forelimb_muscle
Frontal_cortex
Gastrocnemius_medialis
Heart
Heart_left_ventricle
Heart_right_ventricle
Hindlimb_muscle
Kidney
Large_intestine
Left_kidney
Left_lung
Left_renal_cortex_interstitium
Left_renal_pelvis
Liver
Lower_leg_skin
Lung
Muscle_of_arm
Muscle_of_back
Muscle_of_leg
Muscle_of_trunk
Omental_fat_pad
Ovary
Pancreas
Peyer's_patch
Placenta
Prostate_gland
Psoas_muscle
Renal_cortex_interstitium
Renal_pelvis
Retina
Right_atrium_auricular_region
Right_kidney
Right_lobe_of_liver
Right_lung
Right_renal_cortex_interstitium
Right_renal_pelvis
Small_intestine
Spinal_cord
Spleen
Stomach
Testis
Thymus
Thyroid_gland
Tibial_artery
Tibial_nerve
Tongue
Transverse_colon
Trophoblast_cell
Upper_lobe_of_left_lung
