# Supplementary material for: Predicting regulatory variants using a dense epigenomic mapped CNN model elucidated the molecular basis of trait-tissue associations
Source: Nucleic Acids Res. 2020 Dec 9;49(1):53–66. doi: 10.1093/nar/gkaa1137 (PMC7797043; doi:10.1093/nar/gkaa1137)
Supplement: gkaa1137_Supplemental_Files [file gkaa1137_supplemental_files.zip › Supplementary Materials.pdf]

Supplementary Materials for

Predicting regulatory variants using a dense epigenomic mapped CNN model elucidated the molecular basis of trait-tissue associations

Guangsheng Pei[1], Ruifeng Hu[1], Yulin Dai[1], Astrid Marilyn Manuel[1], Zhongming Zhao[1, 2, 3, 4*], Peilin Jia[1,*]

[1]Center for Precision Health, School of Biomedical Informatics, The University of Texas Health Science Center at Houston, Houston, TX 77030, USA

[2]Human Genetics Center, School of Public Health, The University of Texas Health Science Center at Houston, Houston, TX 77030, USA

[3]MD Anderson Cancer Center UTHealth Graduate School of Biomedical Sciences, Houston, TX 77030, USA

[4]Department of Biomedical Informatics, Vanderbilt University Medical Center, Nashville, TN 37203, USA

[*] To whom correspondence should be addressed:

Zhongming Zhao, Ph.D.
Center for Precision Health
School of Biomedical Informatics
The University of Texas Health Science Center at Houston
7000 Fannin St. Suite 600, Houston, TX 77030
Phone: 713-500-3631
Email: zhongming.zhao@uth.tmc.edu

Peilin Jia, Ph.D.
Center for Precision Health
School of Biomedical Informatics
The University of Texas Health Science Center at Houston
7000 Fannin St. Suite 600, Houston, TX 77030
Phone: 713-500-3633
Email: peilin.jia@uth.tmc.edu

This PDF file includes:

Supplementary Materials and Methods

References

Captions for Supplementary Figures S1 to S7

Captions for Supplementary Tables S1 to S5

Supplementary Materials and Methods

In this Supplementary material, we provide further information regarding one example of DeepFun *in silico* saturated mutagenesis analysis, weighted gene co-expression network analysis (WGCNA) and tissue-specific enrichment analysis on BrainSpan data.

DeepFun *in silico* saturated mutagenesis pinpoints transcription factor binding motifs

To recognize informative sequence features from transcription factor (TF) binding affinity profiles, '*in silico* saturated mutagenesis' approach by scanning along all potential single-nucleotide substitutions was integrated to DeepFun model to assess the effect of mutating every base, and what sequence features are informative for a specific chromatin profiles effect prediction (1,2). For better representing our results, we selected one autism spectrum disorder (ASD) associated SNP, rs11685464 (p-value = 6×10^{-4}) (3) as an example, which demonstrated the highest SAD value on transcription factor FOS binding affinity profile on MOF-7 cell line.

As shown in **Supplementary Figure S7**, two heat maps are displayed to show the saturated mutagenesis effects (SAD values) of rs11685464 from upstream to downstream 50 base pair on reference and alternative alleles, followed by an estimation of each individual nucleotide contribution to sequence's binding activity. In addition, the predicted binding activity statistics in G allele sequence is highly consistent with relevance FOS binding motif TGACTCAT, any mutations around motif and flanking regions would result in predicted biding activity

decrease (**Supplementary Figure S7A)**. On the contrary, the predicted binding activity statistics in C allele sequence shows that, only mutating from C to G would result in predicted binding activity increase (**Supplementary Figure S7B**), while any mutations around rs11685464 could not bring great change in binding activity. Therefore, *in silico* saturated mutagenesis is not only provide the opportunity to extensively investigate the impact of variant to transcription factor binding affinity in a cell-specific fashion, but also an effective approach for dissecting potential sequence feature of binding motif (1).

Different TFs in human genome involved in different biological process, e.g., *MYBL2* involved in cell proliferation, cell survival and differentiation during tumorigenesis (4); *GATA* involved in cardiac development (5); *MITF* involved in melanoma driver (6). By integrating 4,796 TF binding profiles from 305 TF over 66 tissue or cell types, DeepFun would great facilitate the high-throughput evaluation and prioritization of non-coding variants to different TFs across different cell types.

BrainSpan transcriptomic data collection

The transcriptomic data of developing human brain was downloaded from the Allen Institute BrainSpan Atlas. The raw data comprised 524 transcriptomes from 42 individuals ranging in age between 8 postconceptional weeks (pcw) to 40 years across 26 brain regions. The overall missing data rate was ~52% (568 out of 42 × 26=1092 transcriptomes), with 16/26 regions sampled for at least 20 individuals and 35/42 individuals sampled for at least 5 regions. After removing individuals with high missing data rates (i.e., ≥15%), we obtained a working data set with 35 individuals for 16 regions. Hereafter, we refer one sample as a transcriptome that was measured at a particular developmental stage in a particular region. For temporal information, we group all samples into 6 stages, corresponding to prenatal-early (before 12 pcw), prenatal-middle (from 13 to 21 pcw), prenatal-late (after 24 pcw), postnatal-early (4 months to 4 years), postnatal-middle (8 to 13 years), postnatal-late stages (18 to 40 years).

Weighted gene co-expression network analysis (WGCNA)

All gene expression values were firstly converted into z-score, and the weighted gene co-expression network analysis (WGCNA, v1.67) approach (7) was used to establish a co-expression network from the clean BrainSpan RNAseq profiles. The co-expression network was created by following the standard procedure of WGCNA. Briefly, weighted correlation matrices were transformed into matrices of connection strengths follows a power law distribution (scale free topology). These connection strengths were then used to calculate topological overlap matrix (TOM) (7), which is computed as:

$$\mathrm{TOM}_{ij} = \frac{(l_{ij} + a_{ij})}{\min(k_i, k_j) + 1 - a_{ij}}$$

where l_{ij} is defined as the dot product on row *i* and column *j* in adjacency matrix [*a*] and k_i (the connectivity) is the summation of row *i* in adjacency matrix [*a*] (8). Hierarchical clustering based on TOM was used to group genes with highly similar co-expression relationships into modules. Gene dendrogram was obtained by average linkage hierarchical clustering, while the color row underneath the dendrogram showed the module assignment determined by the Dynamic Tree Cut method (9). The network for each module was generated with the minimum spanning tree with a dissimilarity matrix from WGCNA. The modules with $r > 0.5$ and a p-value < $1e^{-5}$ were extracted as traits associate modules. For gene within each module, *deTS* package (10) were applied to explore their tissue specificity.

References

1. Kelley, D.R., Snoek, J. and Rinn, J.L. (2016) Basset: learning the regulatory code of the accessible genome with deep convolutional neural networks. *Genome Res*, 26, 990-999.

2. Zhou, J. and Troyanskaya, O.G. (2015) Predicting effects of noncoding variants with deep learning-based sequence model. *Nat Methods*, 12, 931-934.

3. Demontis, D., Walters, R.K., Martin, J., Mattheisen, M., Als, T.D., Agerbo, E., Baldursson, G., Belliveau, R., Bybjerg-Grauholm, J., Baekvad-Hansen, M. *et al.* (2019) Discovery of the first genome-wide significant risk loci for attention deficit/hyperactivity disorder. *Nat Genet*, 51, 63-75.

4. Musa, J., Aynaud, M.M., Mirabeau, O., Delattre, O. and Grunewald, T.G. (2017) MYBL2 (B-Myb): a central regulator of cell proliferation, cell survival and differentiation involved in tumorigenesis. *Cell Death Dis*, 8, e2895.

5. Pikkarainen, S., Tokola, H., Kerkela, R. and Ruskoaho, H. (2004) GATA transcription factors in the developing and adult heart. *Cardiovasc Res*, 63, 196-207.

6. Hartman, M.L. and Czyz, M. (2015) MITF in melanoma: mechanisms behind its expression and activity. *Cellular and molecular life sciences*, 72, 1249-1260.

7. Langfelder, P. and Horvath, S. (2008) WGCNA: an R package for weighted correlation network analysis. *BMC Bioinformatics*, 9, 1471-2105.

8. Gibbs, D.L., Baratt, A., Baric, R.S., Kawaoka, Y., Smith, R.D., Orwoll, E.S., Katze, M.G. and McWeeney, S.K. (2013) Protein co-expression network analysis (ProCoNA). *J Clin Bioinforma*, 3, 2043-9113.

9. Langfelder, P., Zhang, B. and Horvath, S. (2008) Defining clusters from a hierarchical cluster tree: the Dynamic Tree Cut package for R. *Bioinformatics*, 24, 719-720.

10. Pei, G., Dai, Y., Zhao, Z. and Jia, P. (2019) deTS: tissue-specific enrichment analysis to decode tissue specificity. *Bioinformatics*, 35, 3842-3845.

Captions for Supplementary Figures

Supplementary Figure S1. Initial convolution filter of model A with captured functional motifs from CIS-BP database. From top to bottom, three motifs are: initial convolution filter captured transcription factor binding motif from CIS-BP database; initial convolution filter weight matrix; reverse complement of initial convolution filter weight matrix.

Supplementary Figure S2. Initial convolution filter of model B with captured functional motifs from CIS-BP database. From top to bottom, three motifs are: initial convolution filter captured transcription factor binding motif from CIS-BP database; initial convolution filter weight matrix; reverse complement of initial convolution filter weight matrix.

Supplementary Figure S3. Top 15 chromatin features for all pathogenic variants in ClinVar database with the highest SAD values.

Supplementary Figure S4. The SAD heat map visualization of ulcerative colitis associated represented SNPs (max SAD > 0.1) functional in all tissues DNase-seq chromatin feature. *X*-axis and *Y*-axis are corresponding to ulcerative colitis associated SNPs and DNase-seq profiles over different tissues.

Supplementary Figure S5. Weighted gene co-expression network analysis and tissue-specific enrichment analysis on BrainSpan data. (A) Correlation between identified modules and spatiotemporal factor indicated by WGCNA. (B) Tissue-specific enrichment analysis of 29 modules from WGCNA.

Supplementary Figure S6. Tissue specific enrichment analysis of traits non-functional loci associated genes. The heat map only shows the significant trait-tissue associations by *p*-value < 0.05, the shared gene count between trait regulatory loci associated genes and tissue-specific expressed genes were labeled on the figure.

Supplementary Figure S7. *In silico* saturated mutagenesis of autism spectrum disorder associated SNP rs11685464 on FOS binding affinity profile of MOF-7 cell line. (A) Reference allele. (B) Alternative allele. The x-axis and y-axis describes variant's upstream 50 to downstream 50 nucleobase, allele replacement by A, C, G or T, respectively. Heat map in red color denotes SAD value > 0, indicating the mutation would increase binding activity; while blue color denotes SAD value < 0, indicating the mutation would decrease binding activity.

Captions for Supplementary Tables

Supplementary Table S1. Full chromatin features summary and performance evaluation in DeepFun model.

Supplementary Table S2. Statistics of epigenomic active sites across 23 human chromosomes.

Supplementary Table S3. DeepFun model initial convolution filters captured functional motifs from CIS-BP database statistics.

Supplementary Table S4. DeepFun prediction summary for 11 pathogenic varaints located in intergenic region from ClinVar database.

Supplementary Table S5. GWAS summary and regulatory loci associated genes statistics for 51 human complex traits.
