# Supplementary figures and images for: Predicting regulatory variants using a dense epigenomic mapped CNN model elucidated the molecular basis of trait-tissue associations
Source: Nucleic Acids Res. 2020 Dec 9;49(1):53–66. doi: 10.1093/nar/gkaa1137 (PMC7797043; doi:10.1093/nar/gkaa1137)

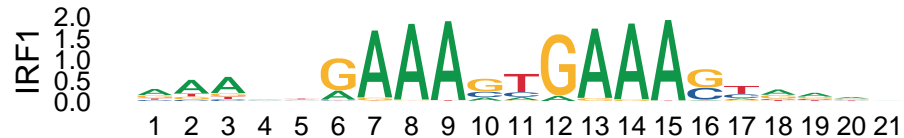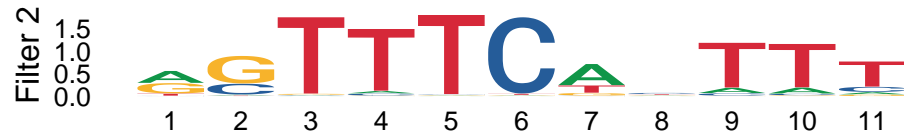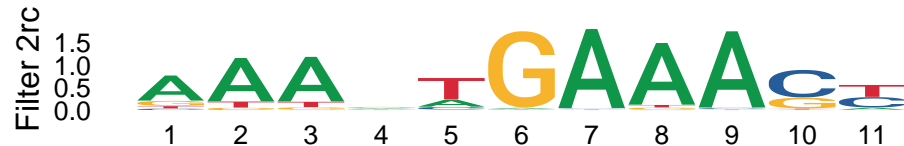

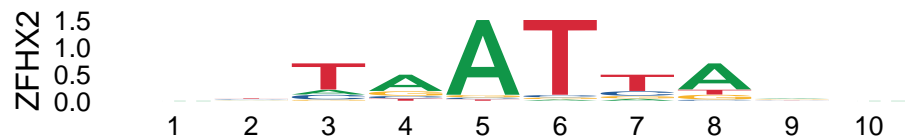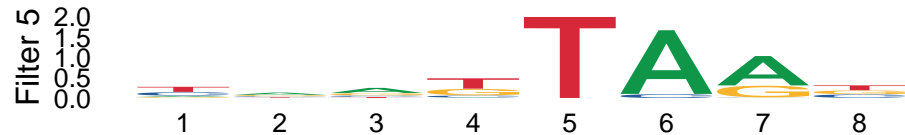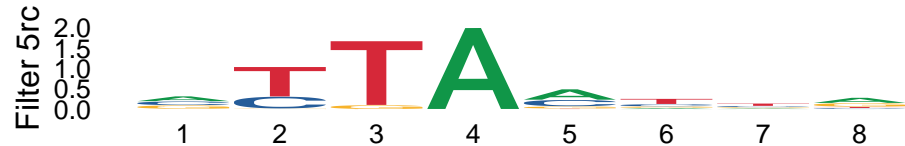

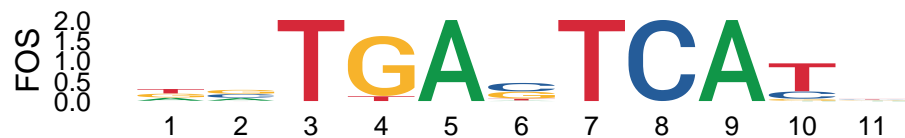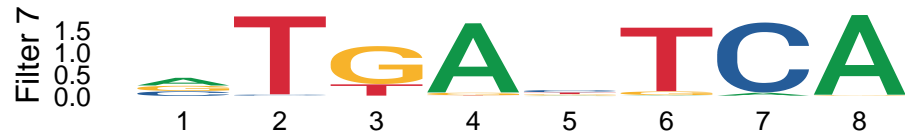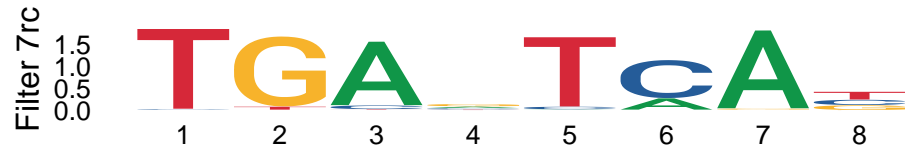

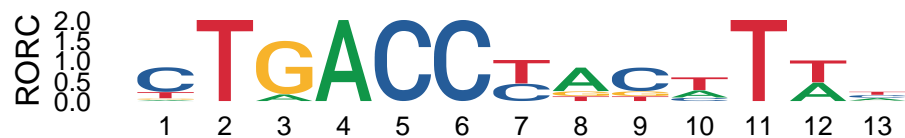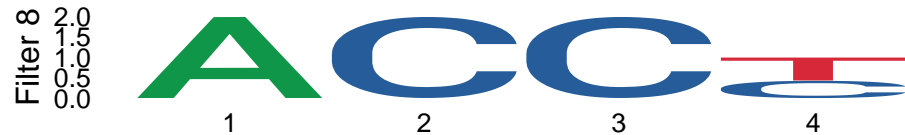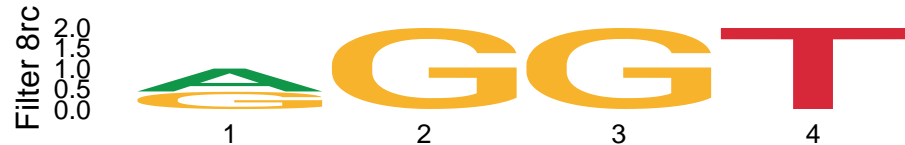

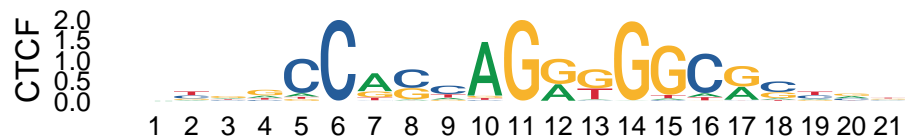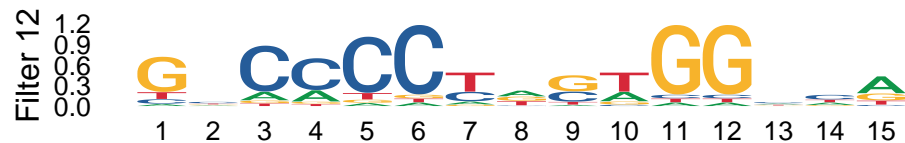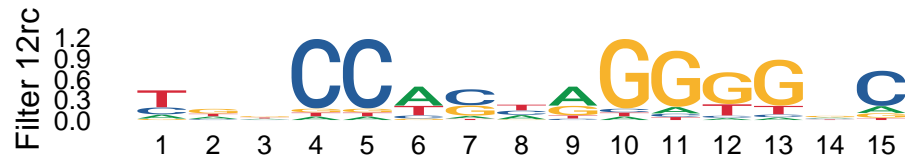

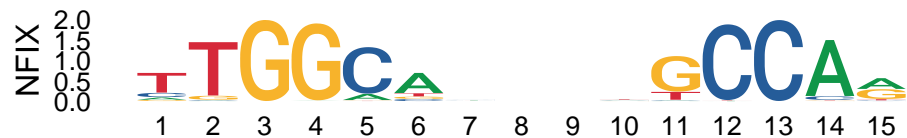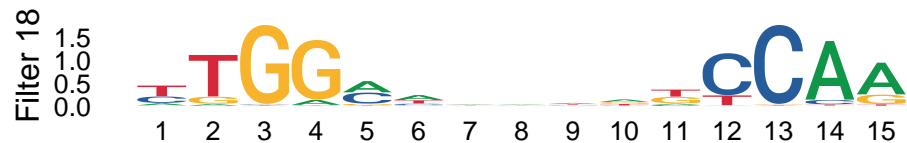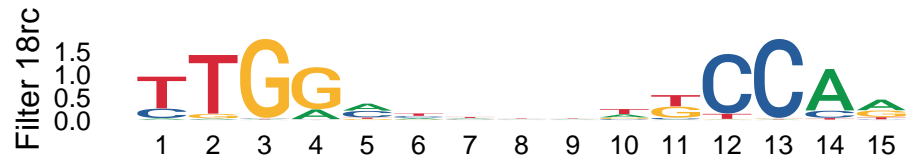

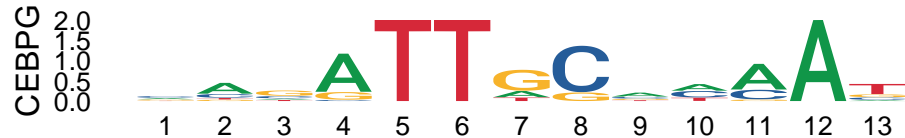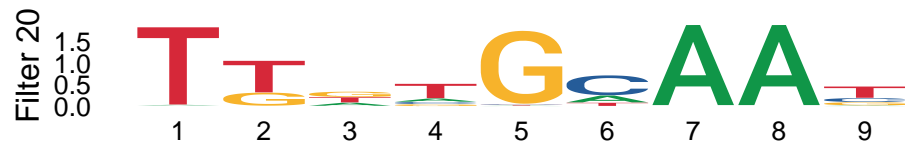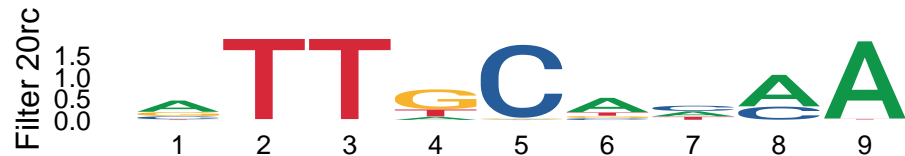

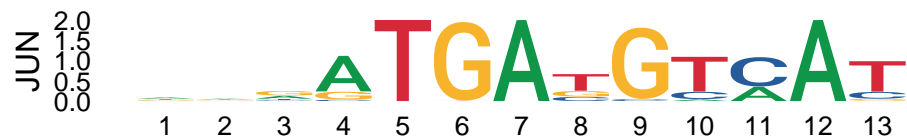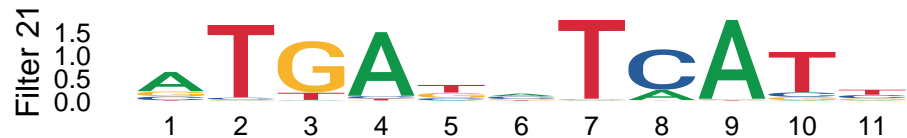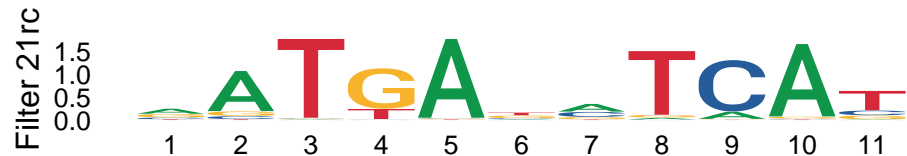

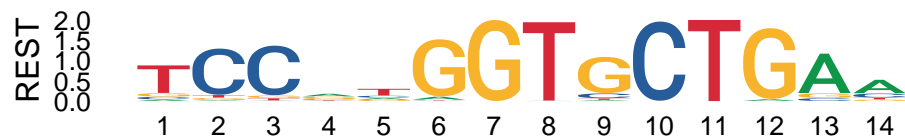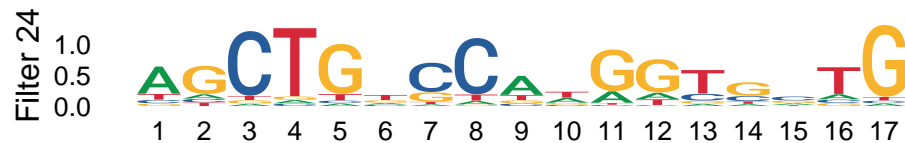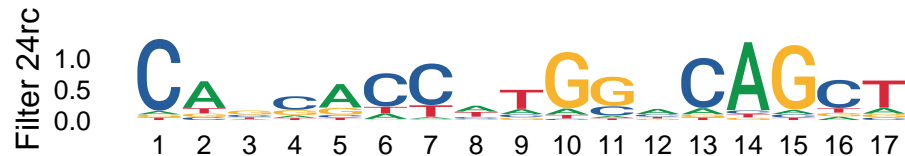

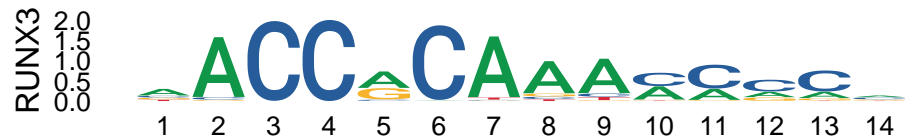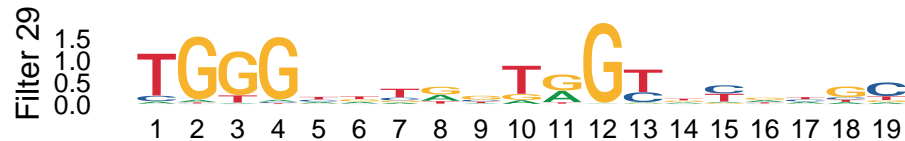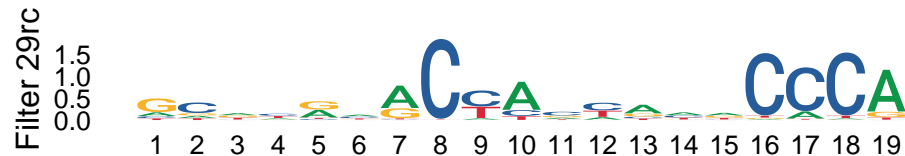

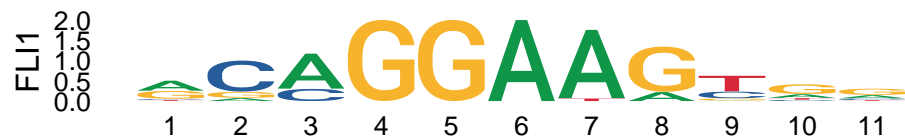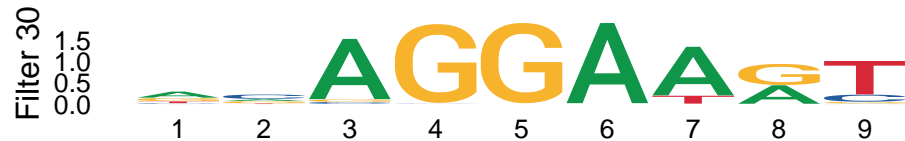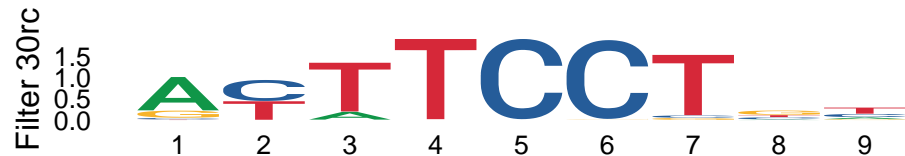

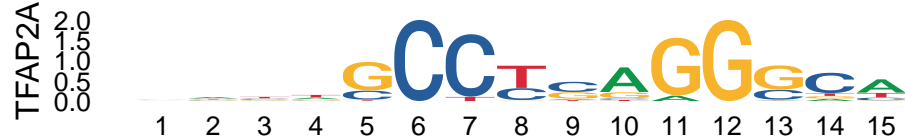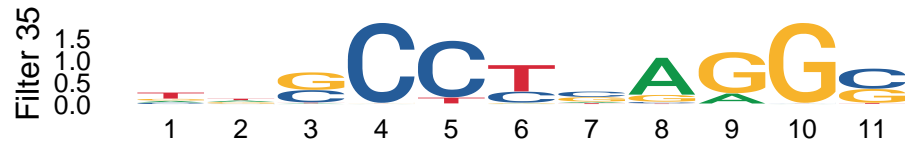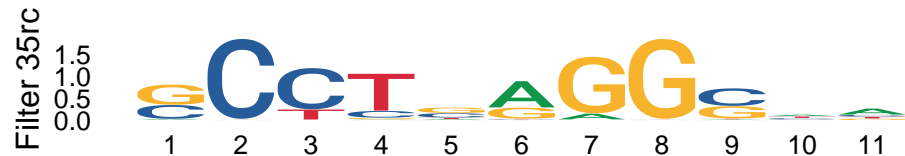

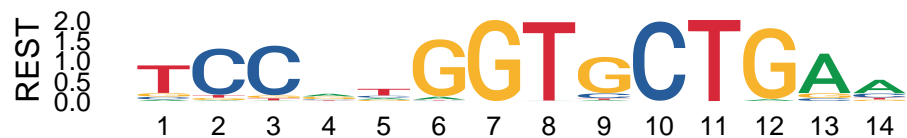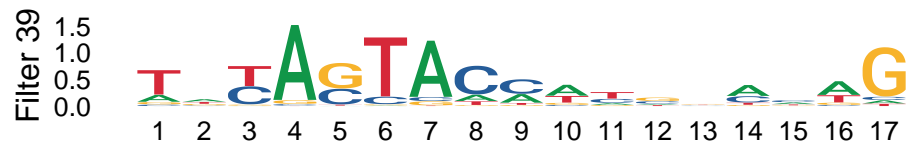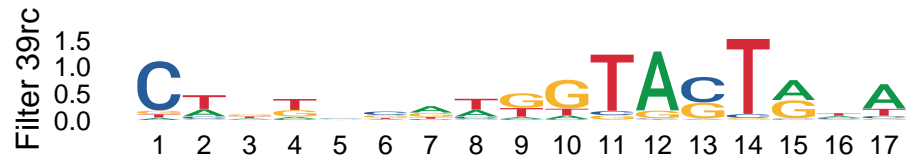

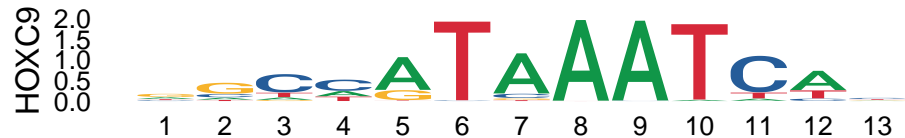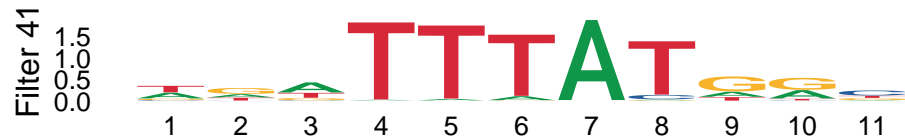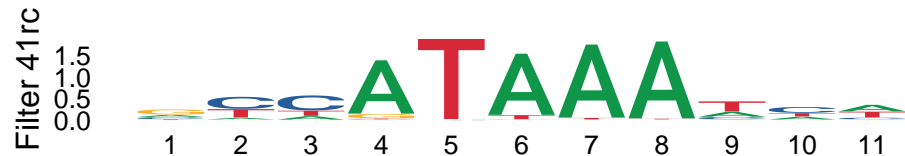

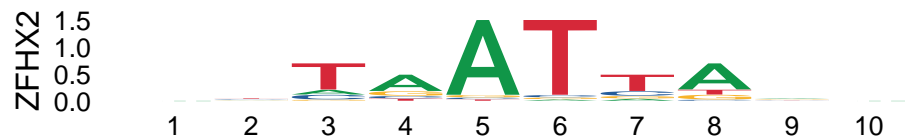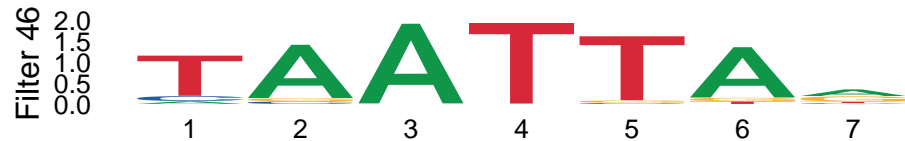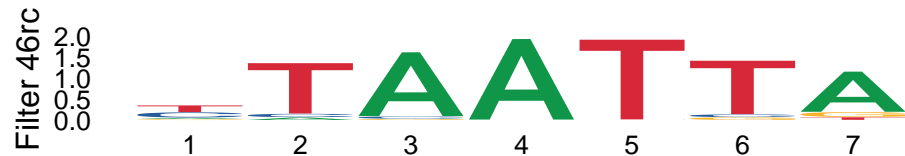

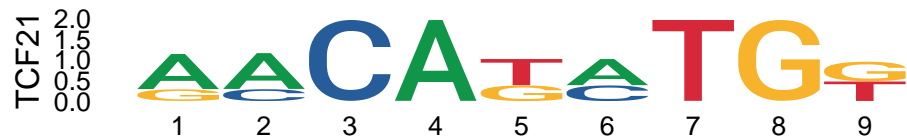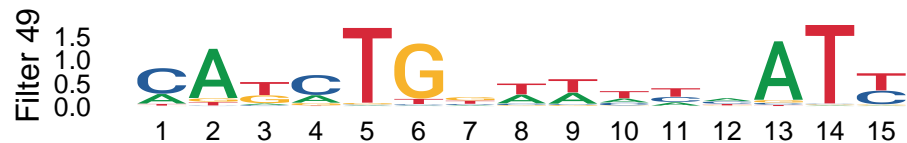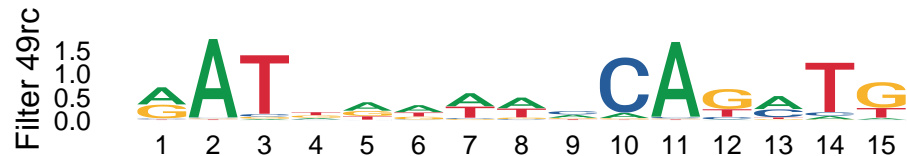



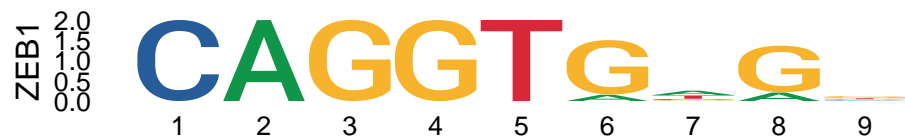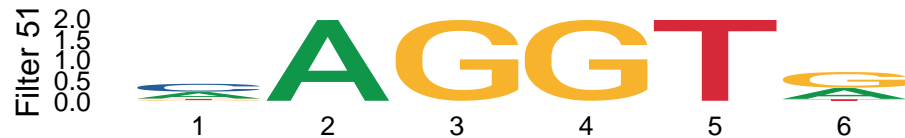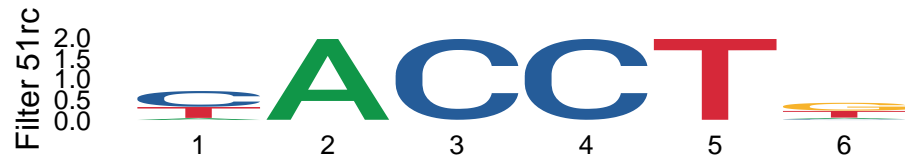

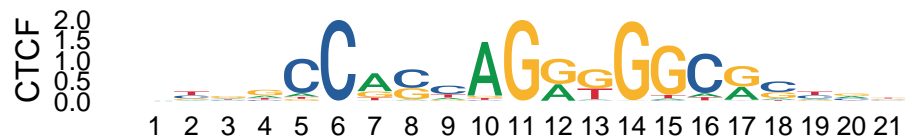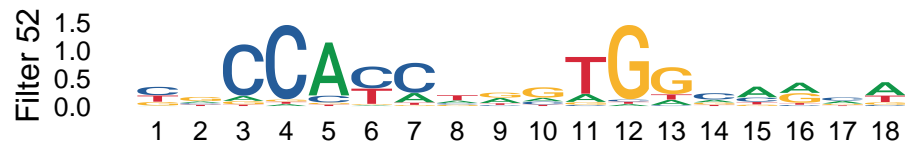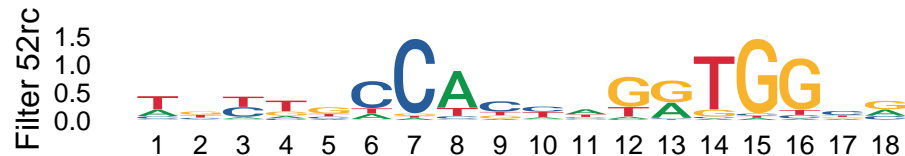

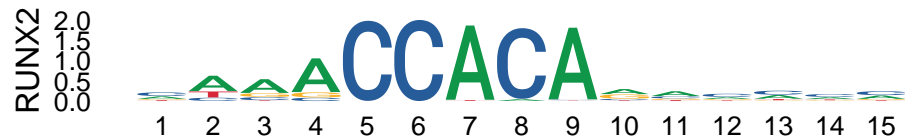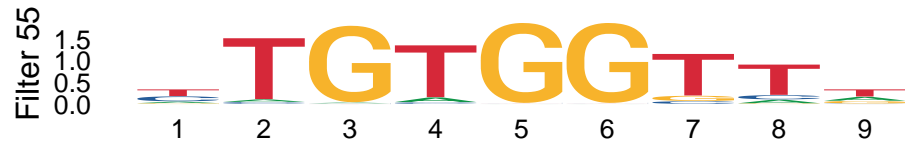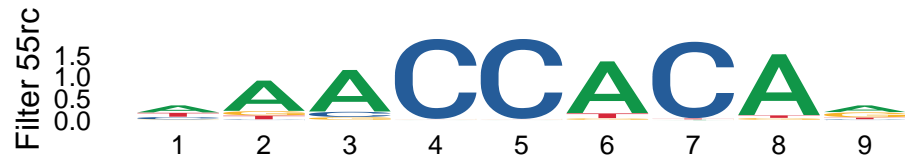

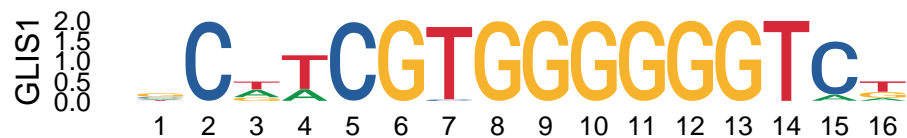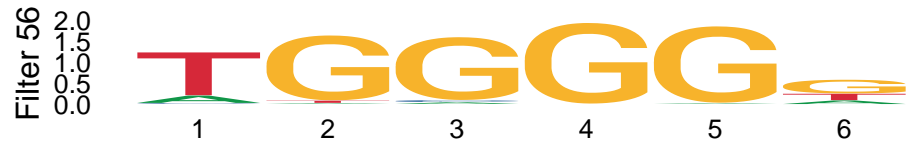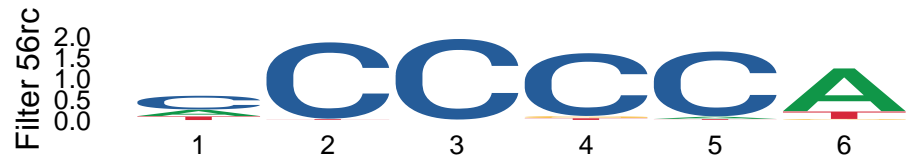

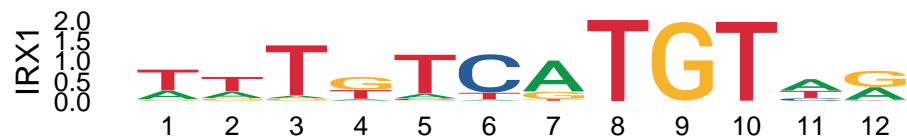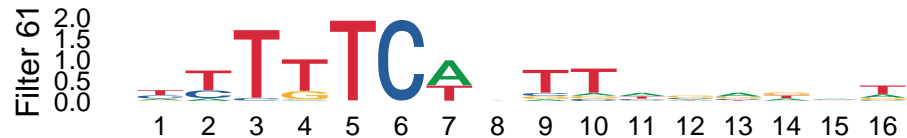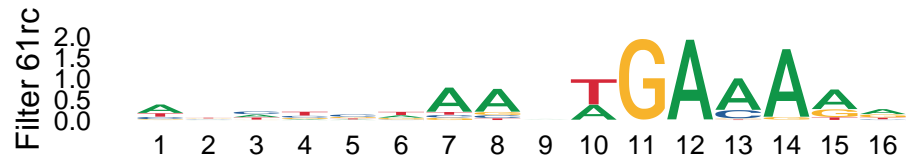

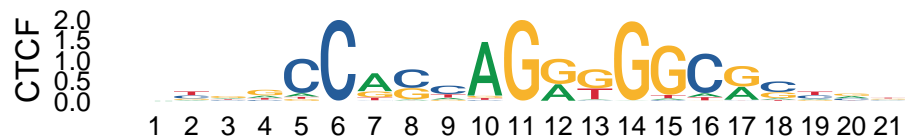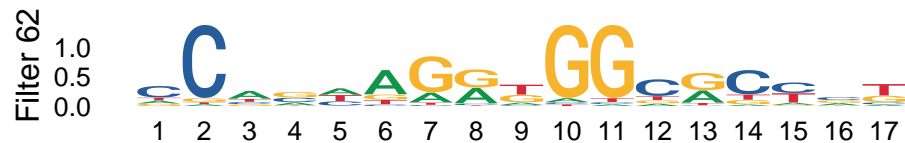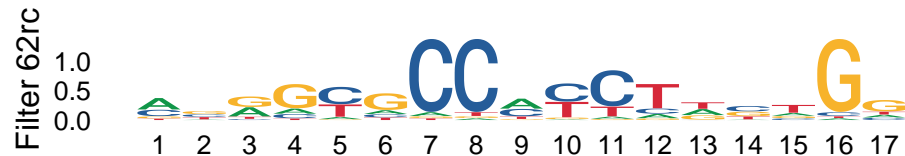

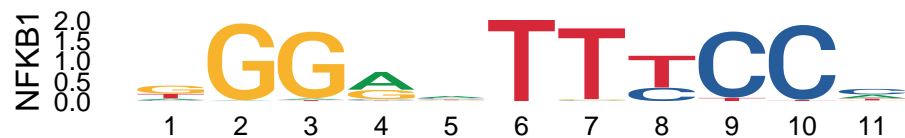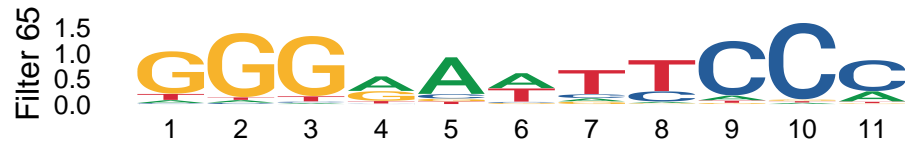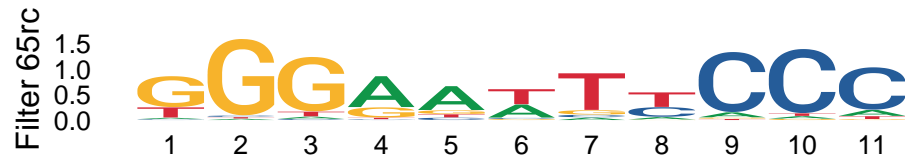



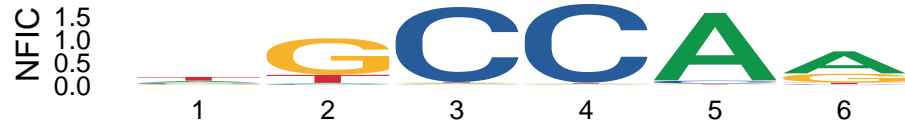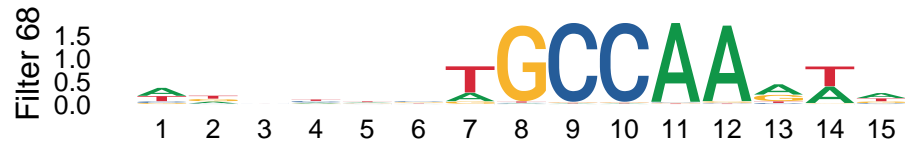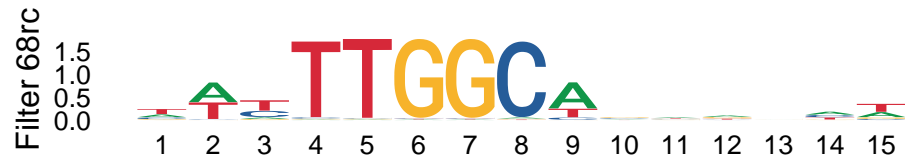

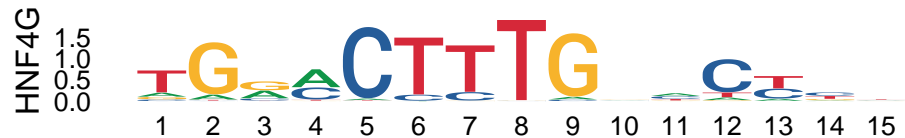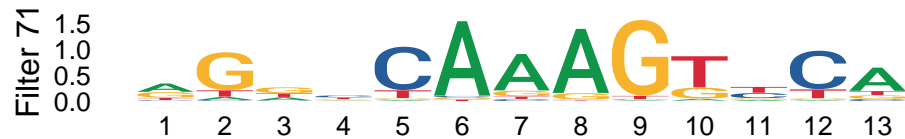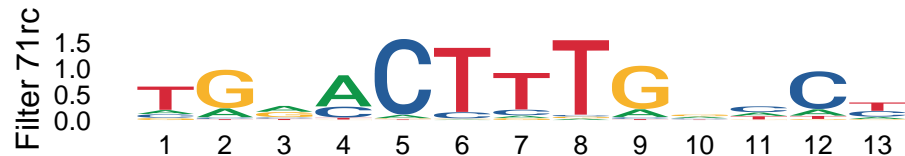

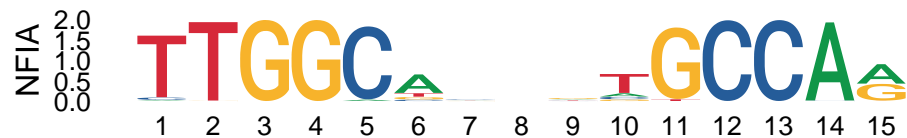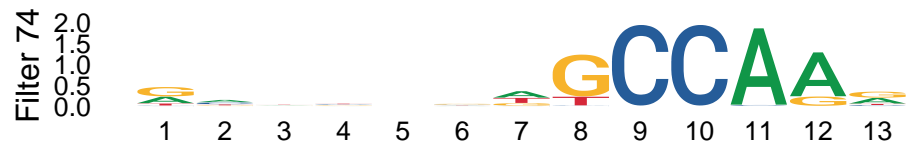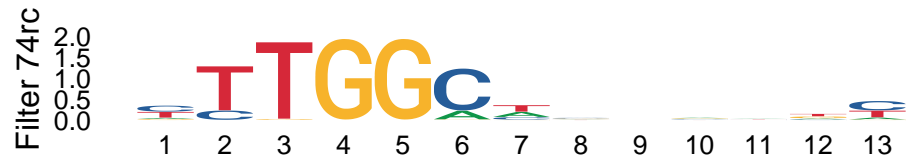

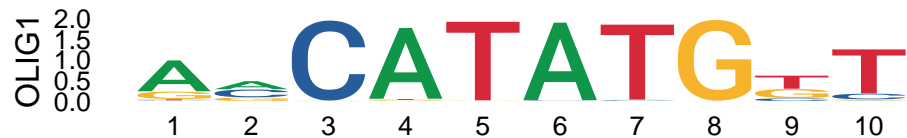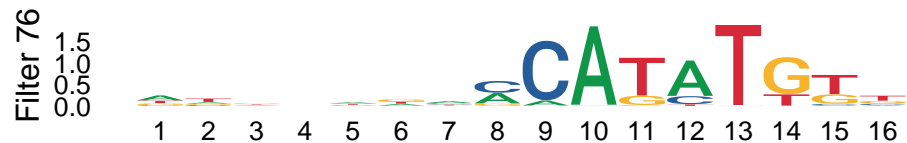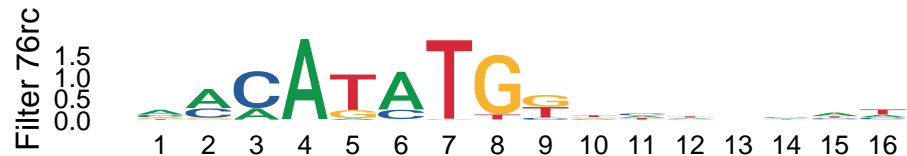

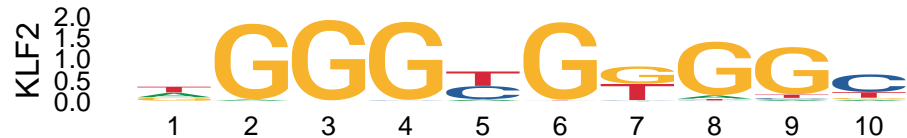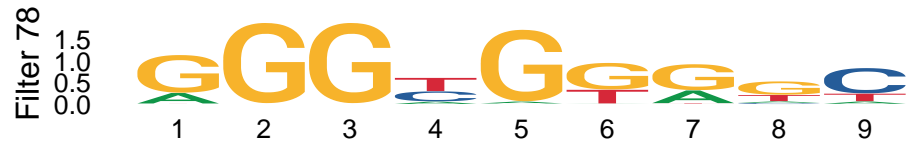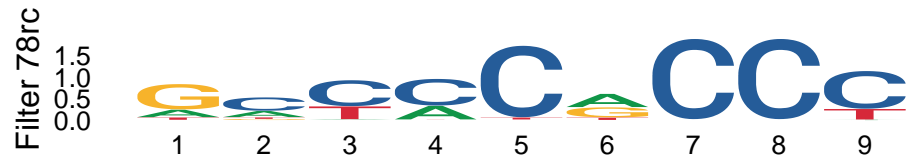

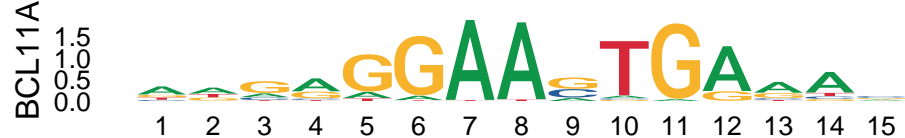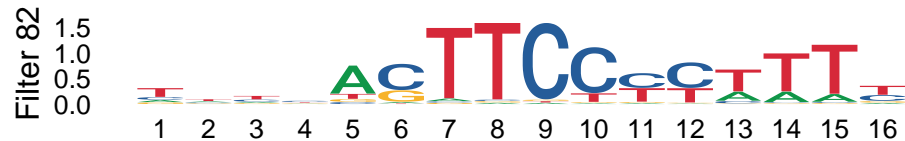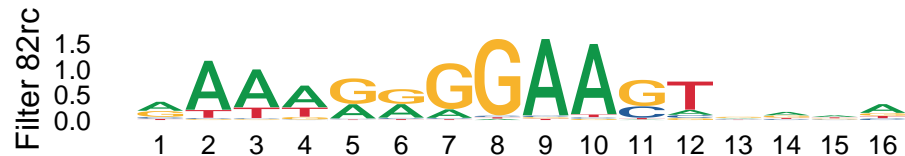

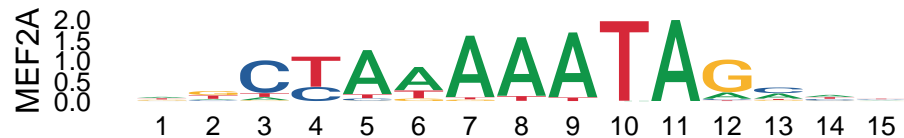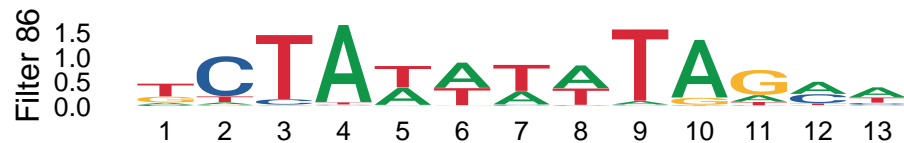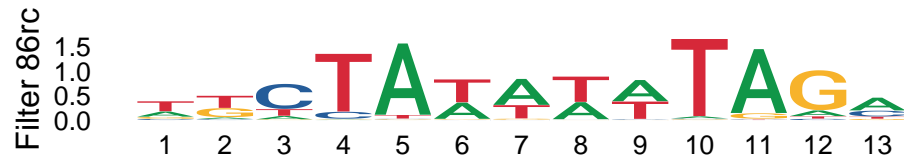

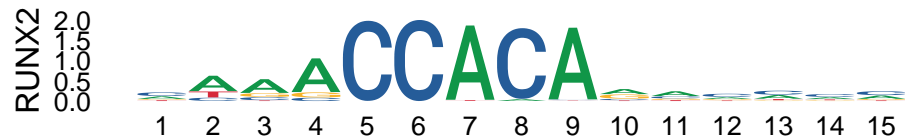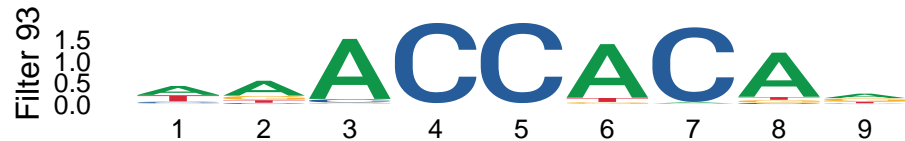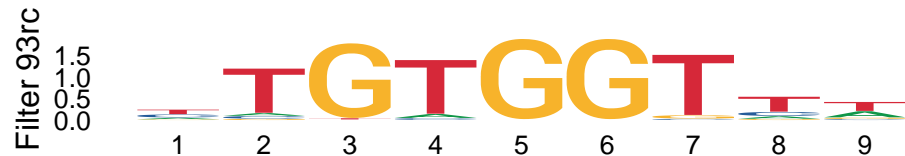

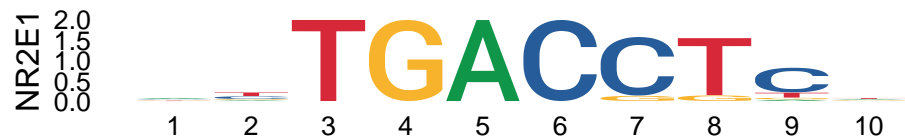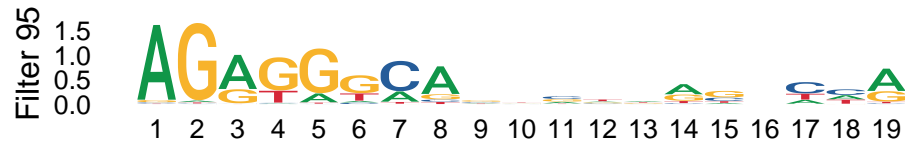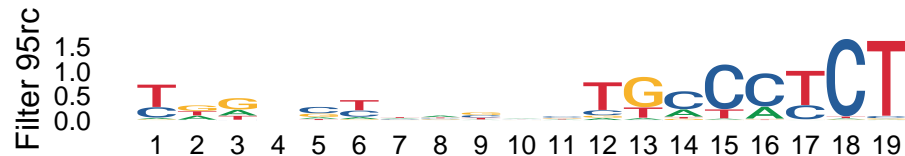

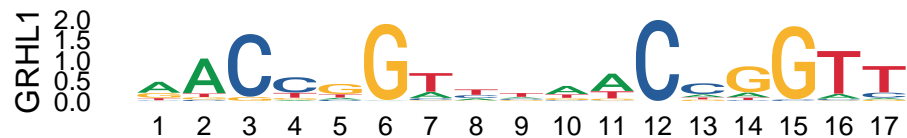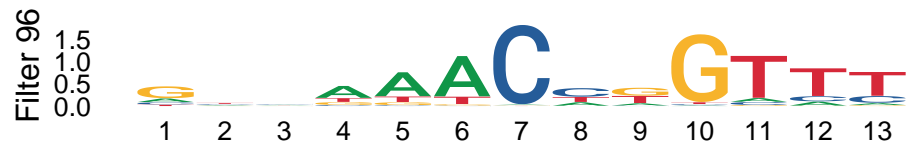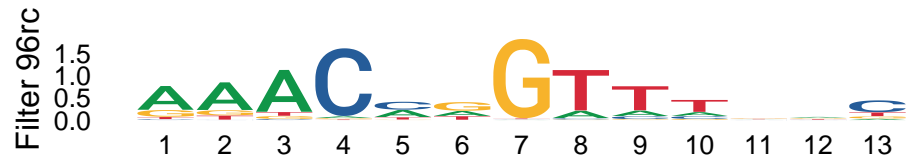

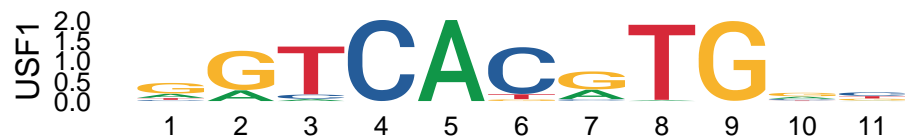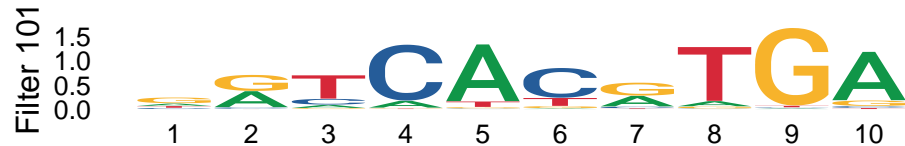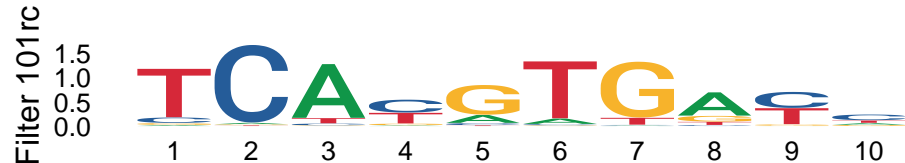

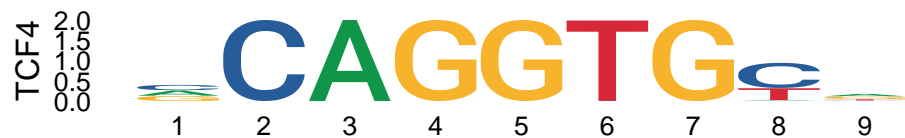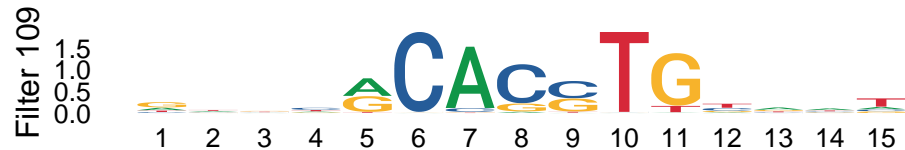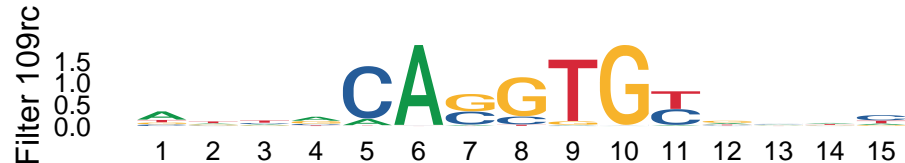

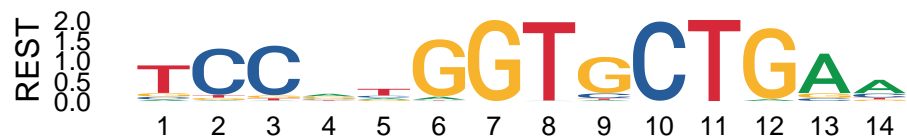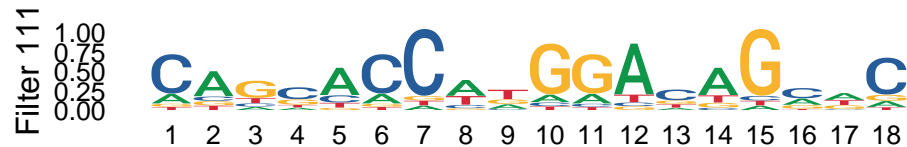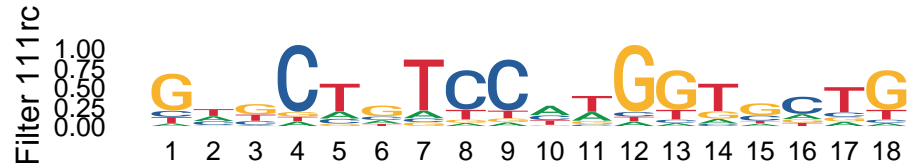

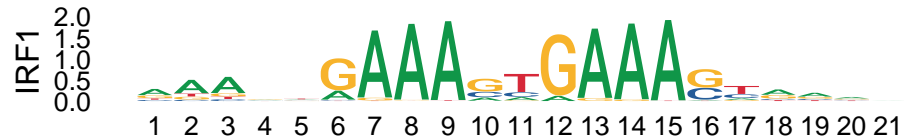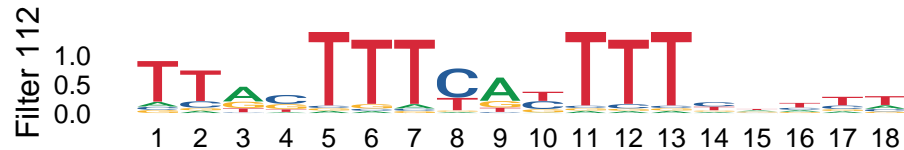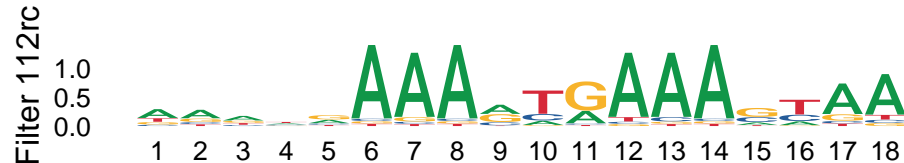

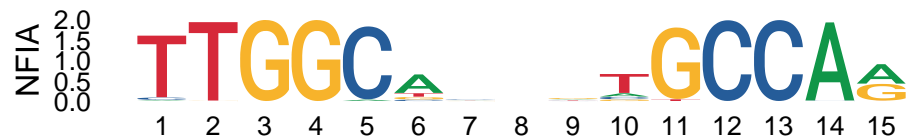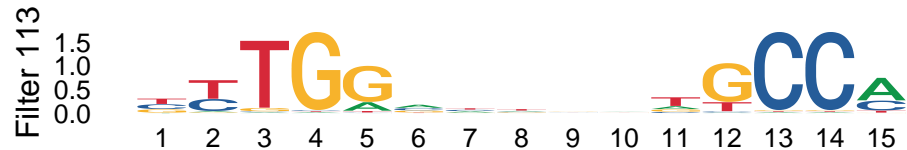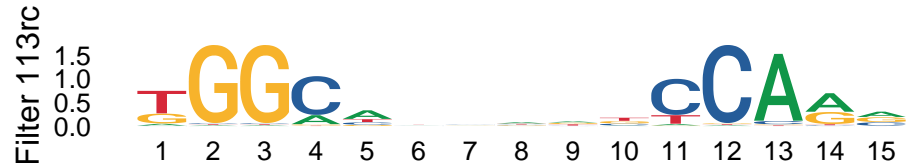

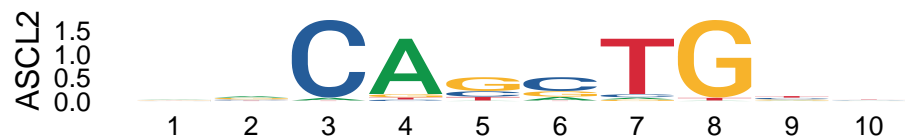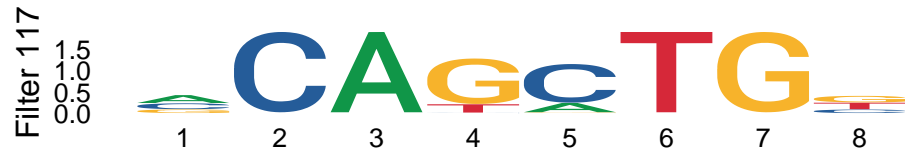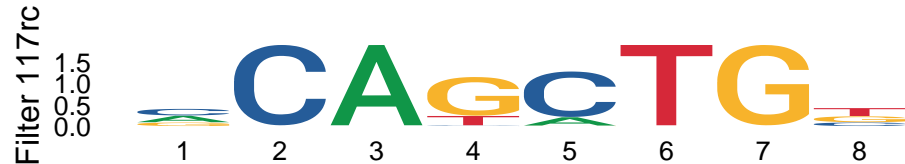

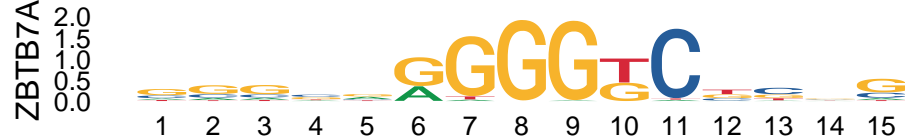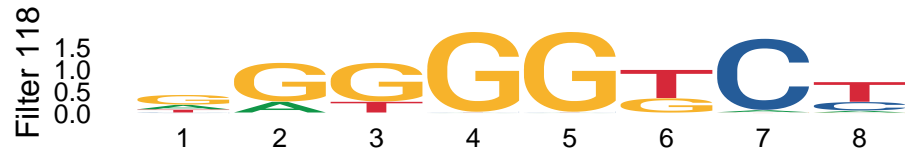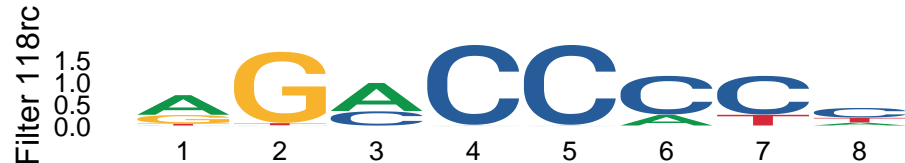

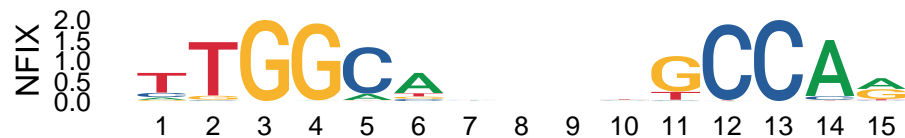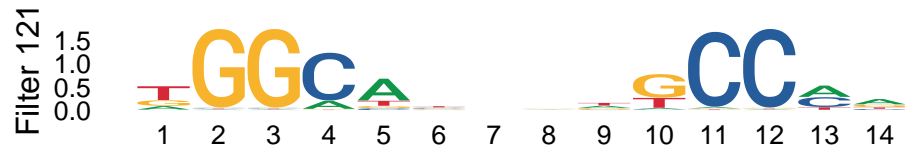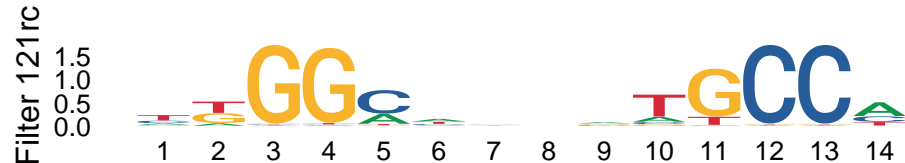

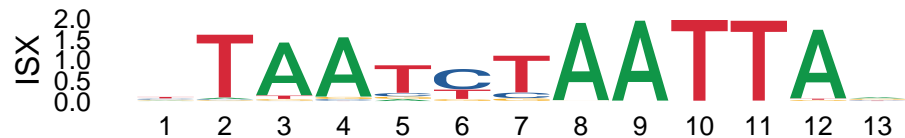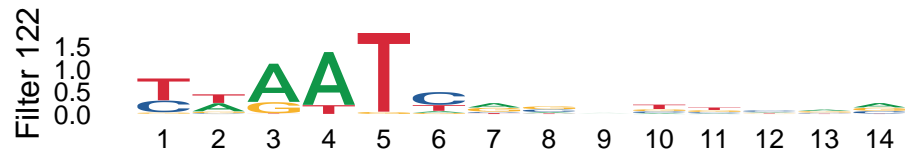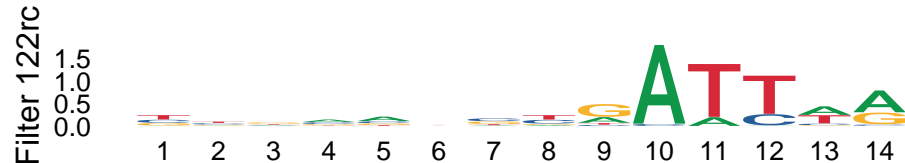

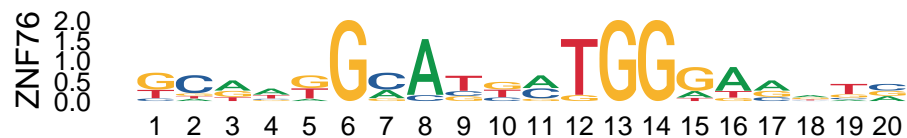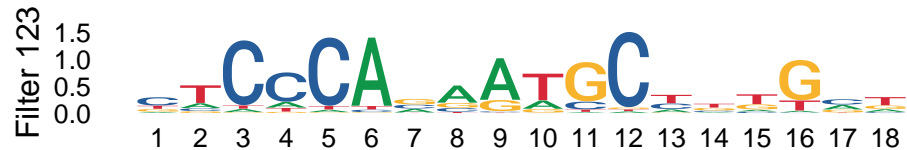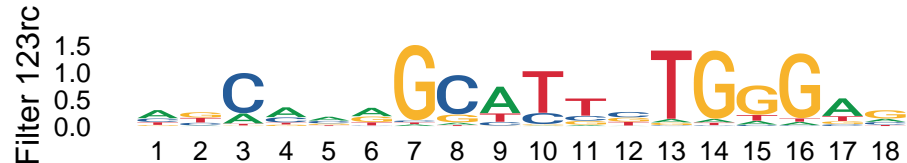

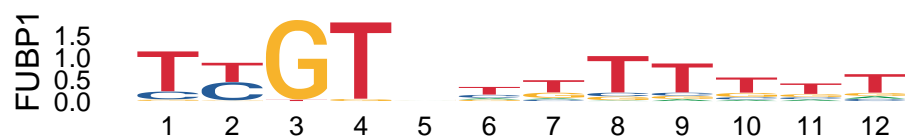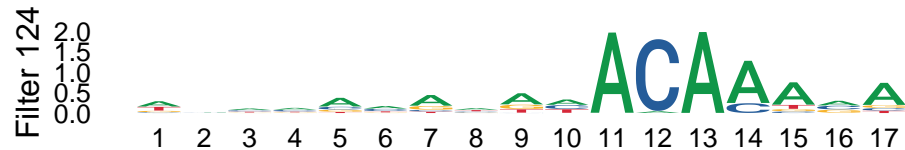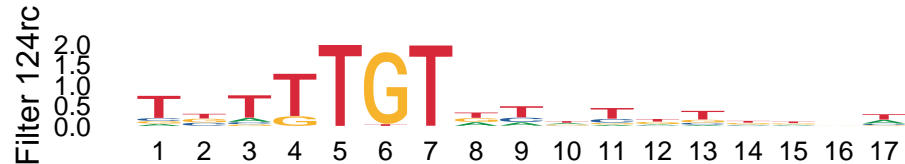



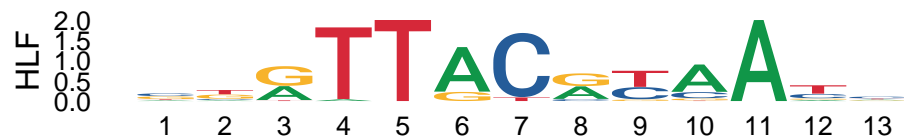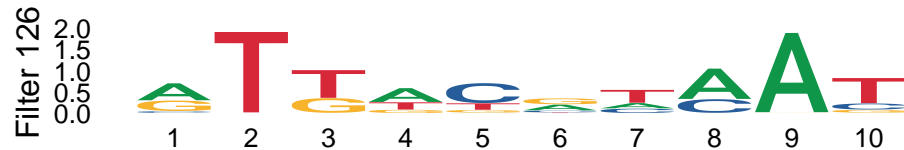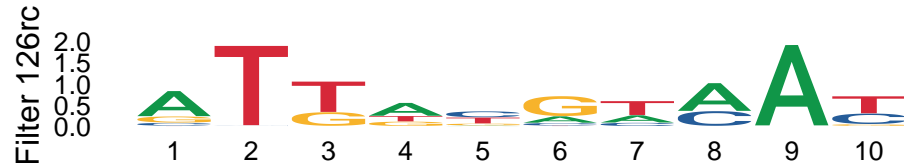

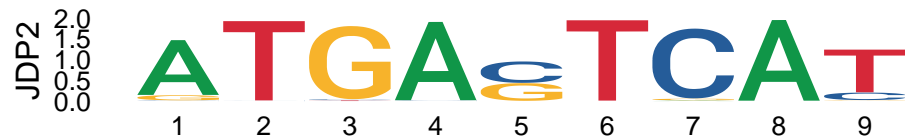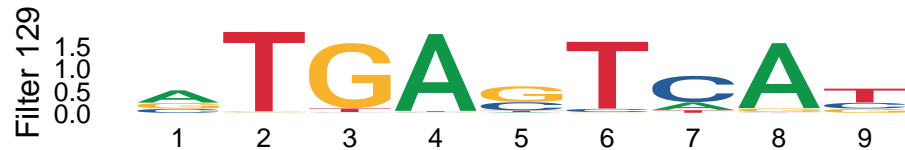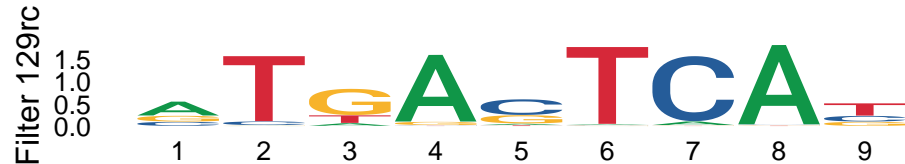

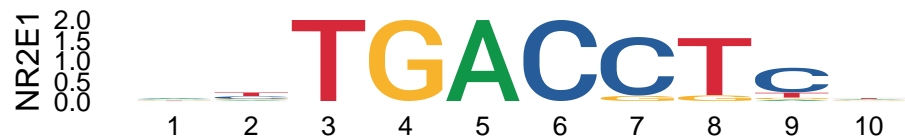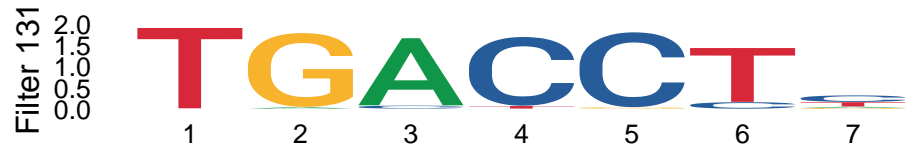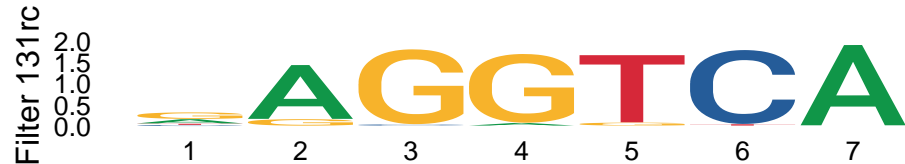

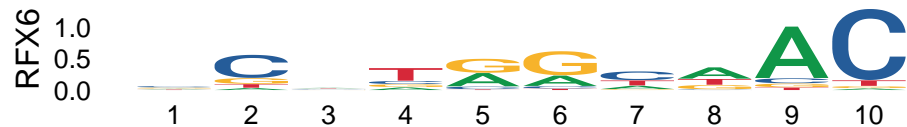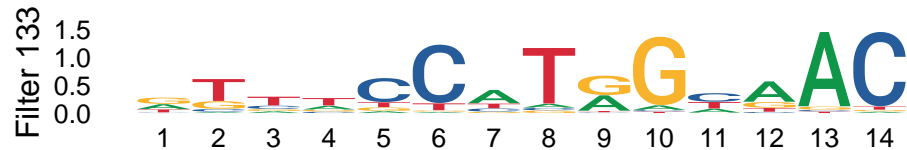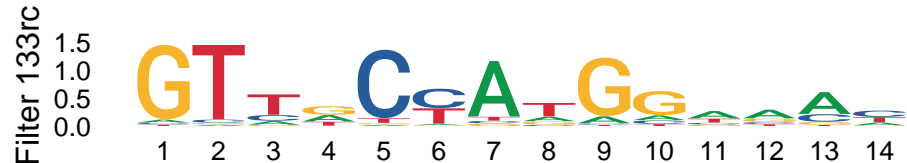

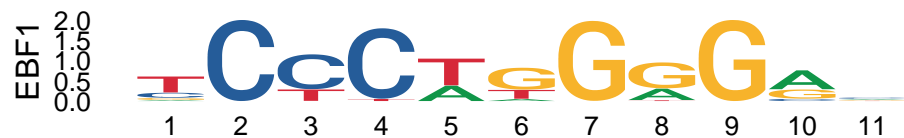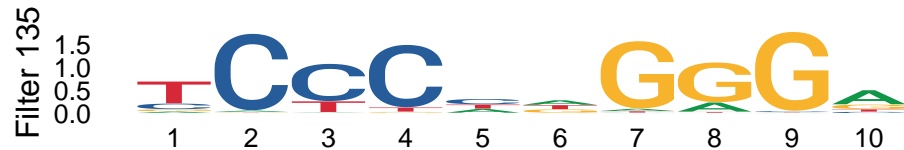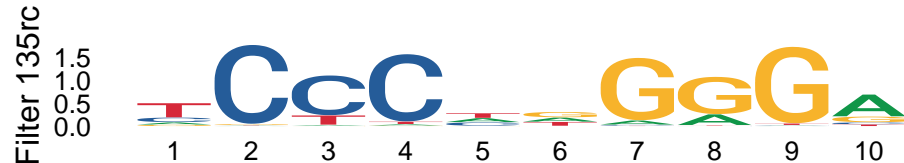

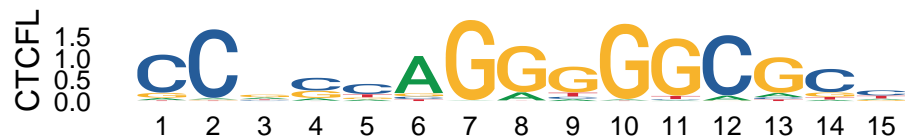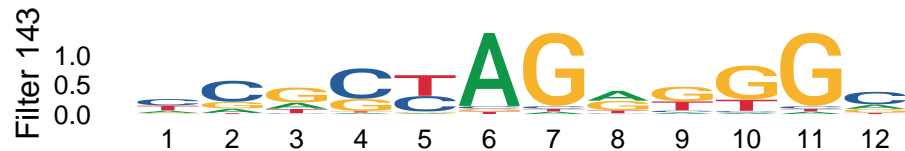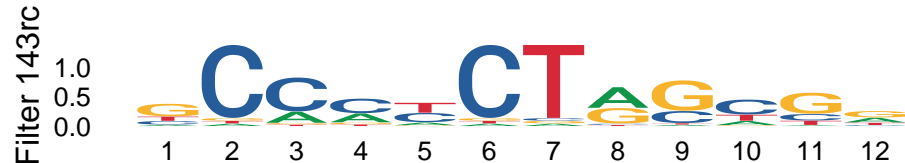

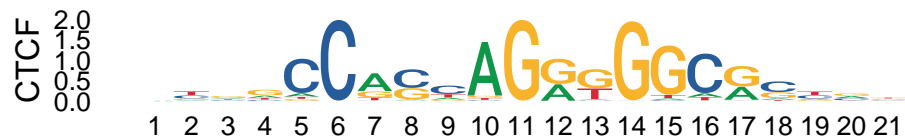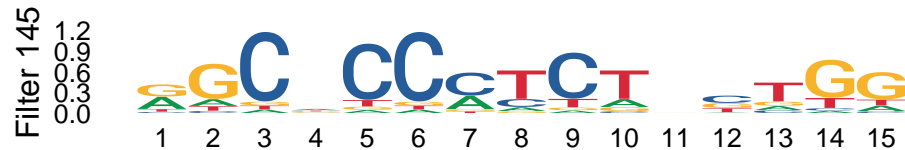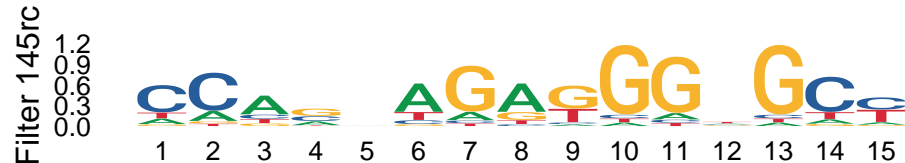

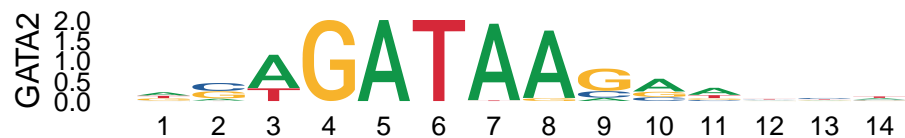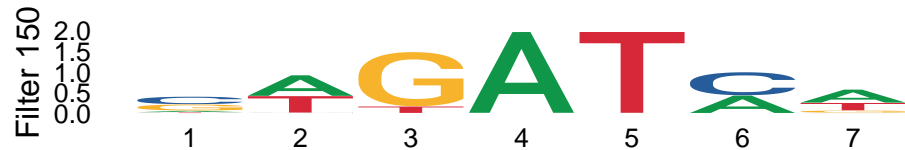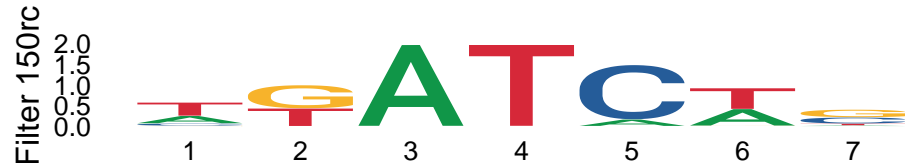

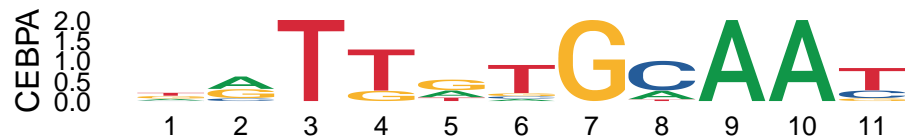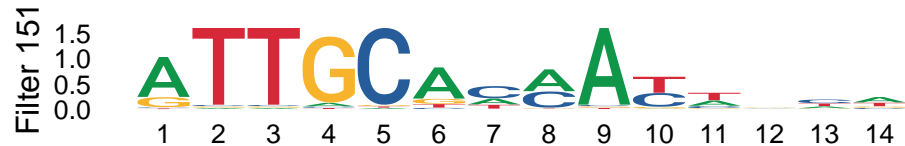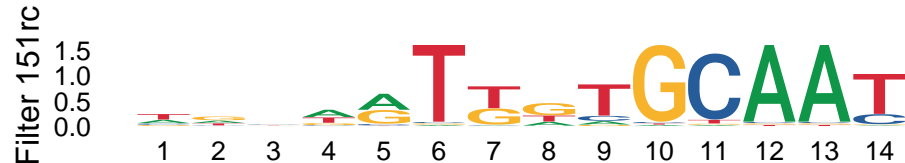

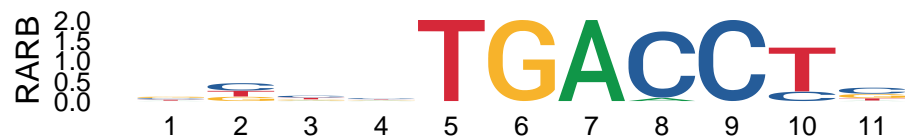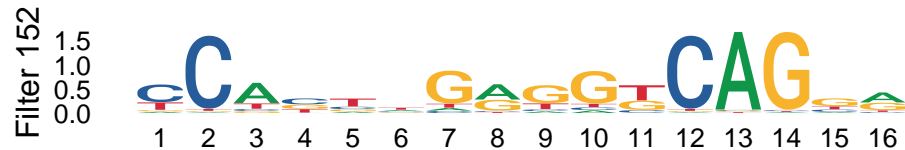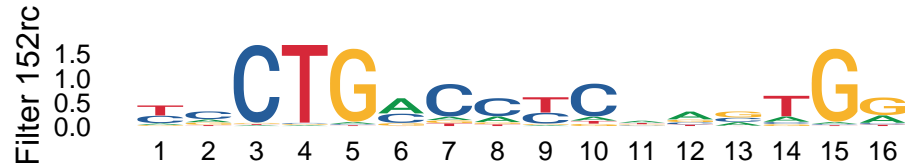

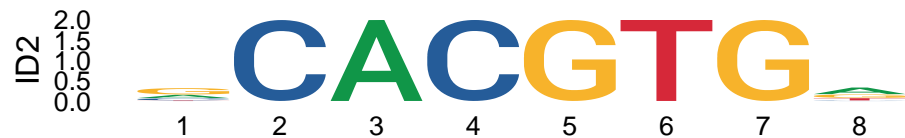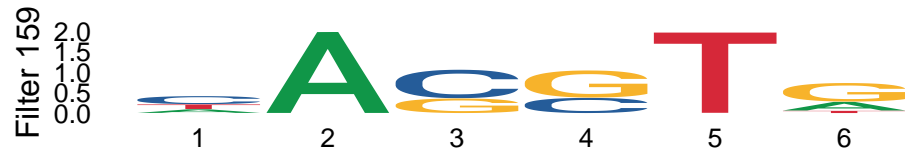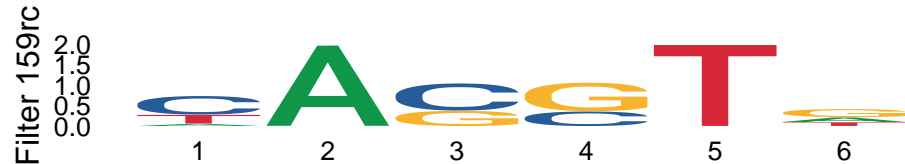

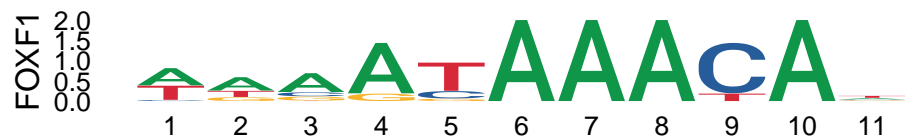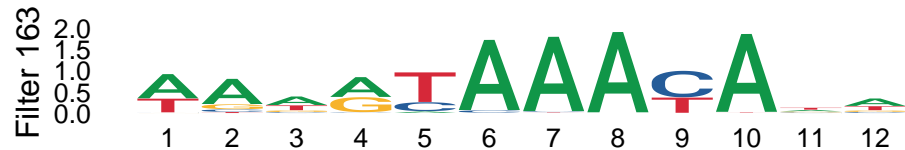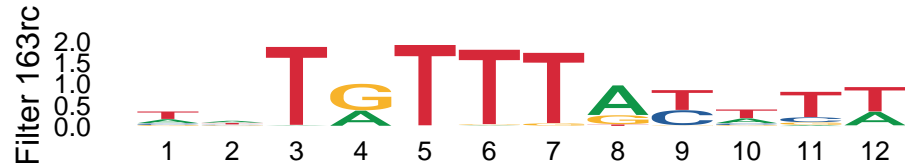

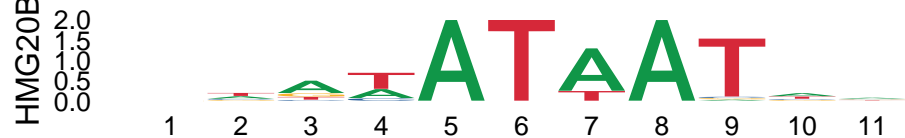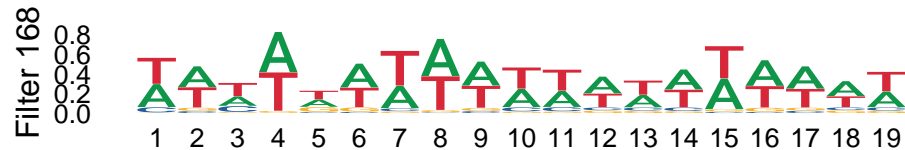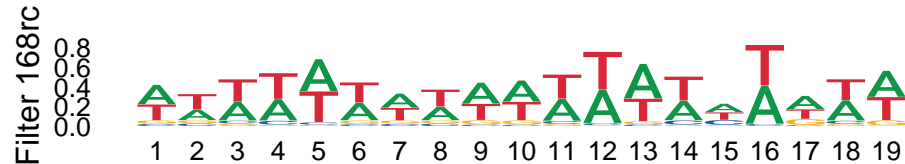

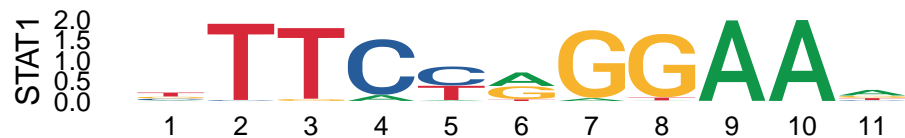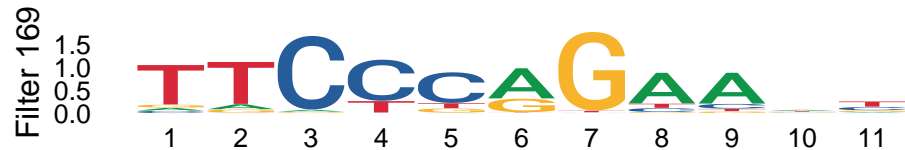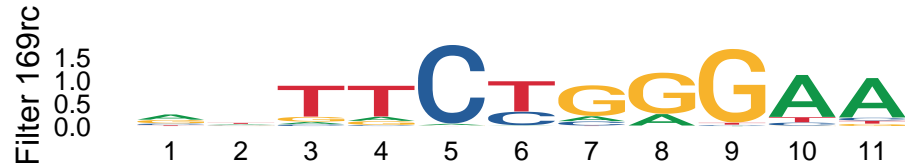

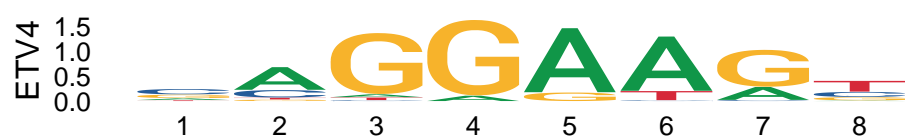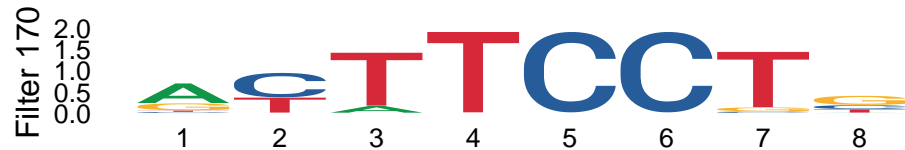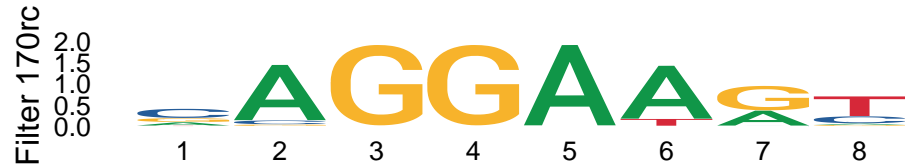

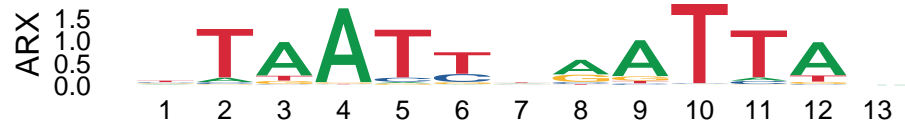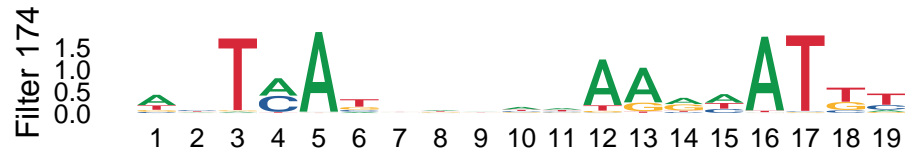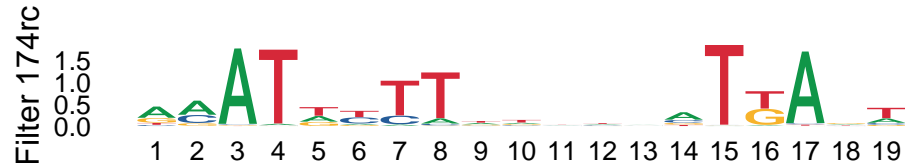

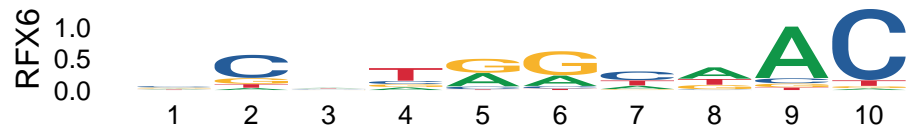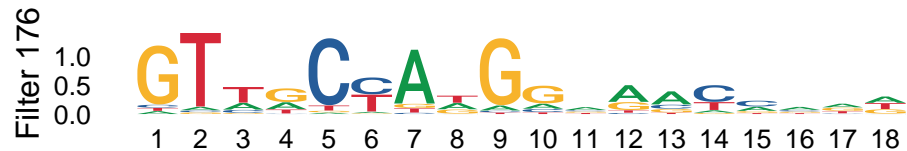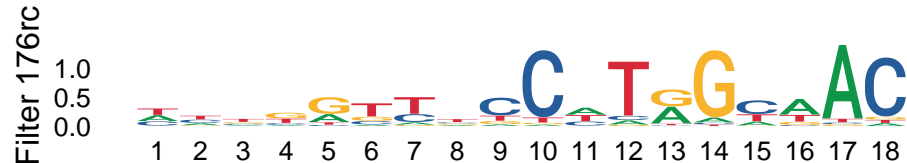



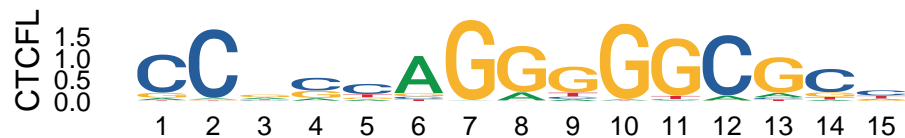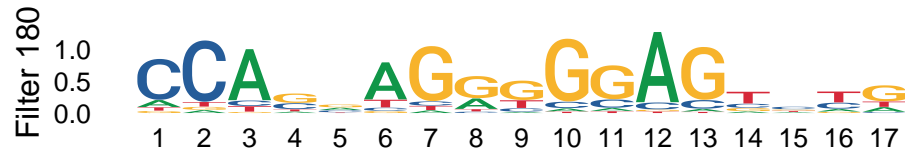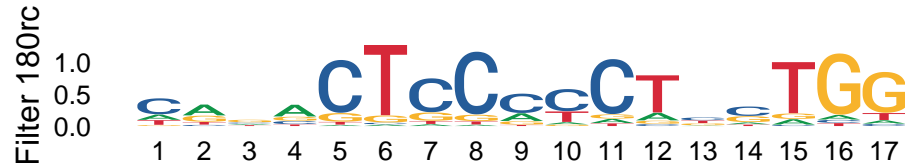

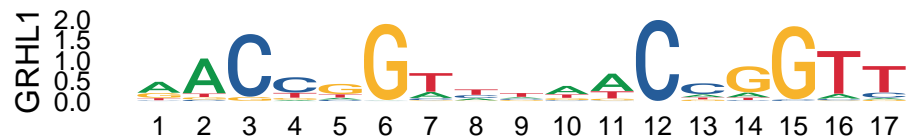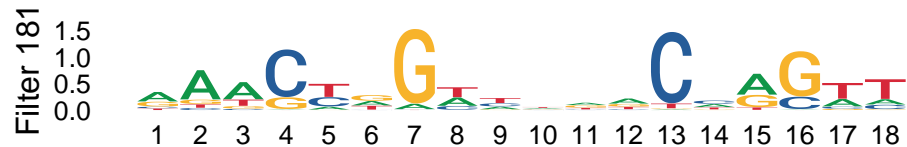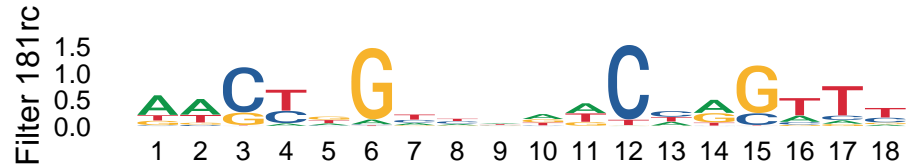

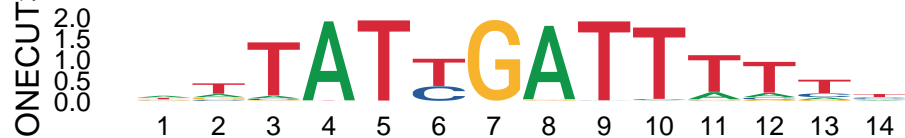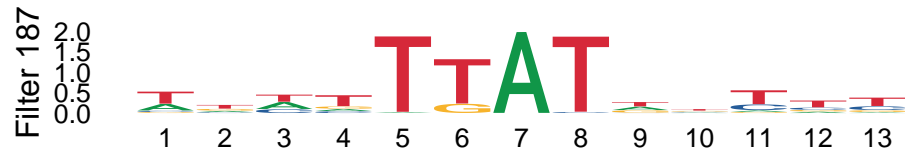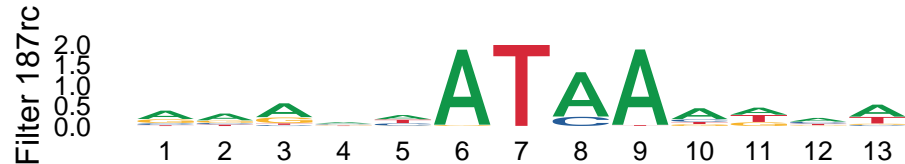

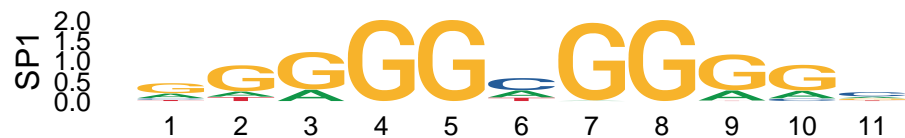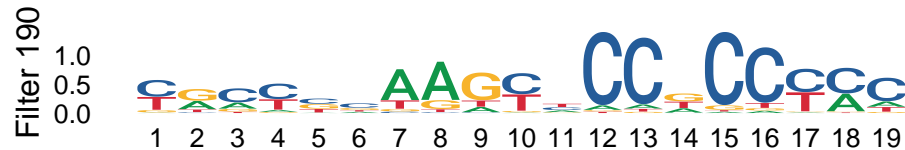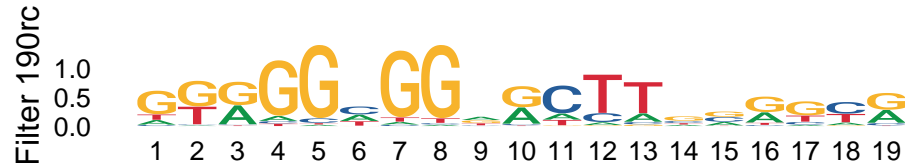

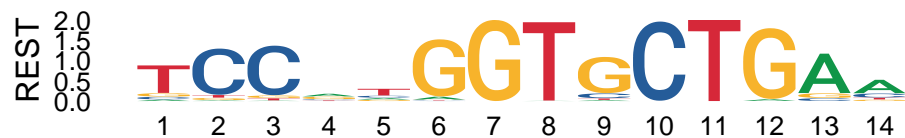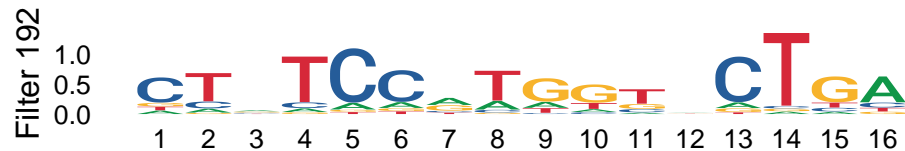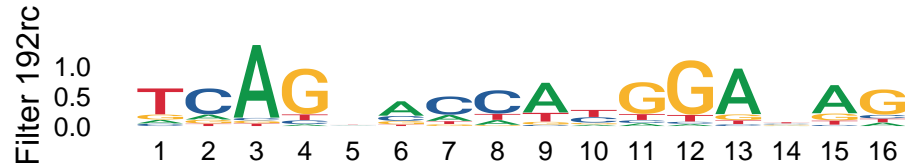

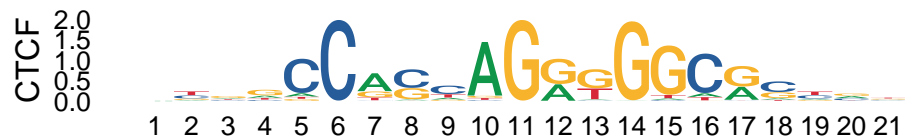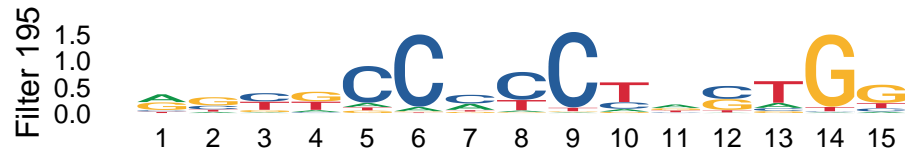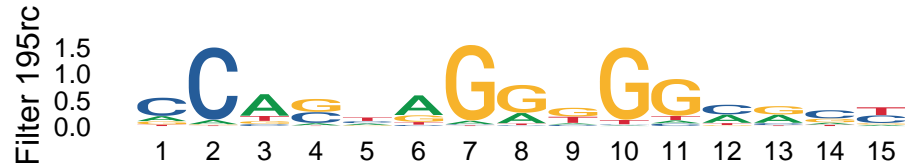

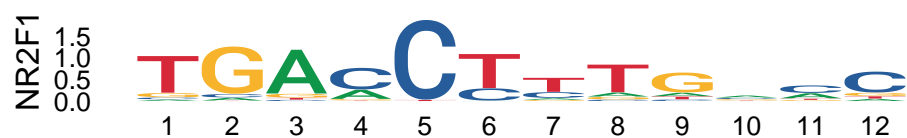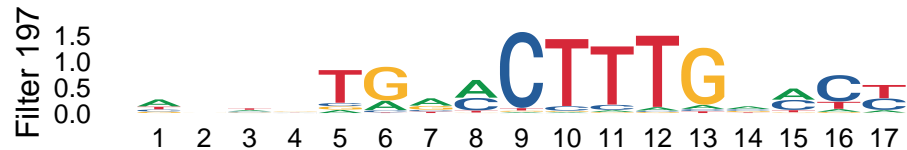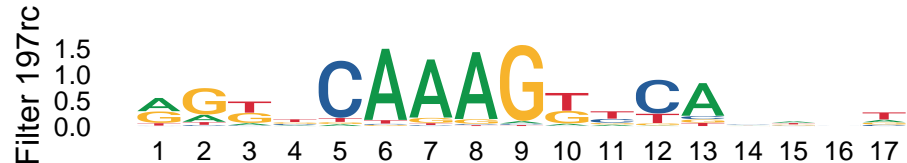

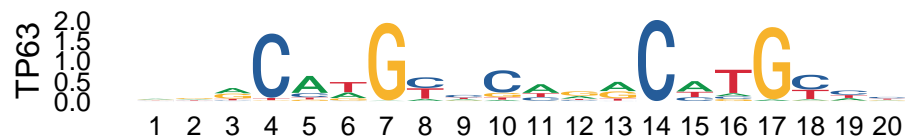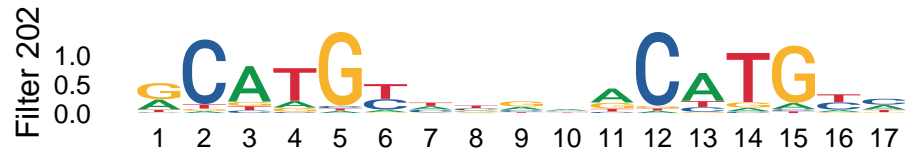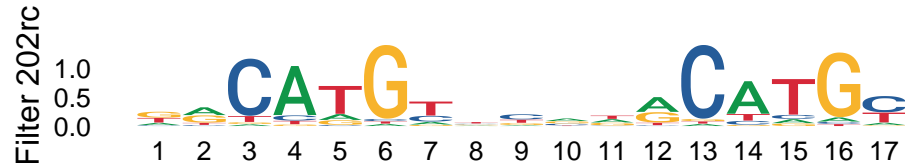

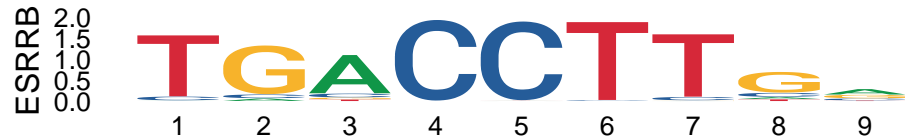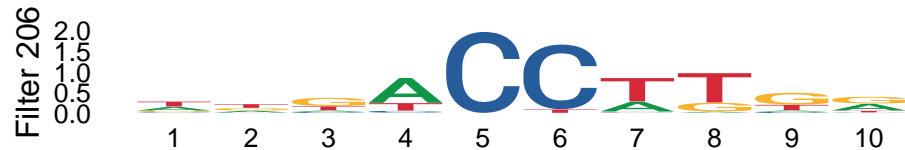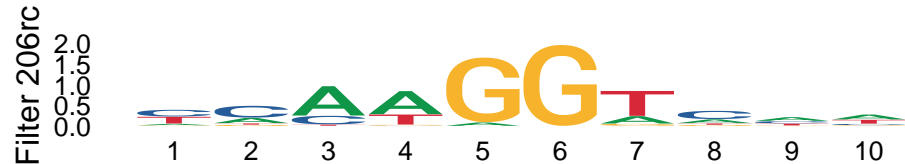

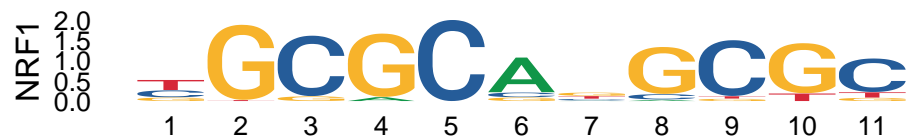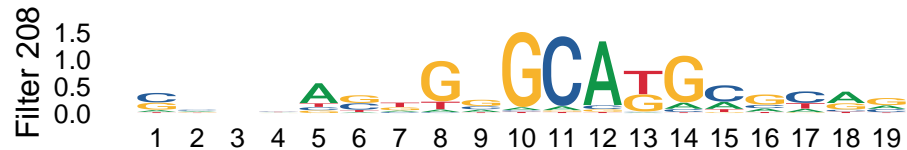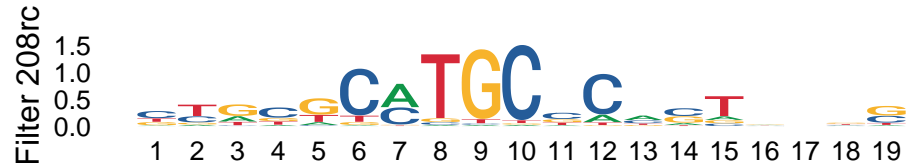

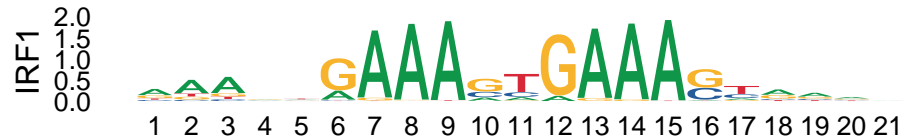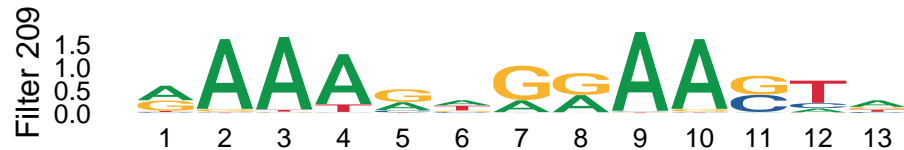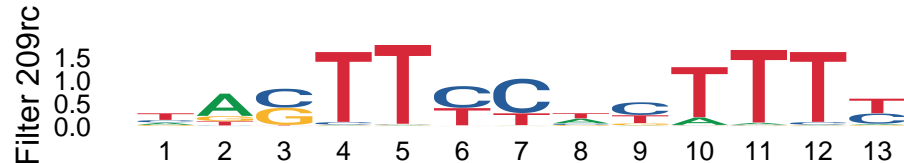

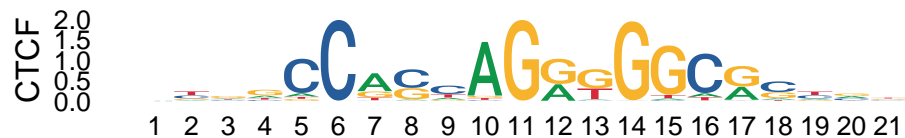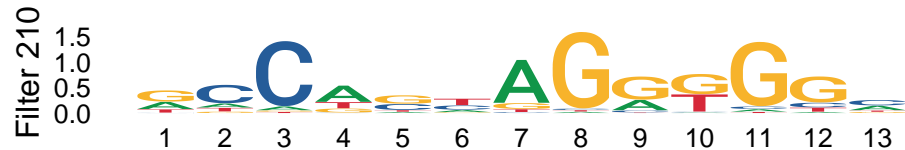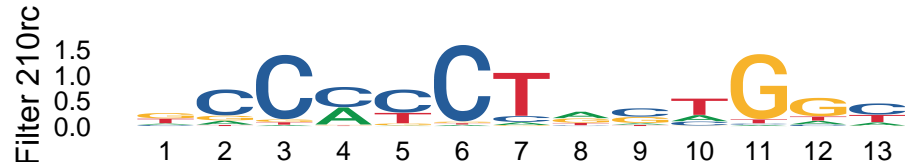

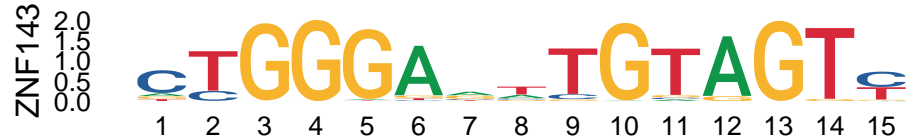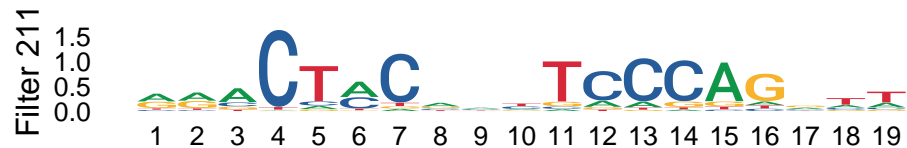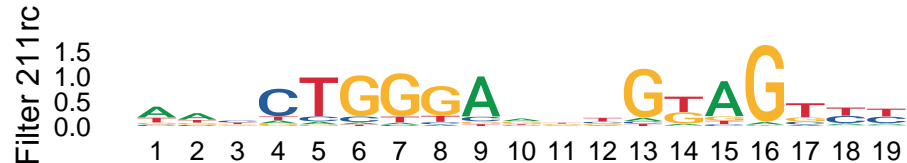

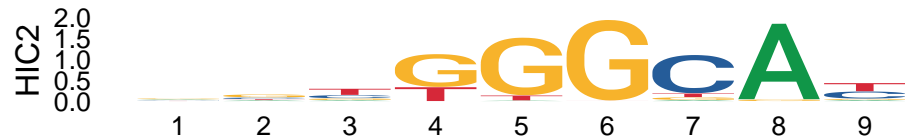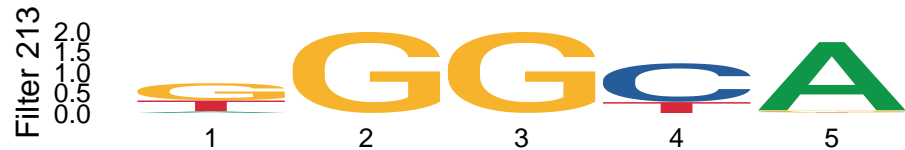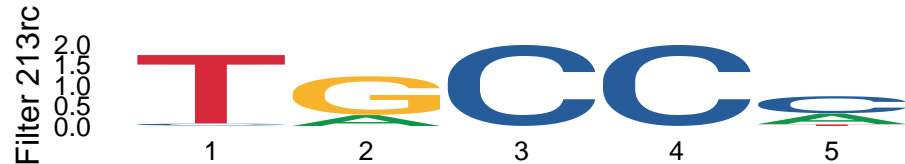

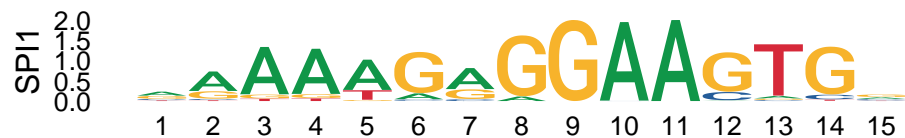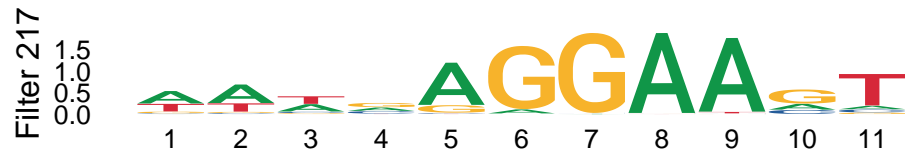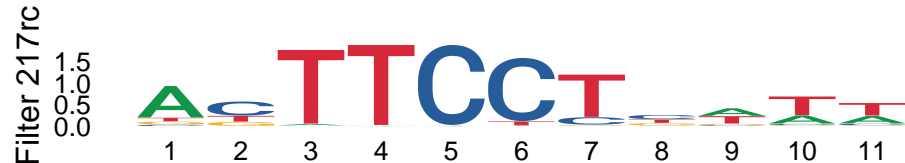

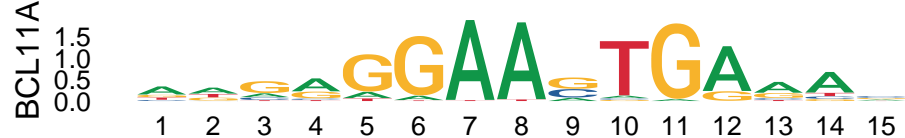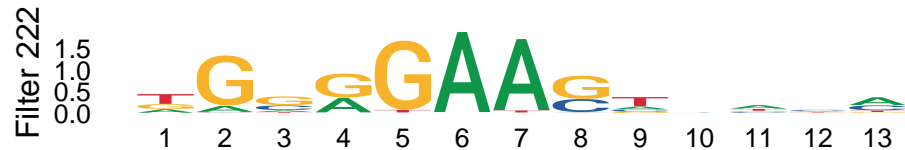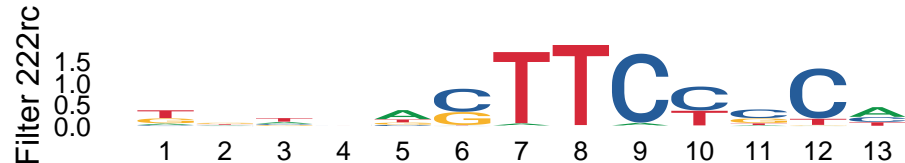

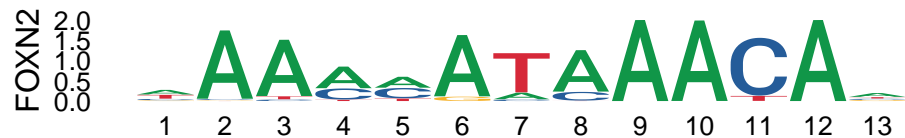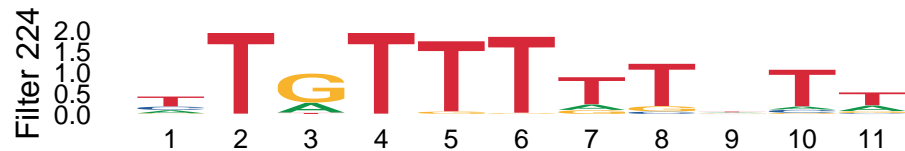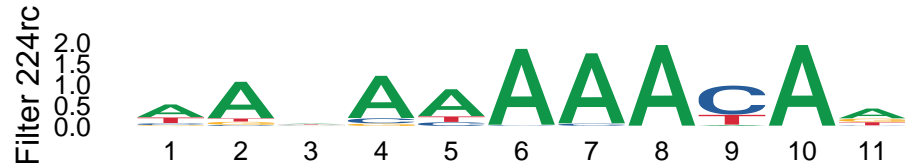

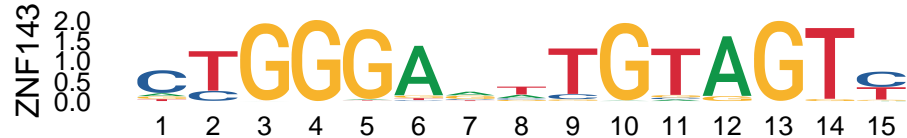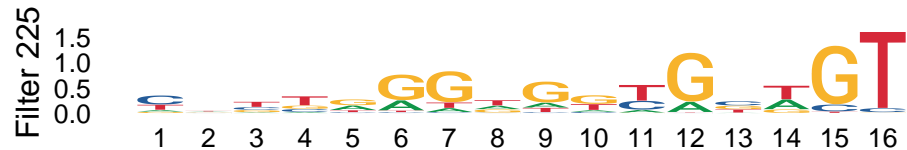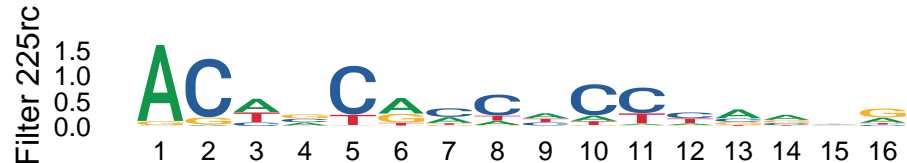

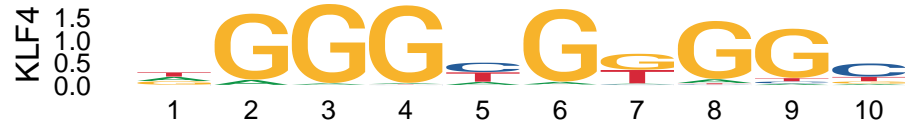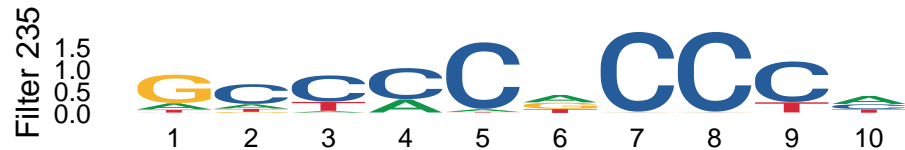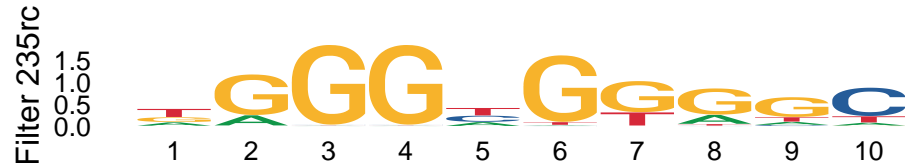

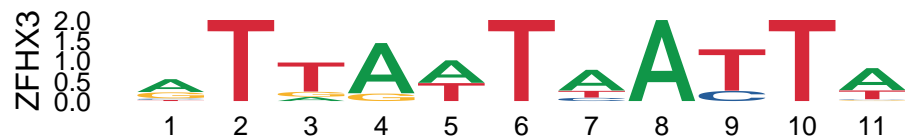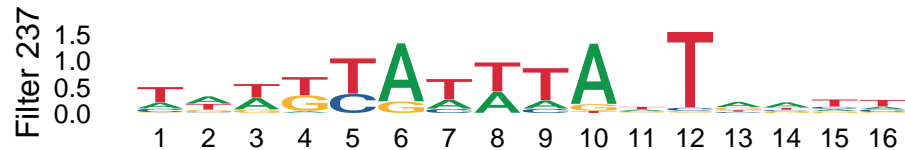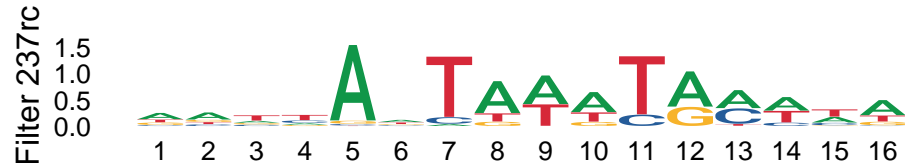

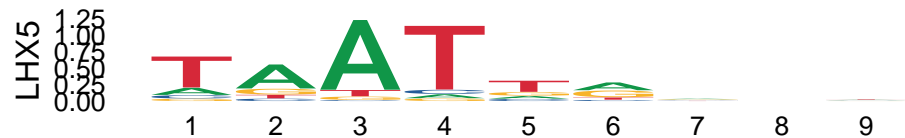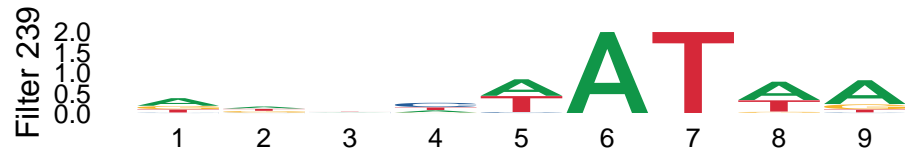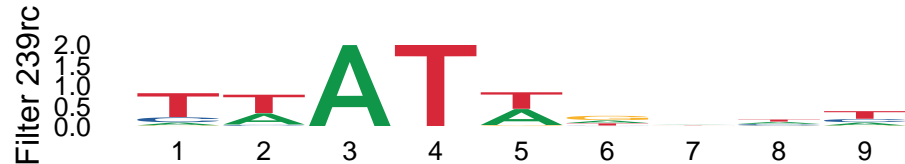

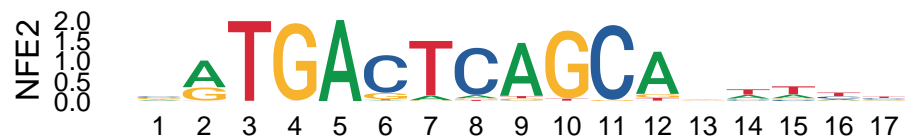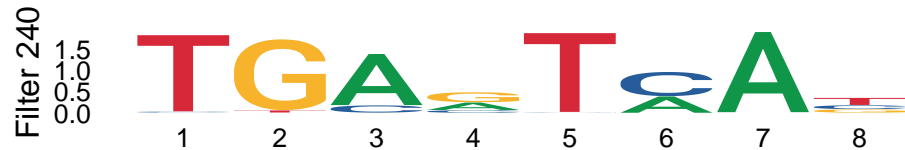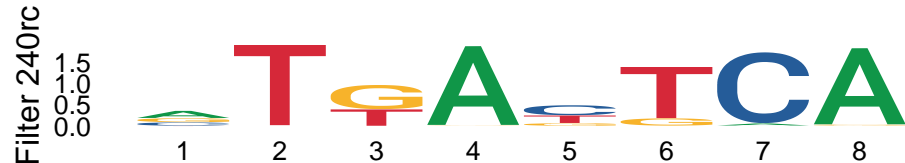

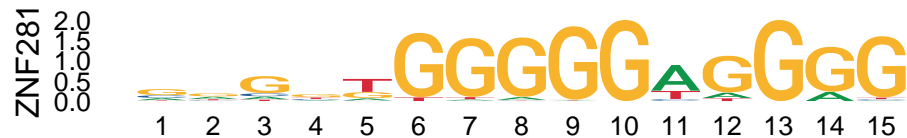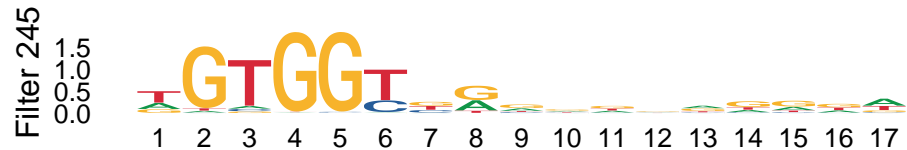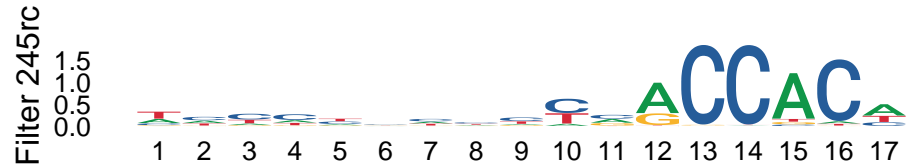

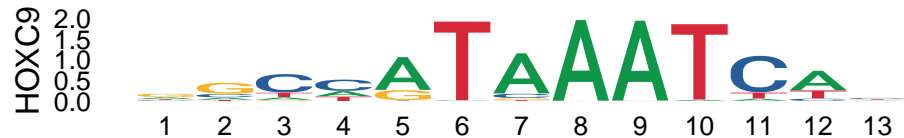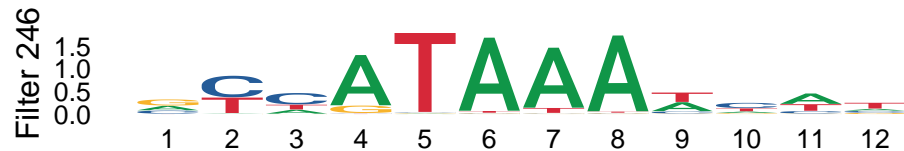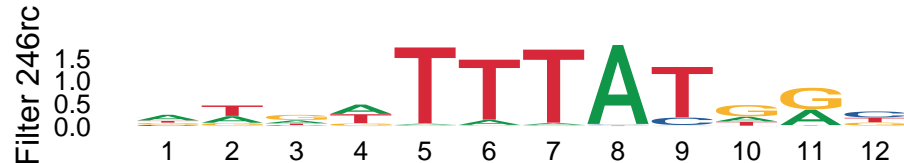

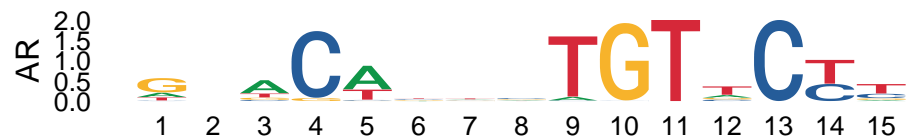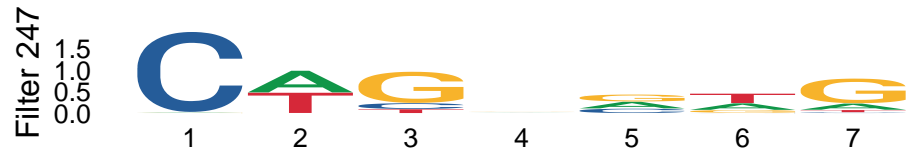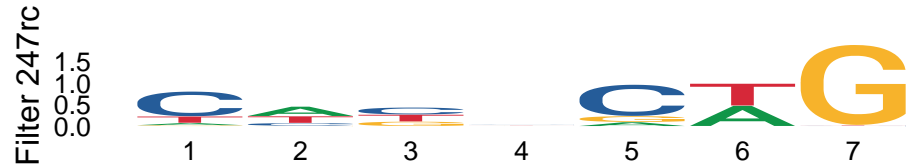



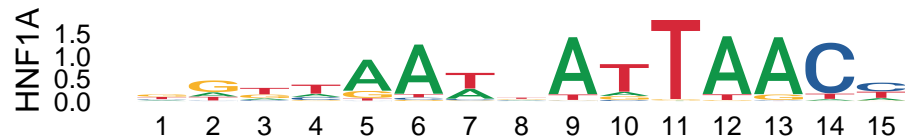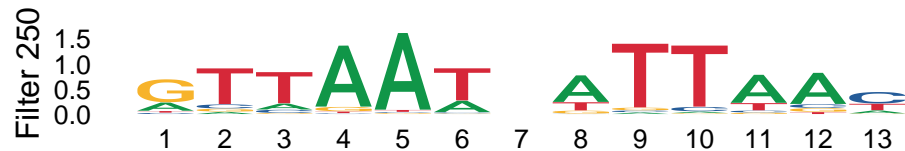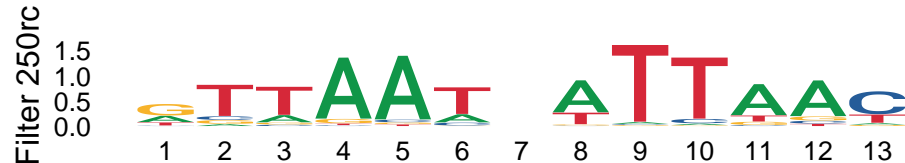

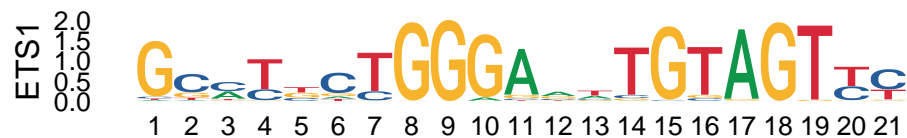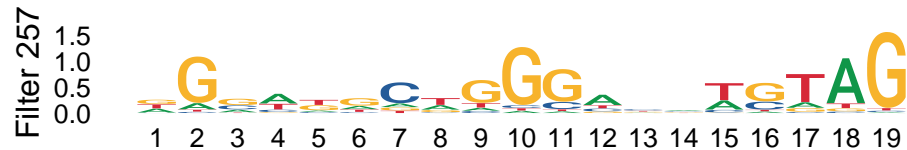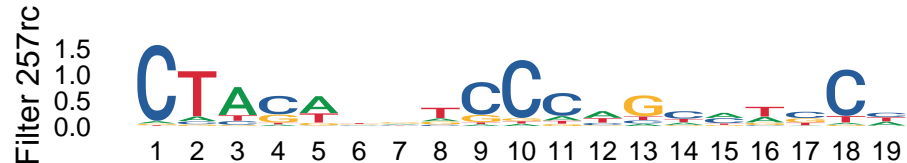

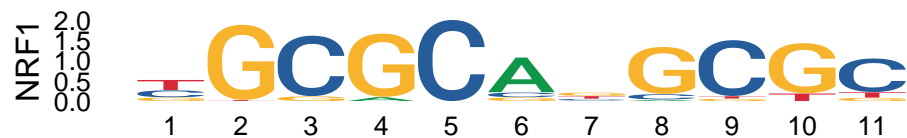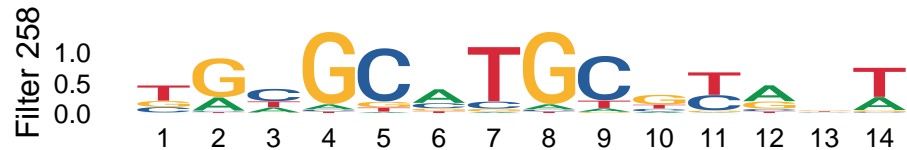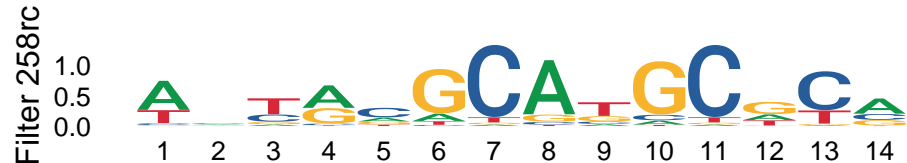

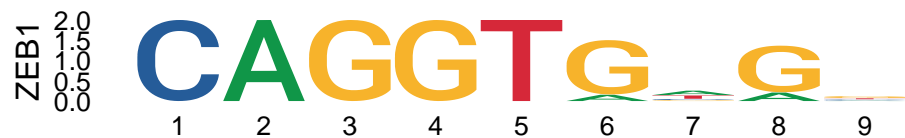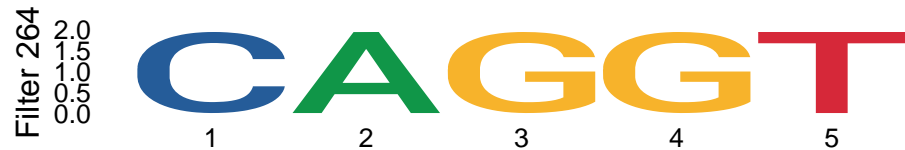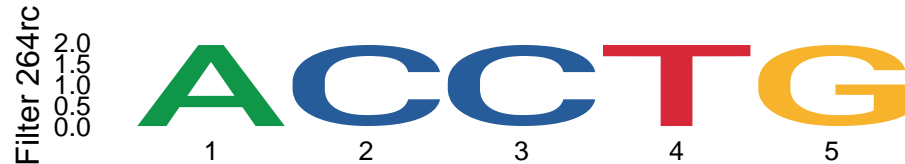

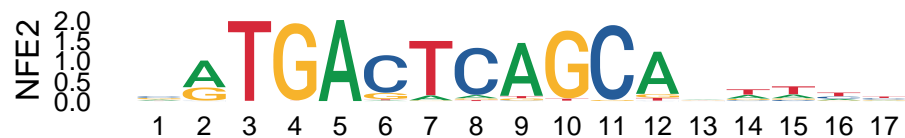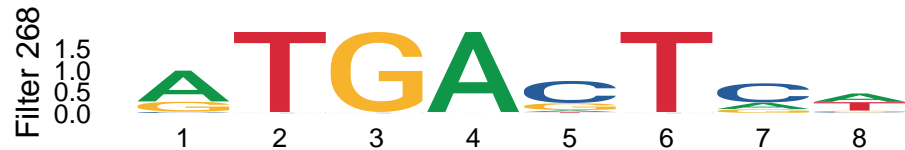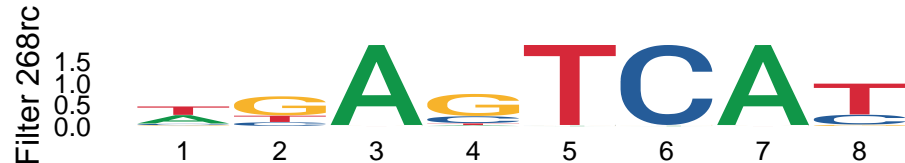

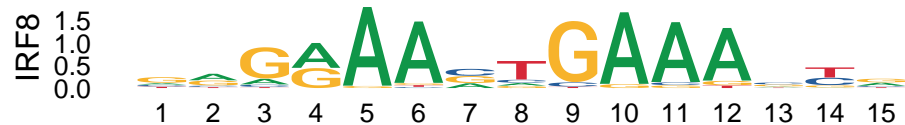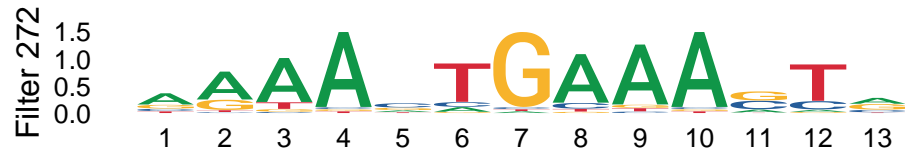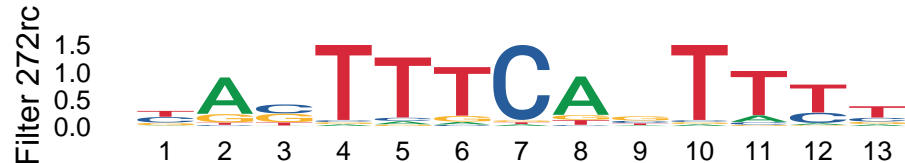

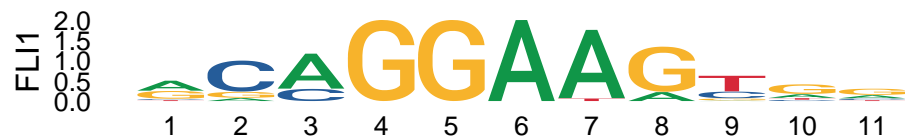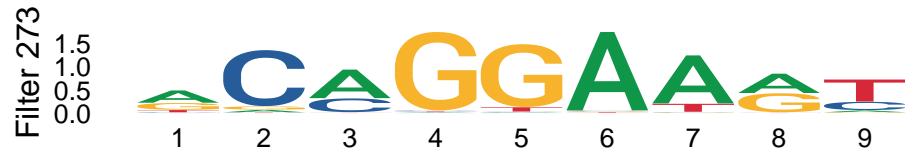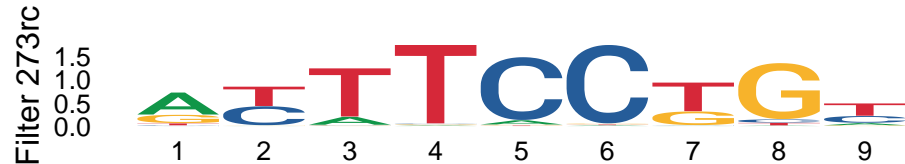

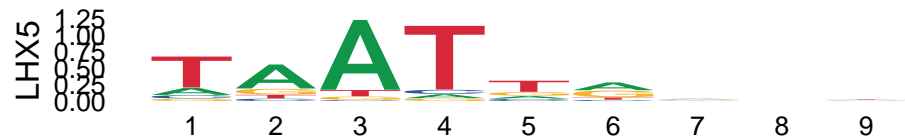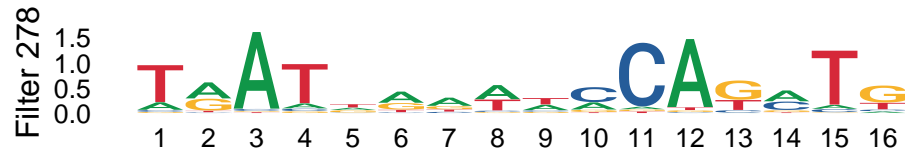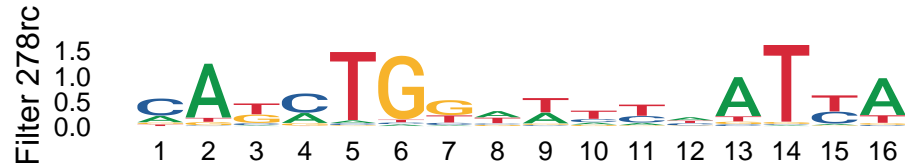

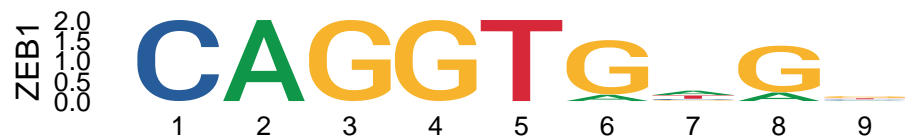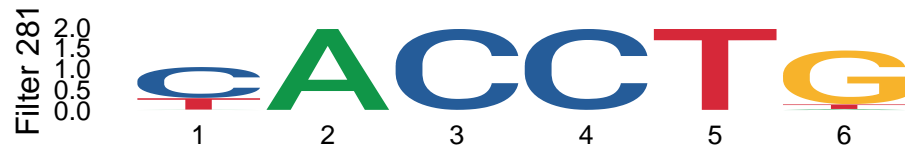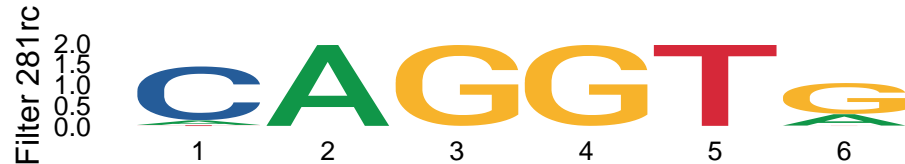

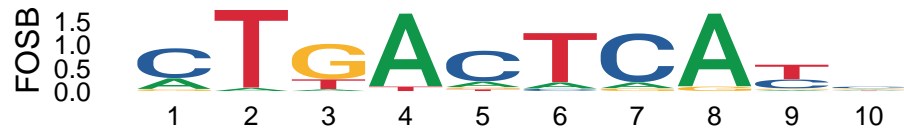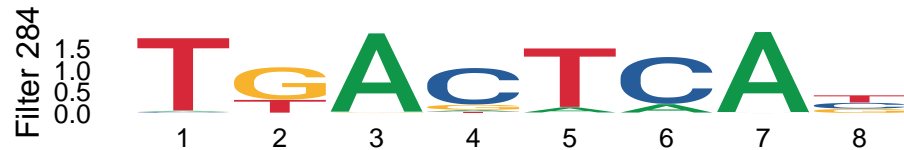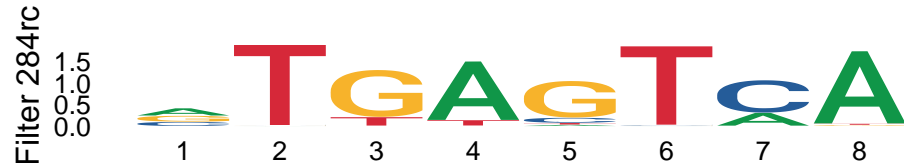

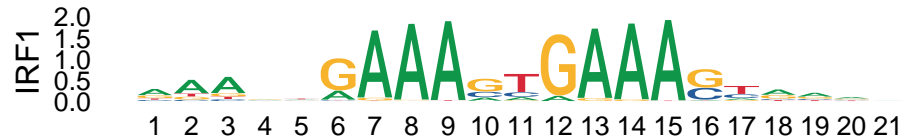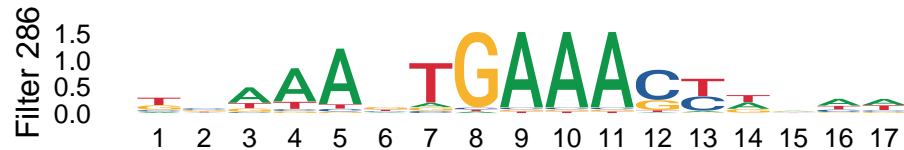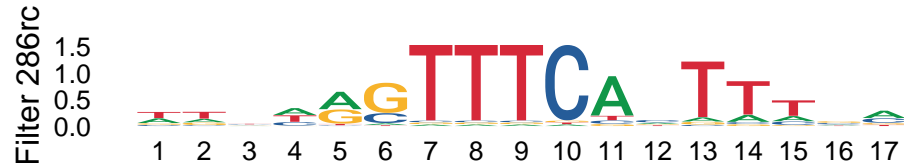

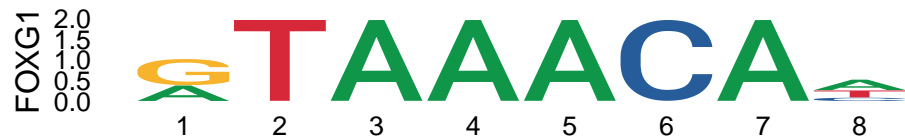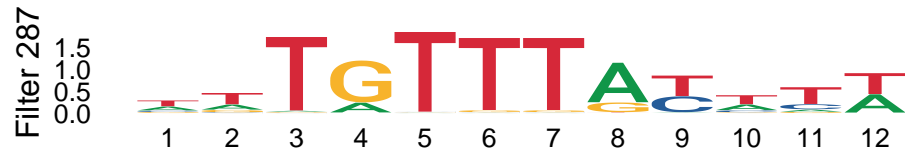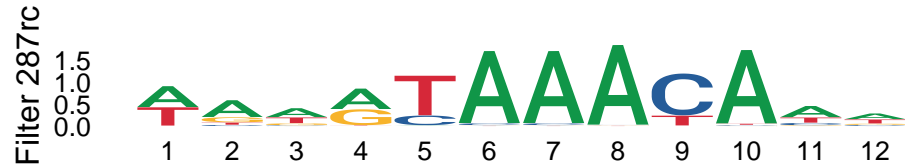

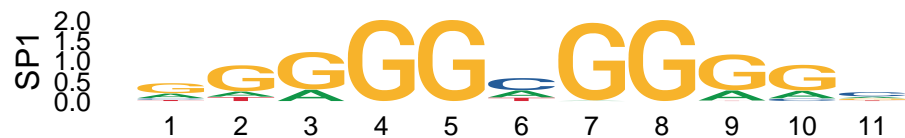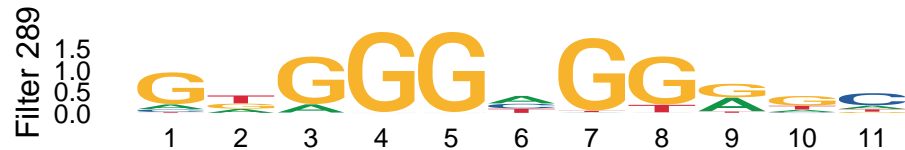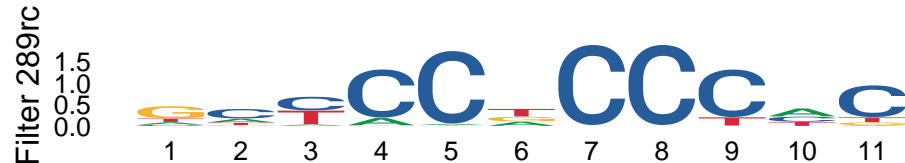

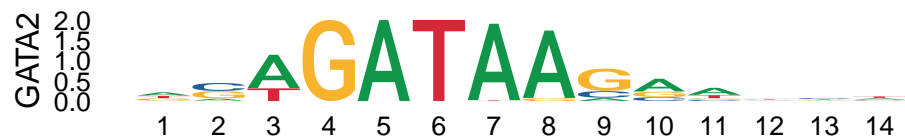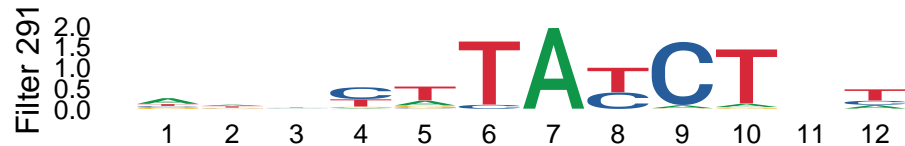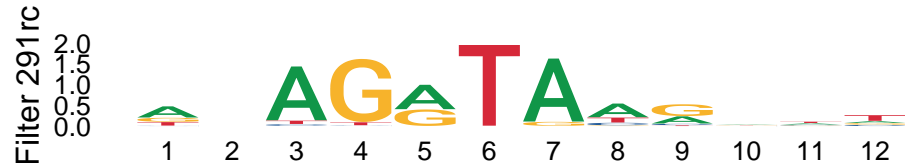

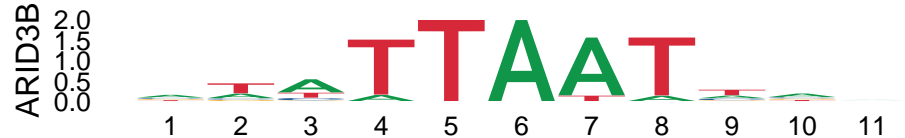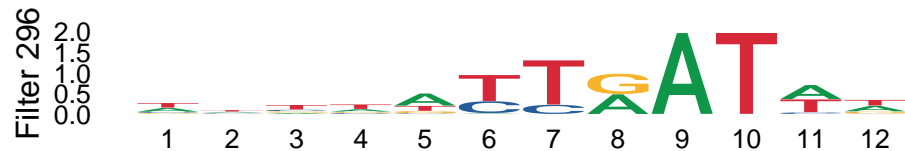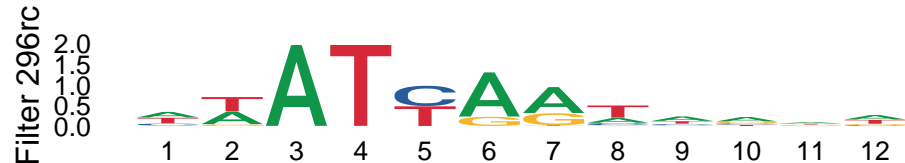

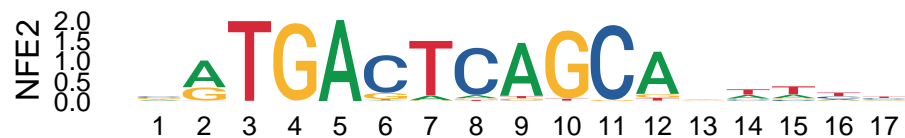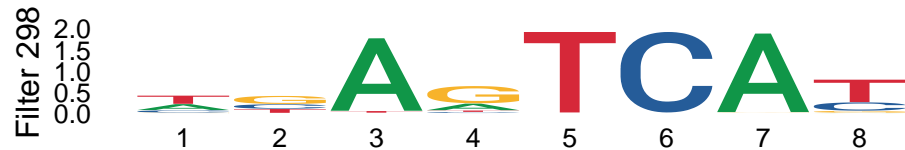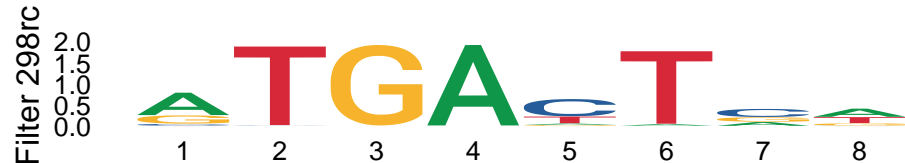

Supplement: gkaa1137_Supplemental_Files [file gkaa1137_supplemental_files.zip › Supplementary Figure S1.pdf]

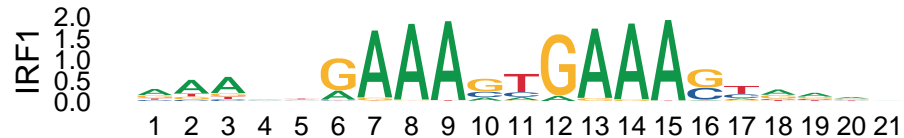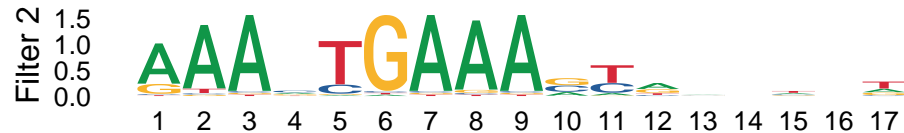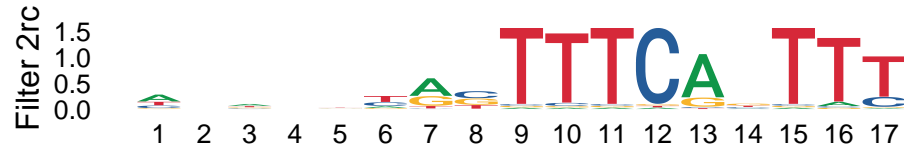

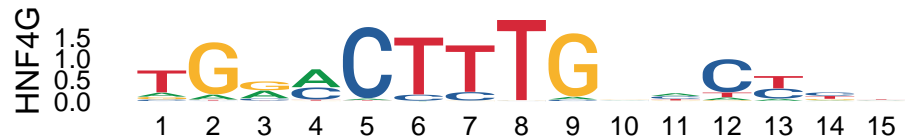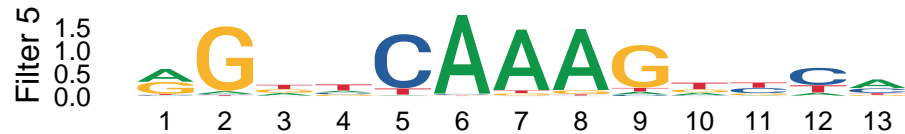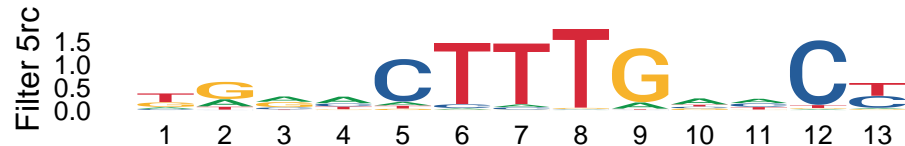

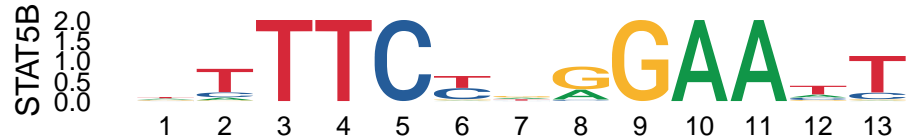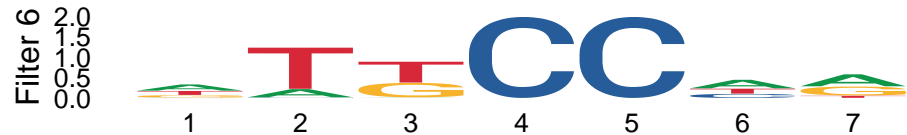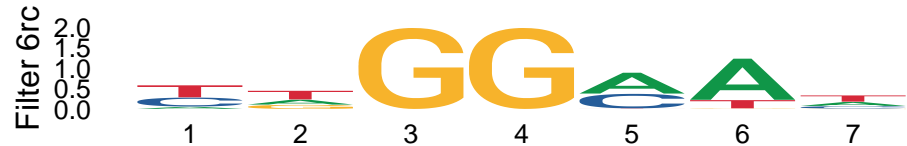



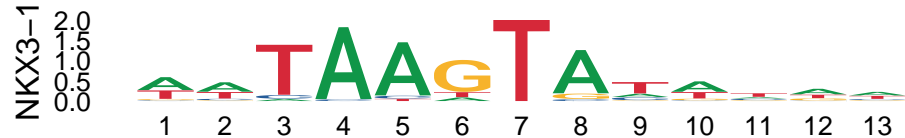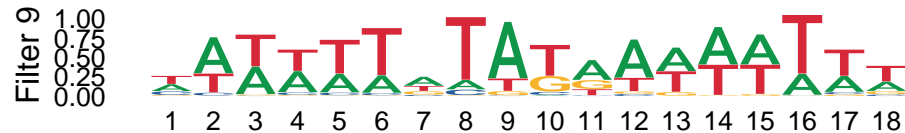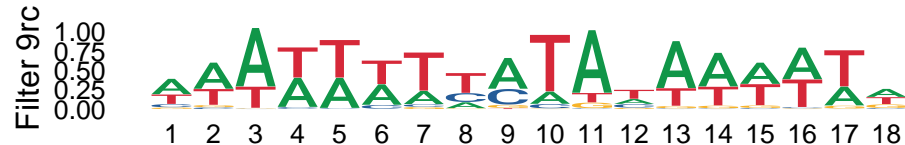

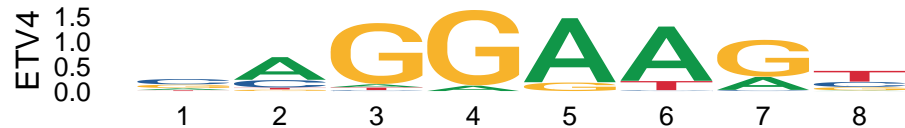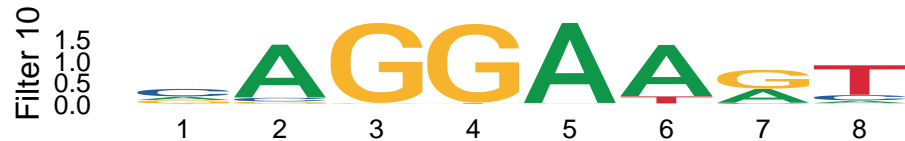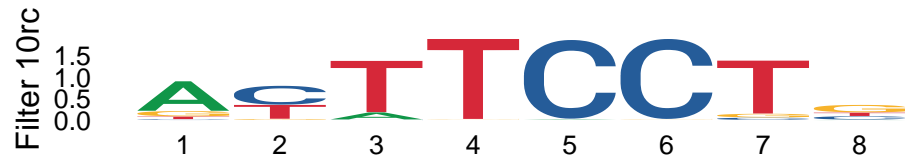

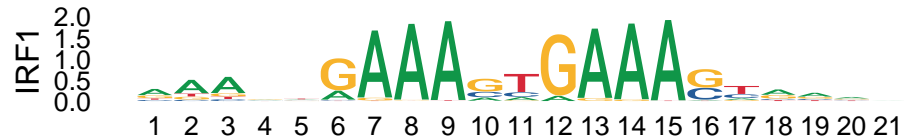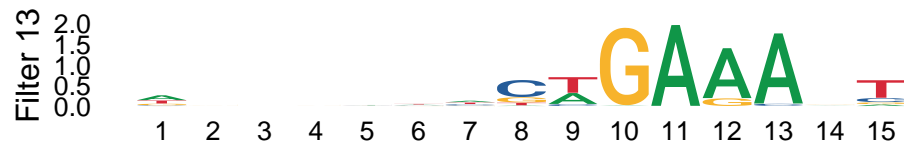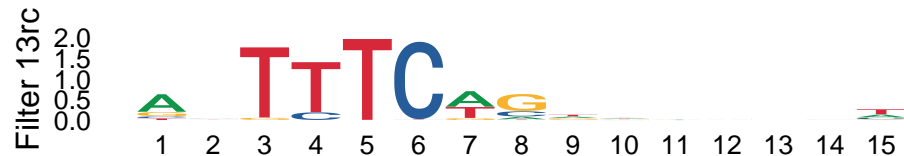

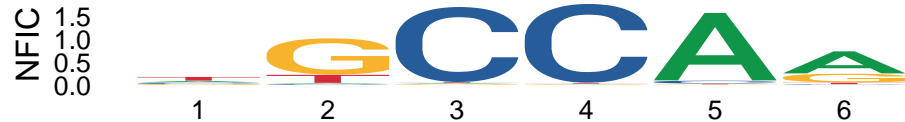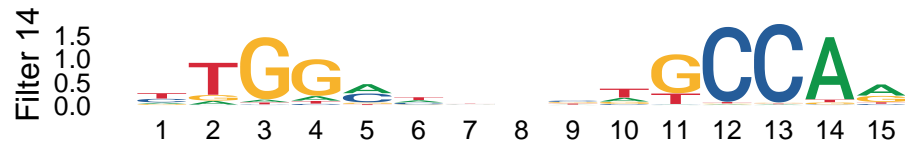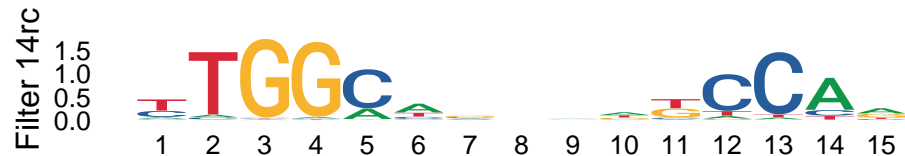

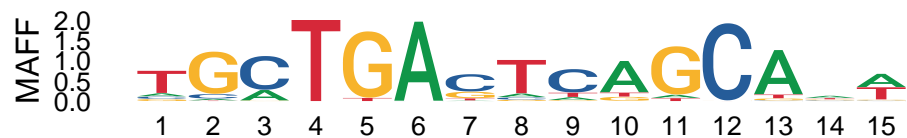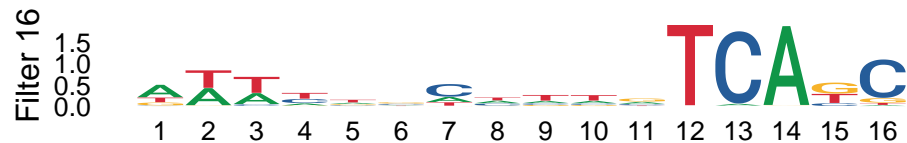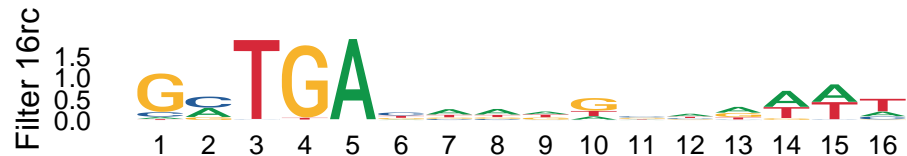

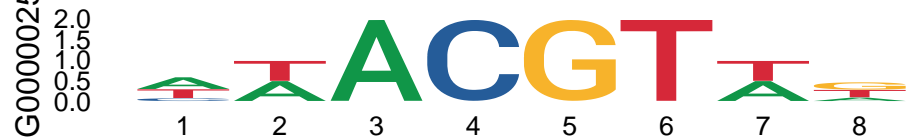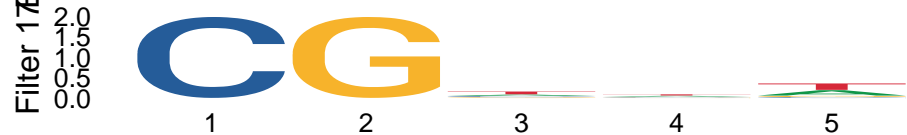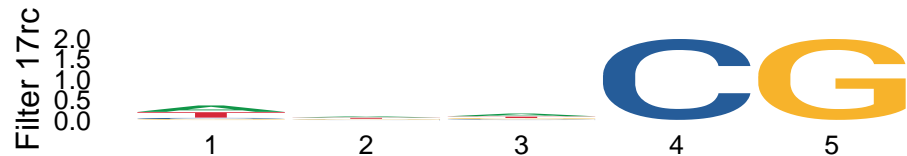

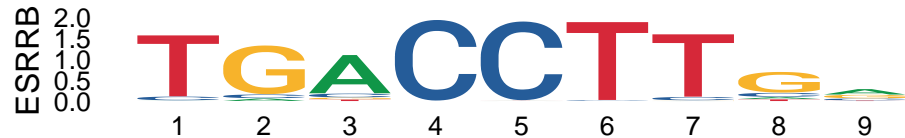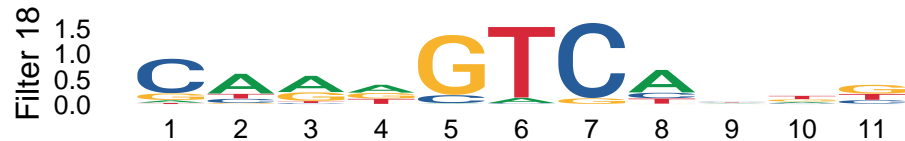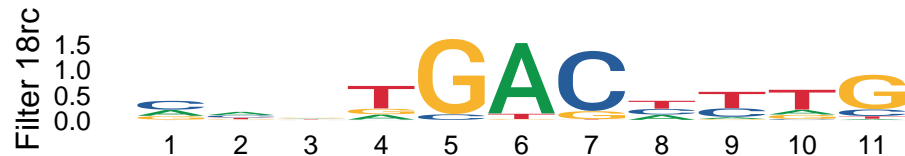

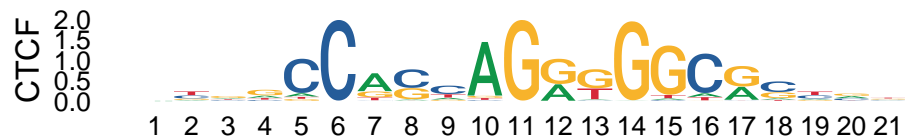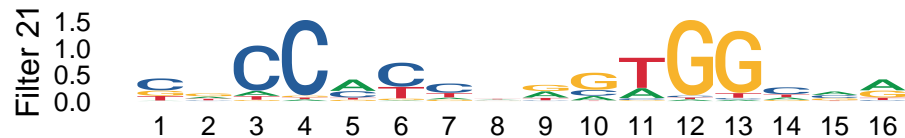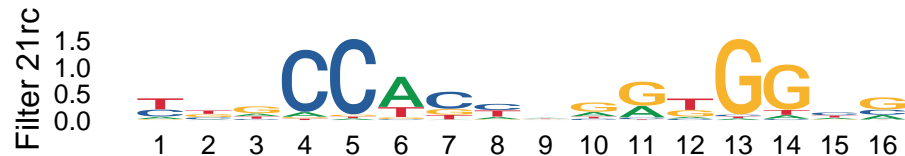

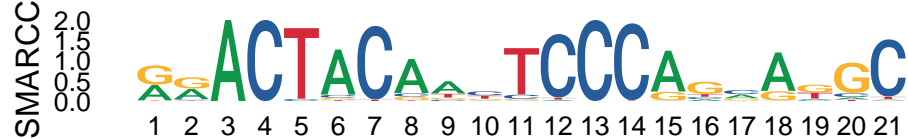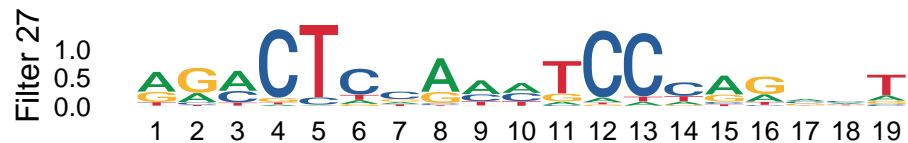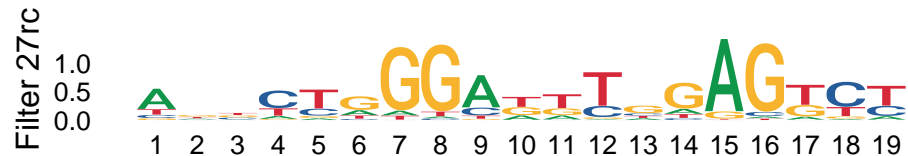

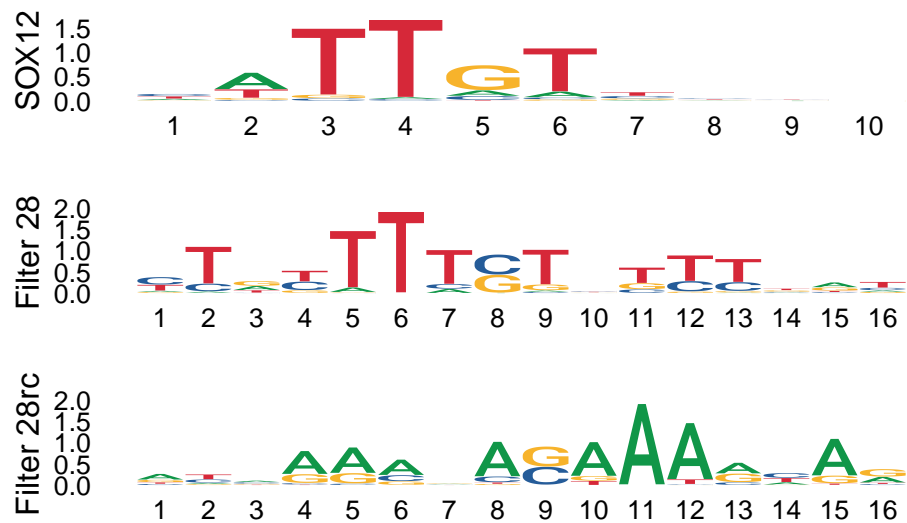

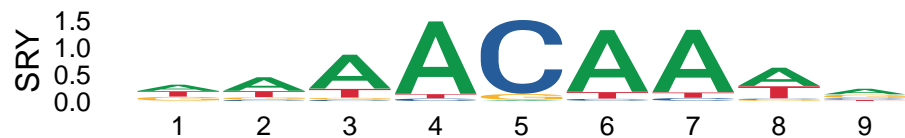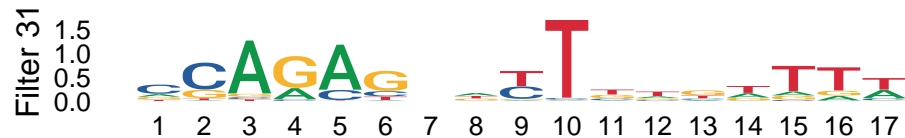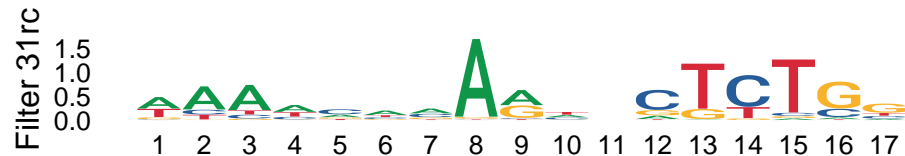



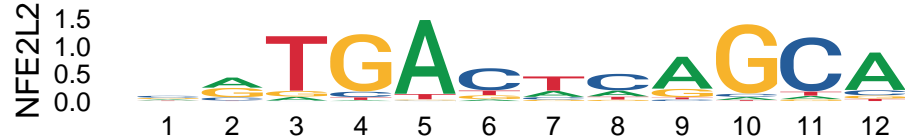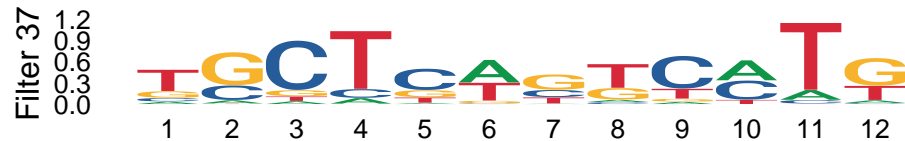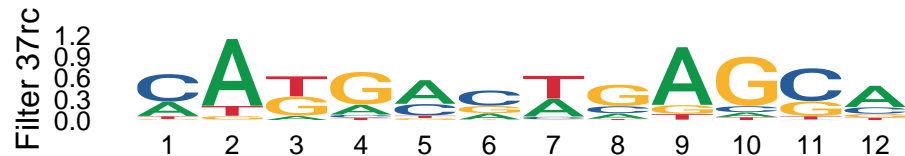

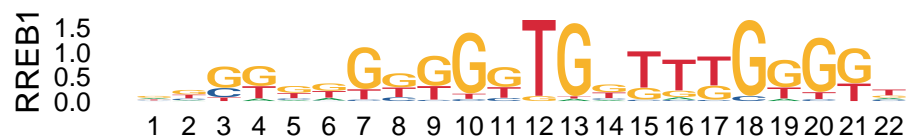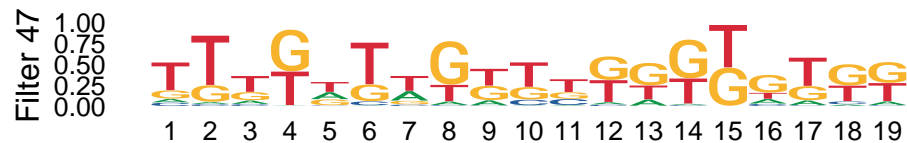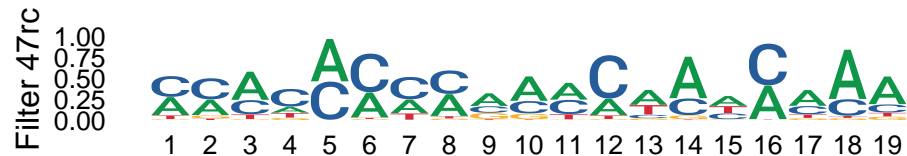

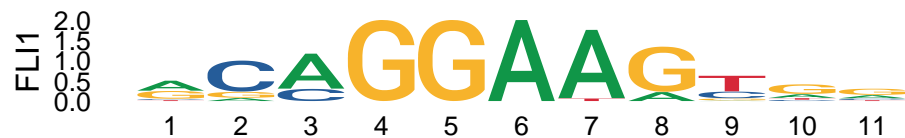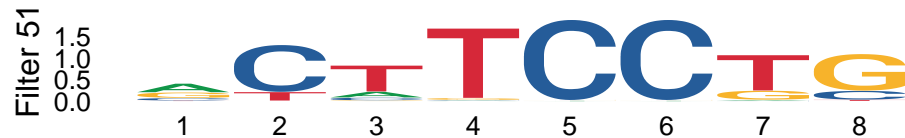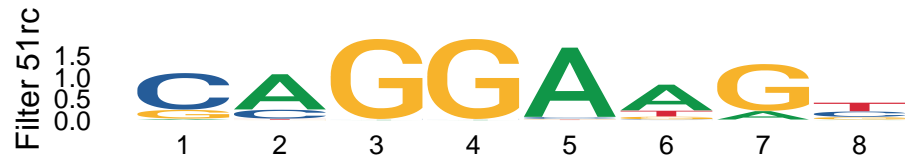

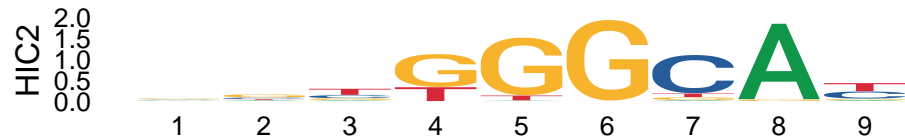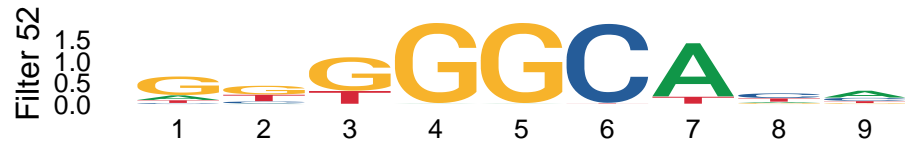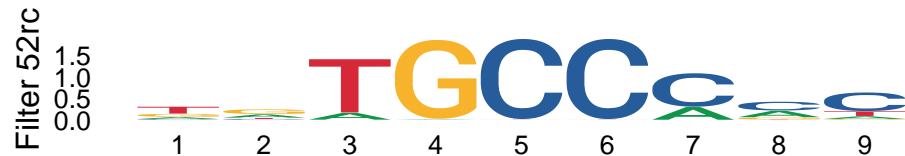

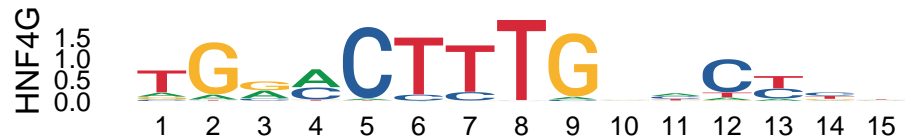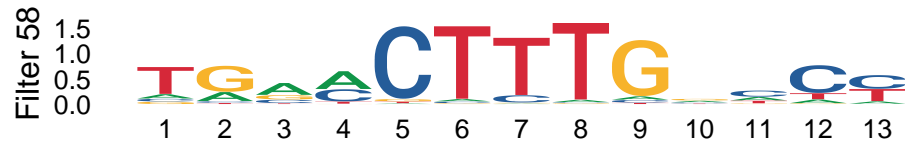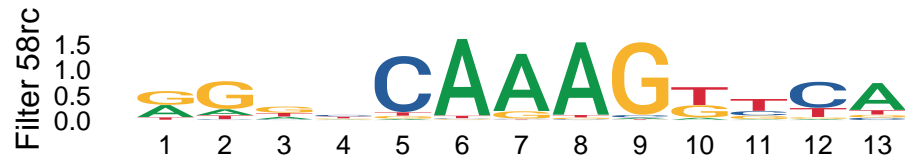

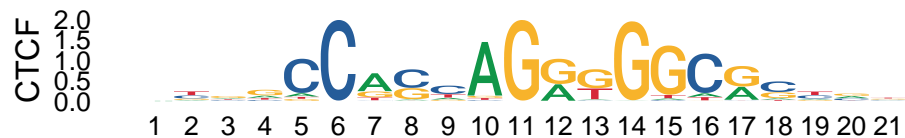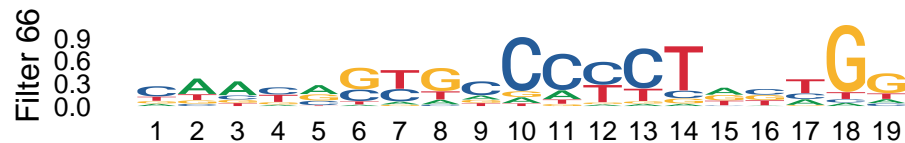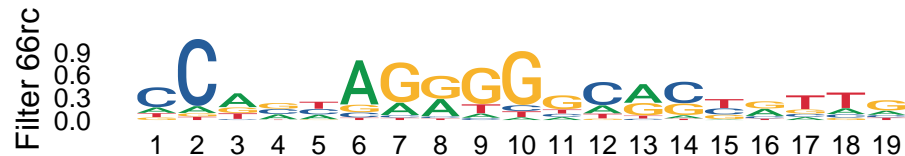

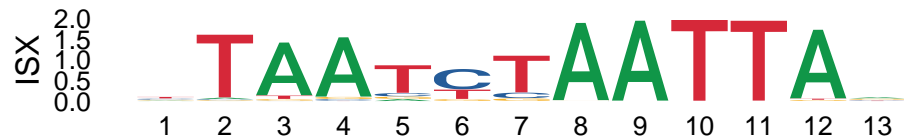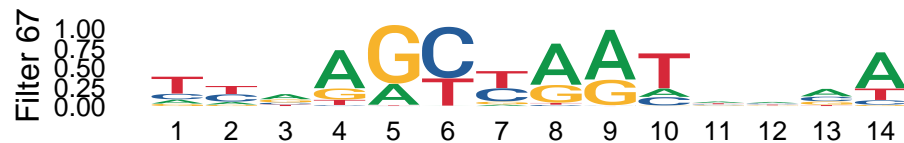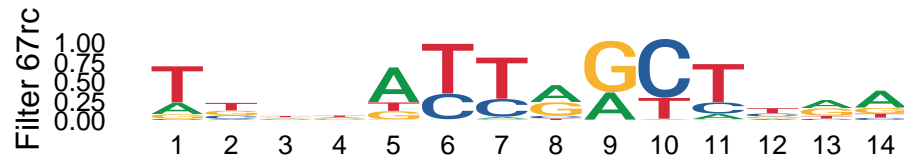

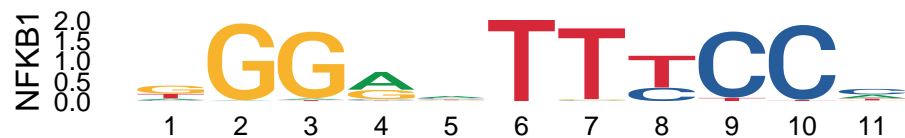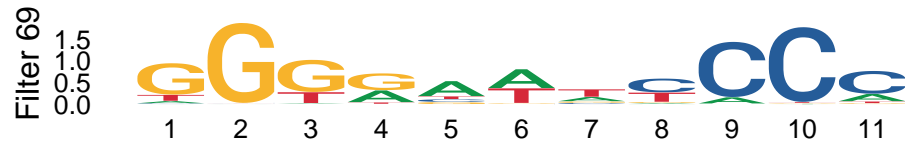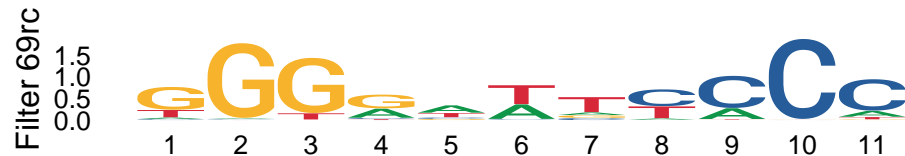

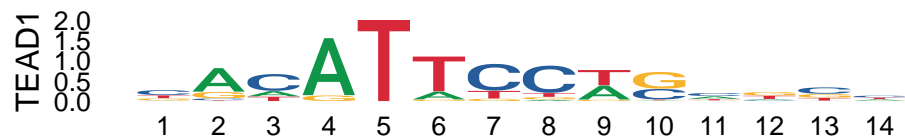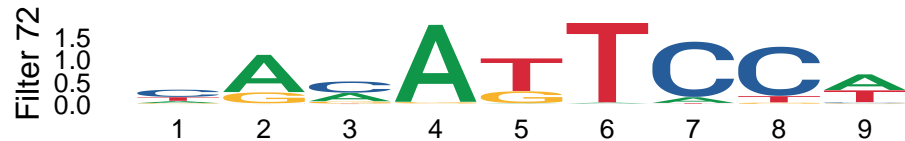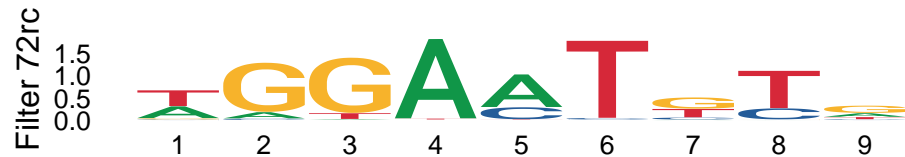

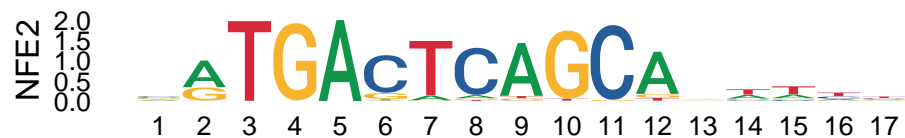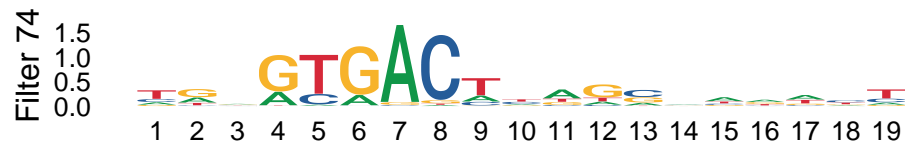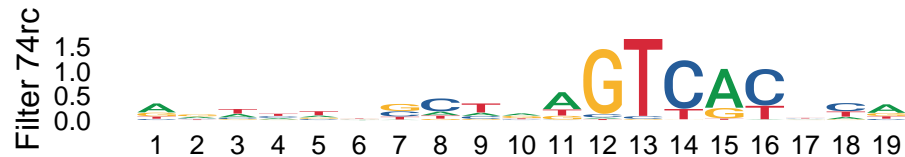

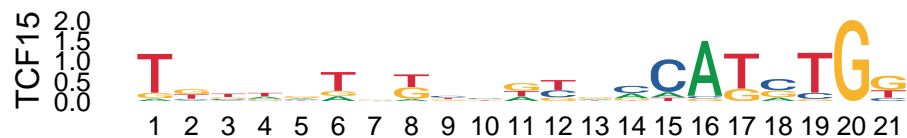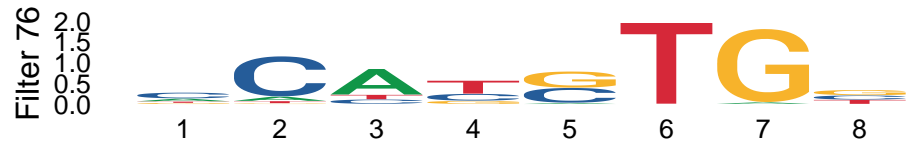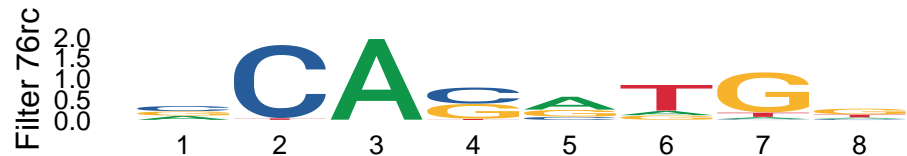

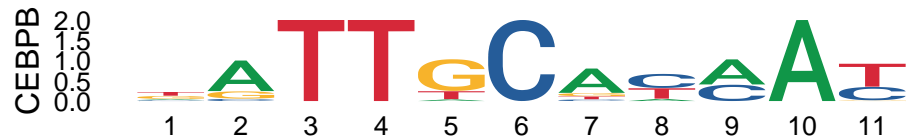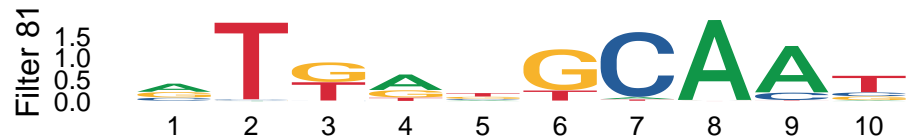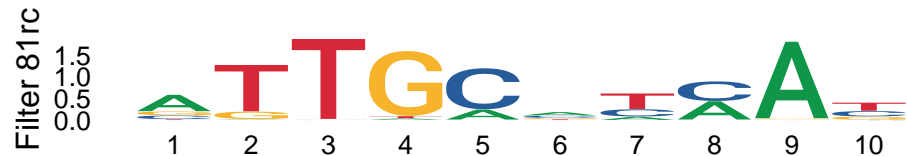

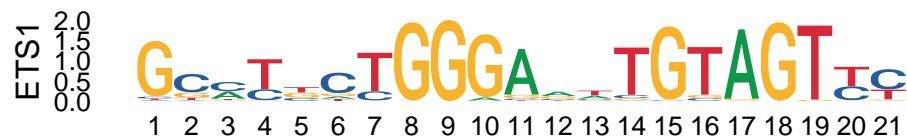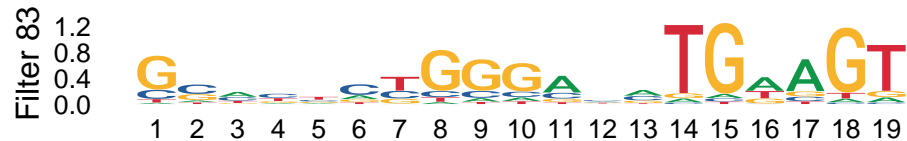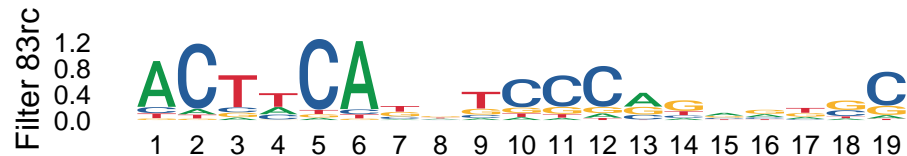

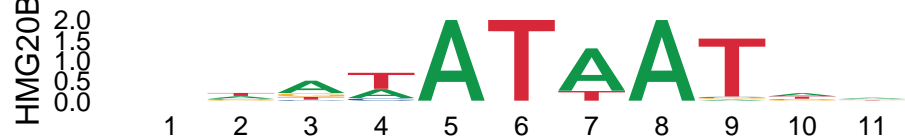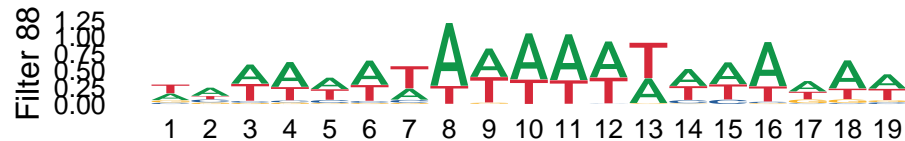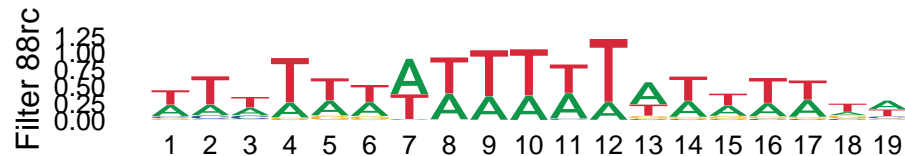

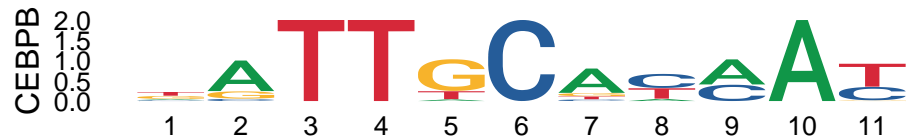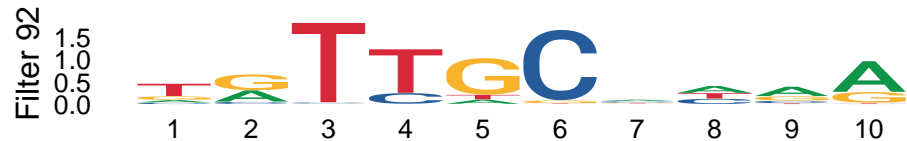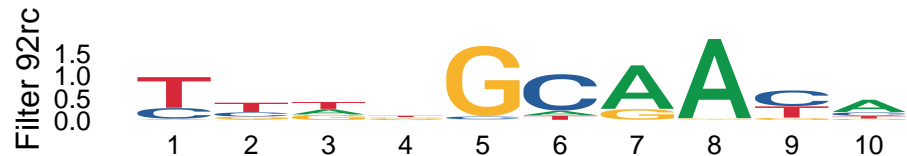

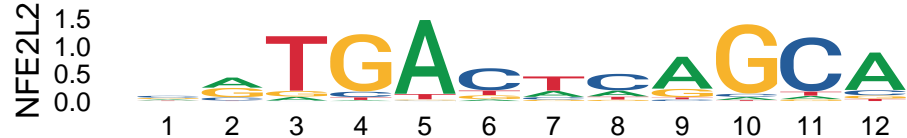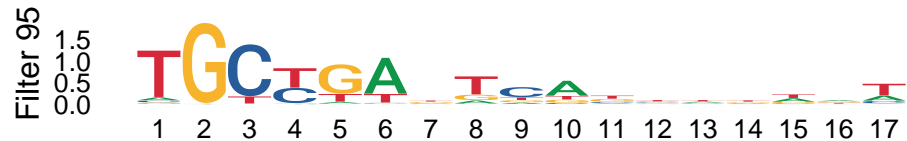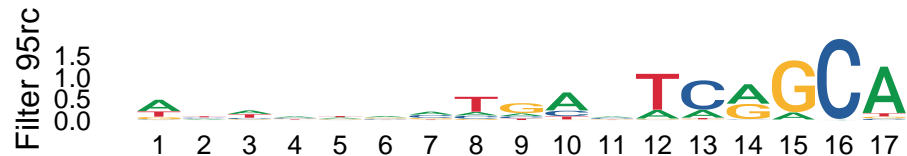

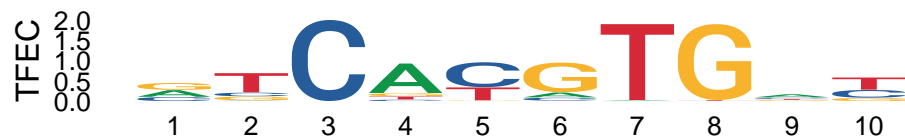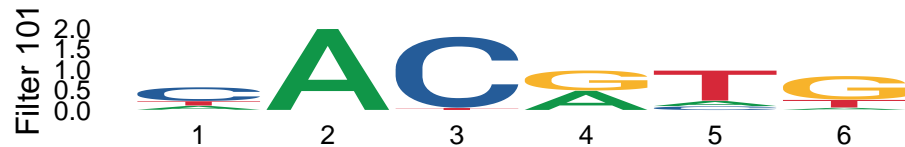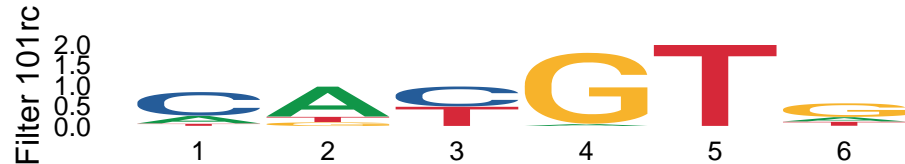

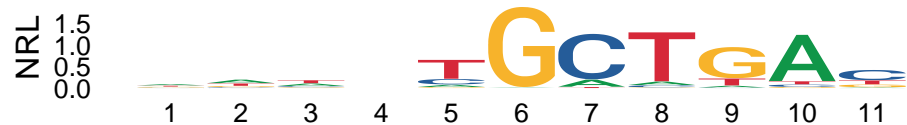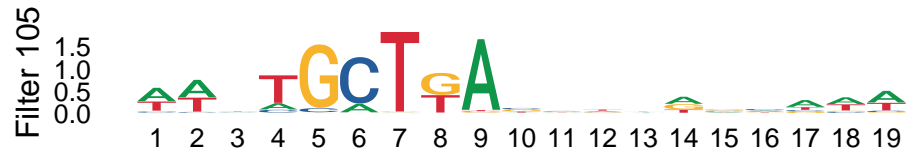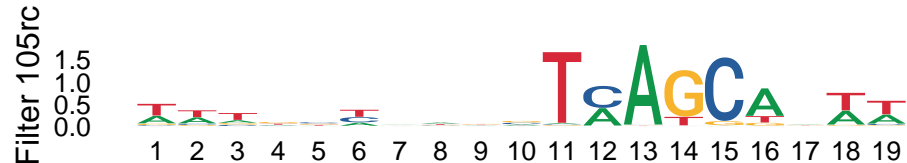

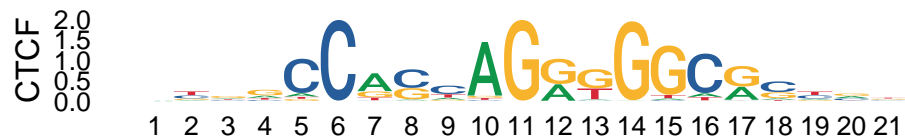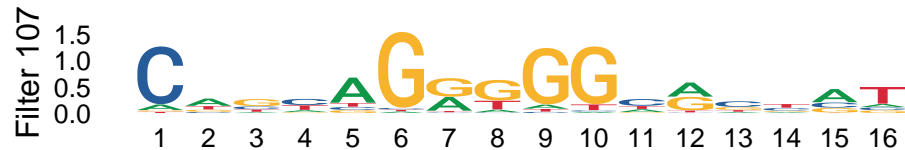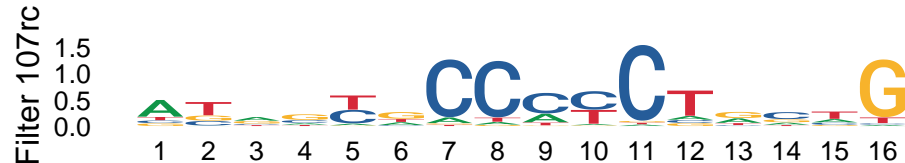

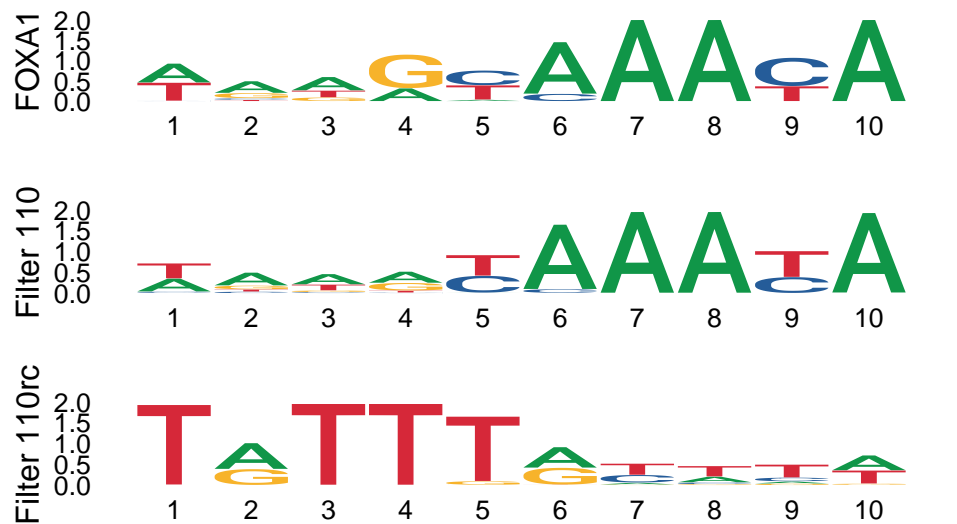

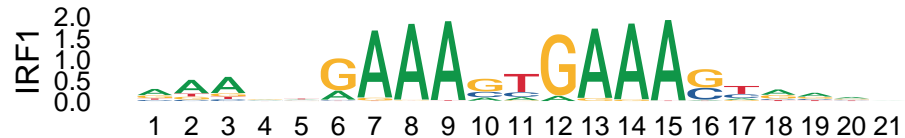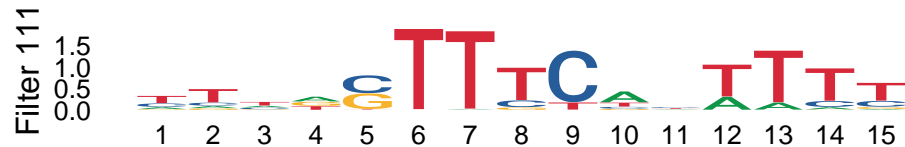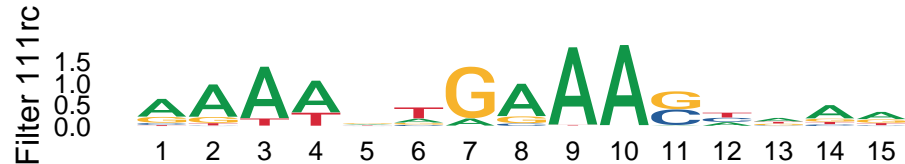

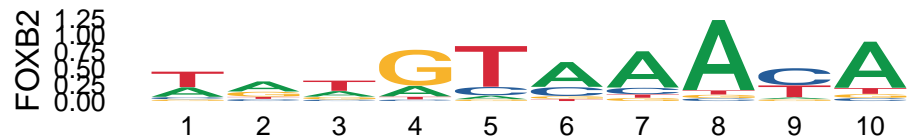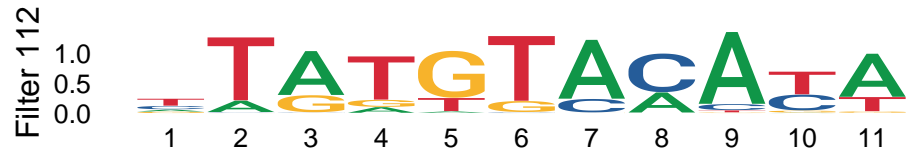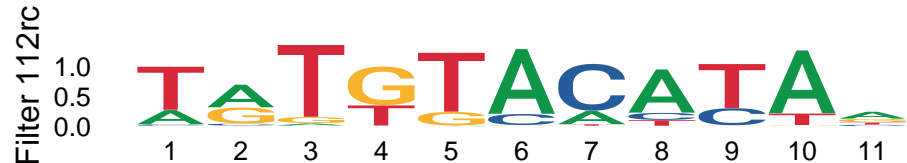

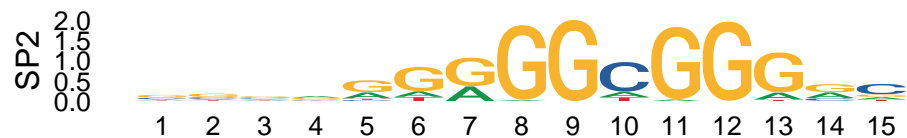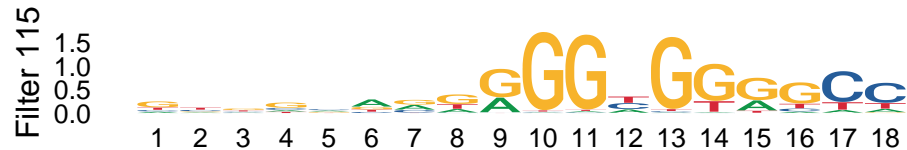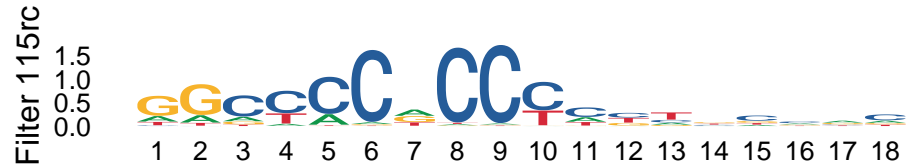

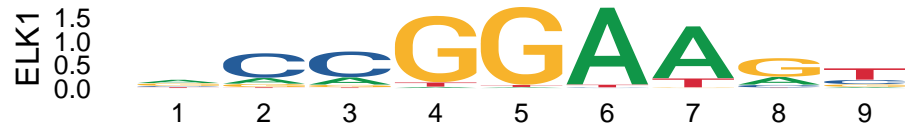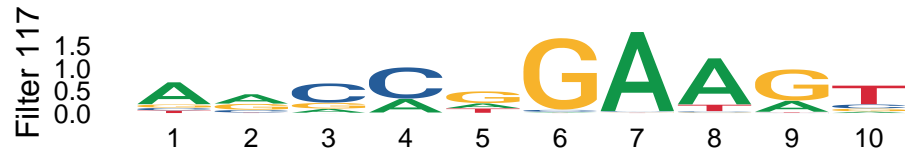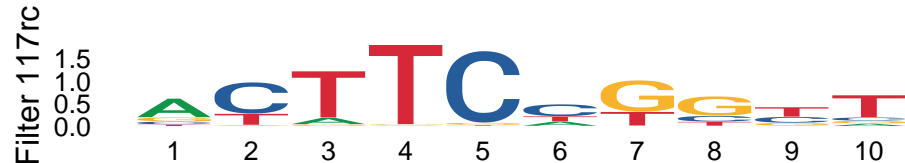

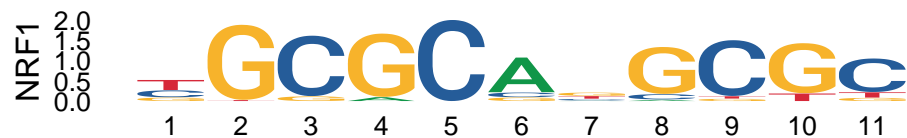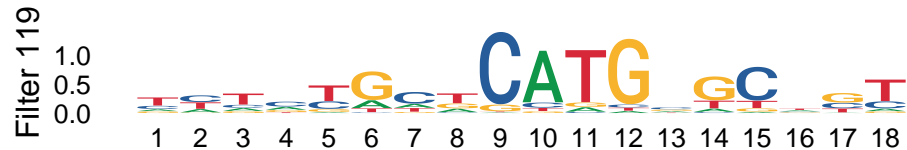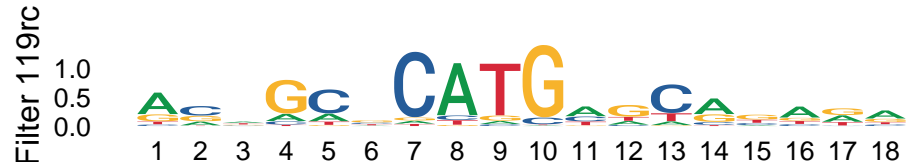

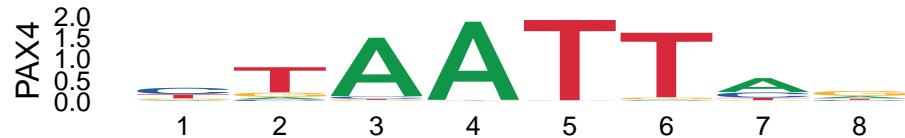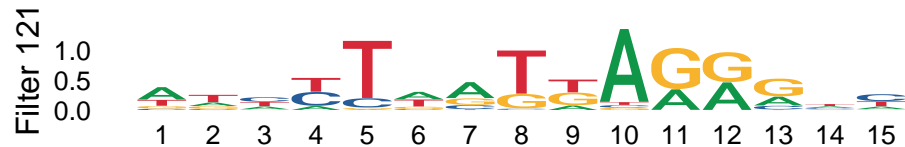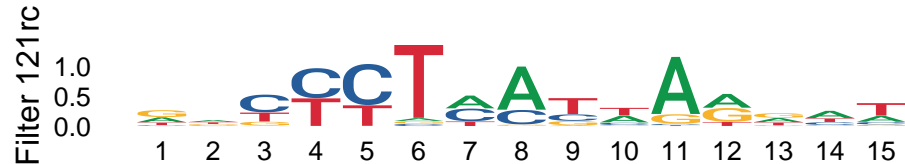

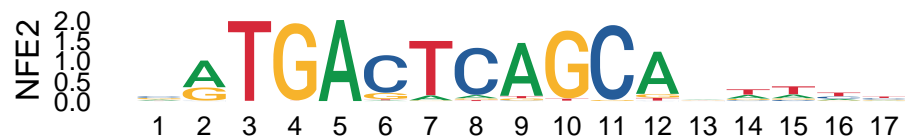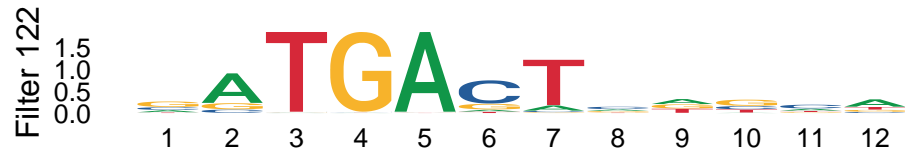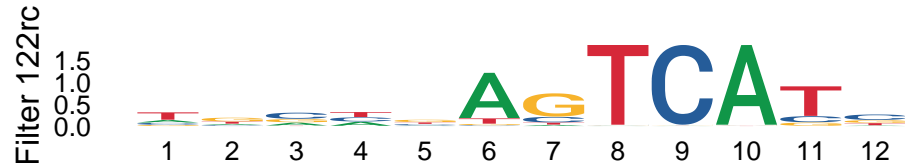

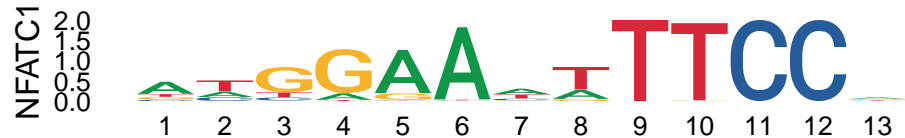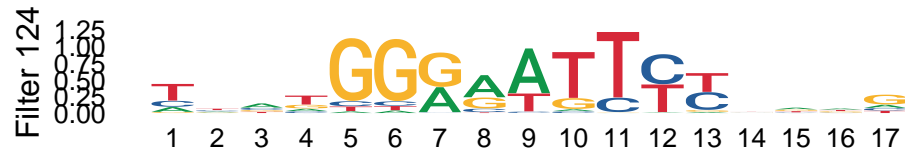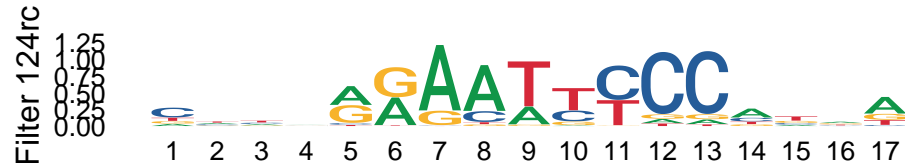

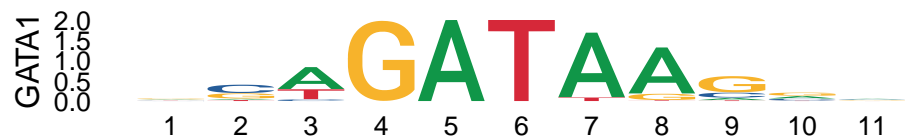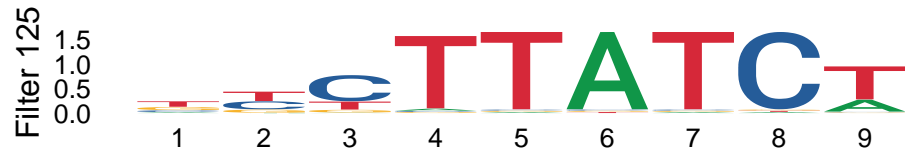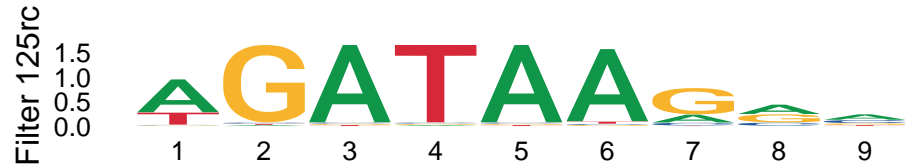

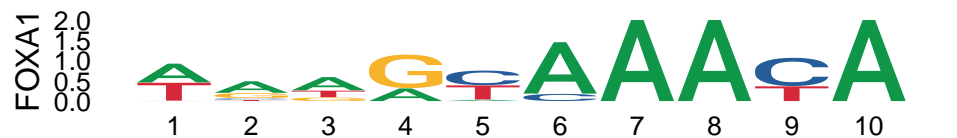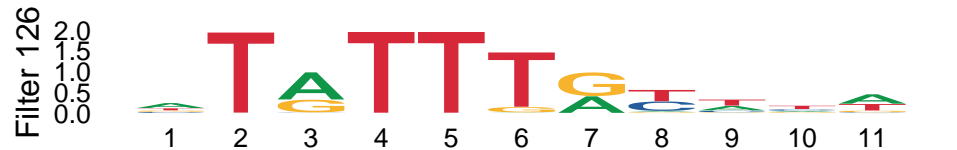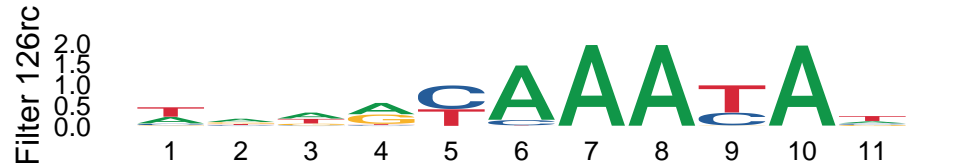

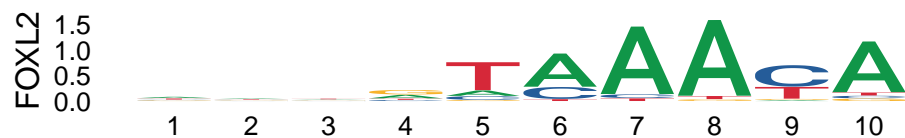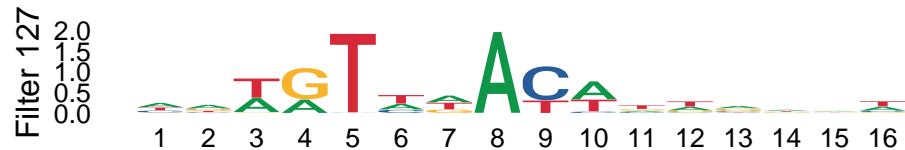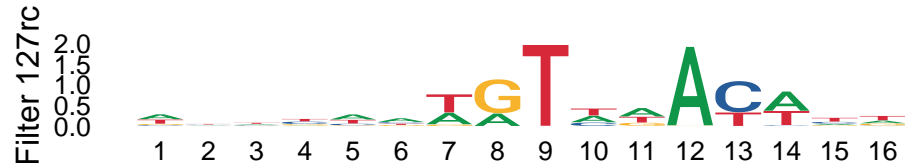

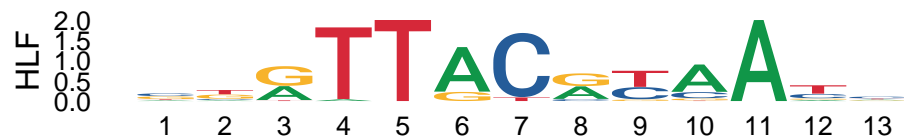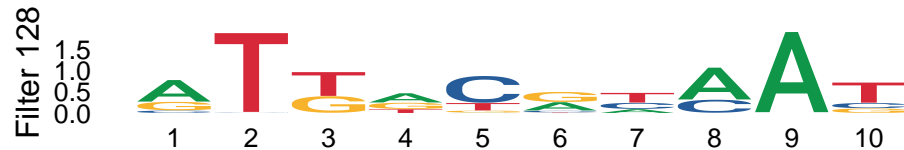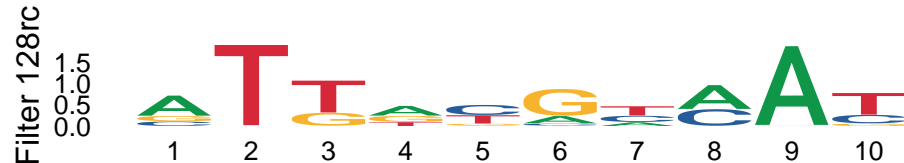

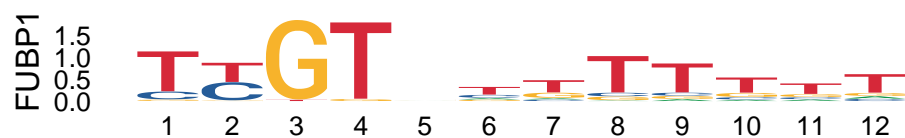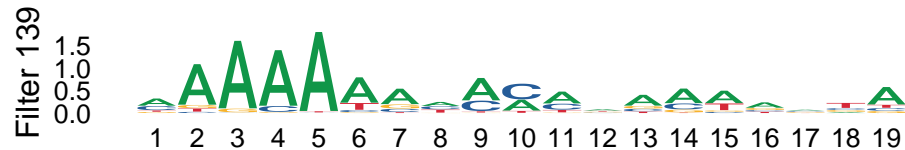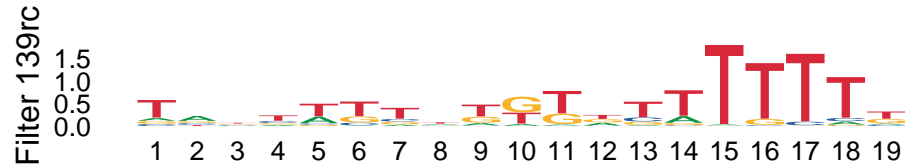

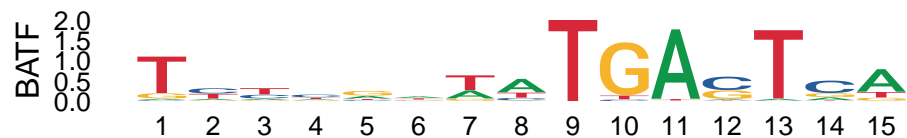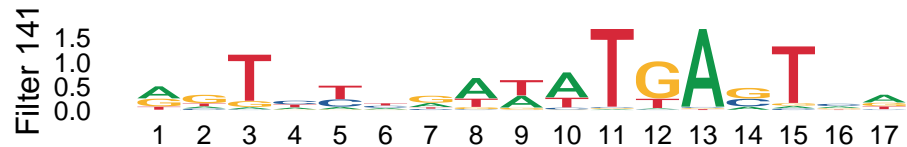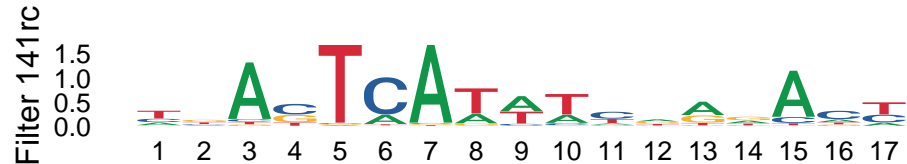

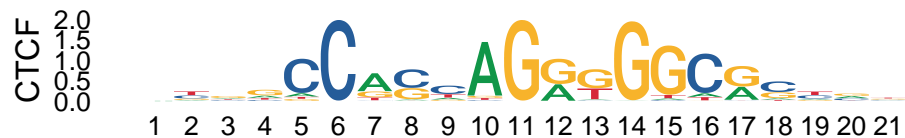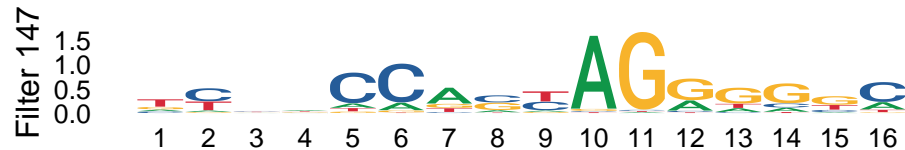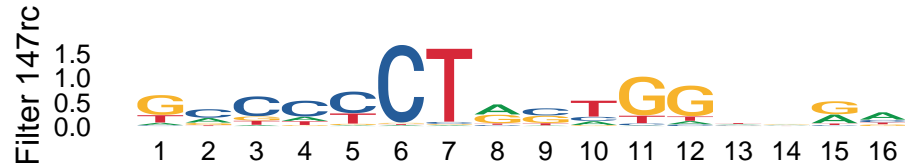

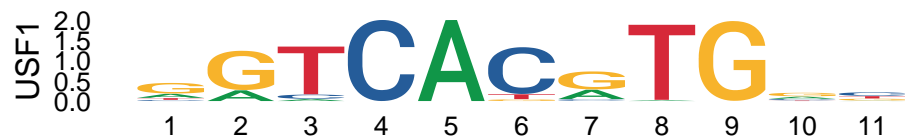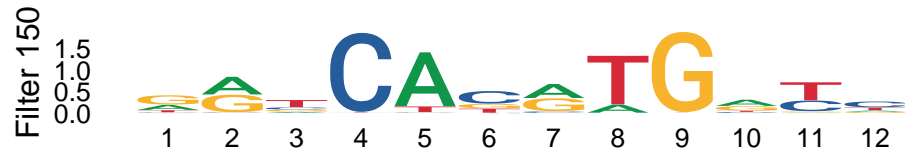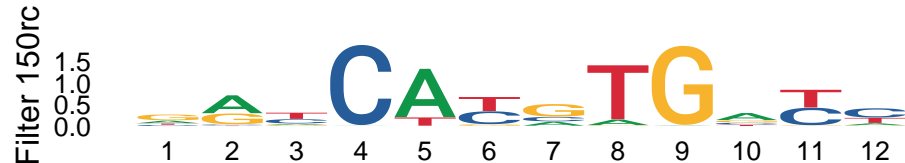

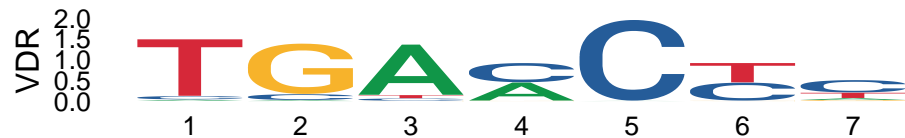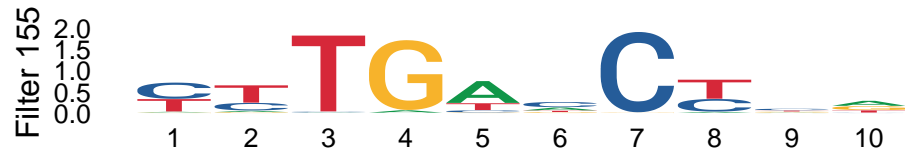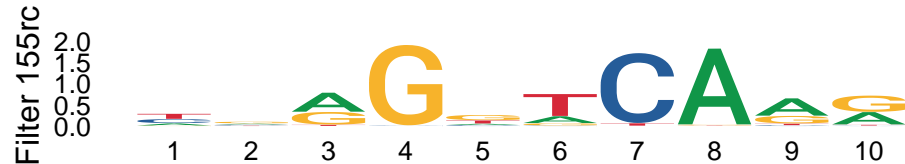



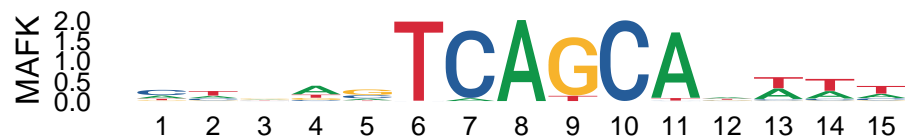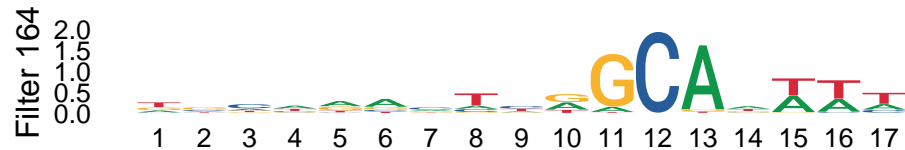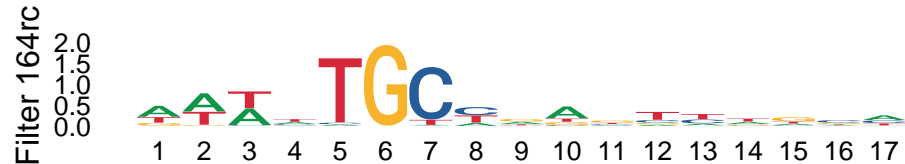

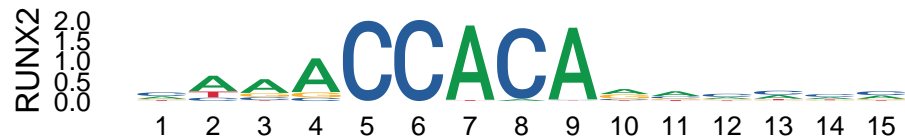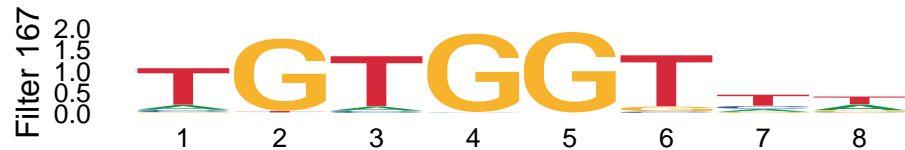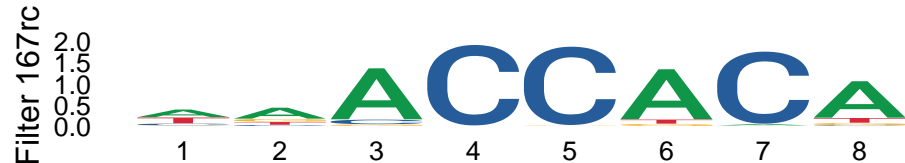

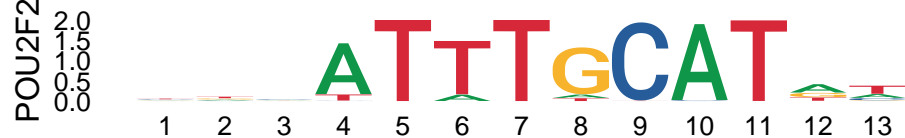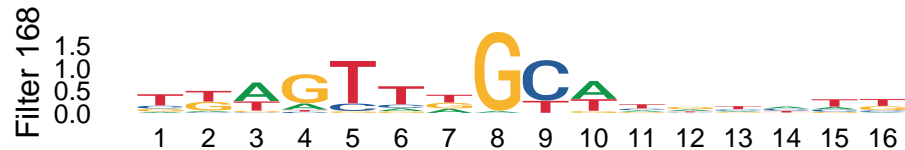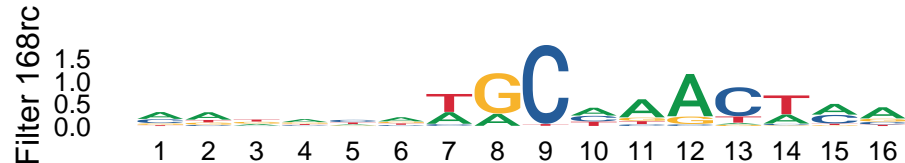

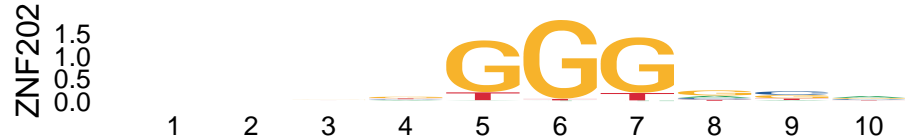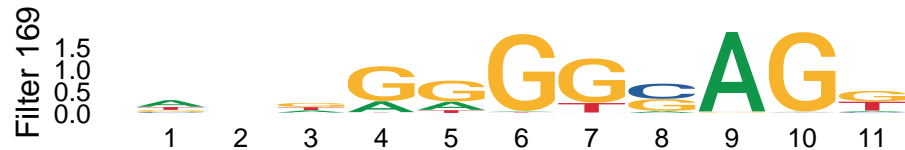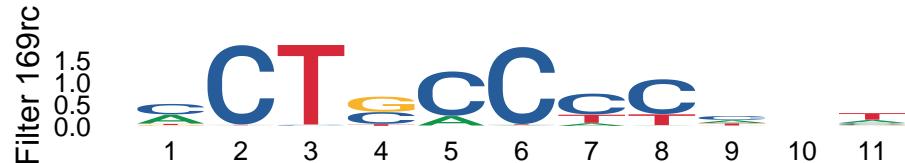

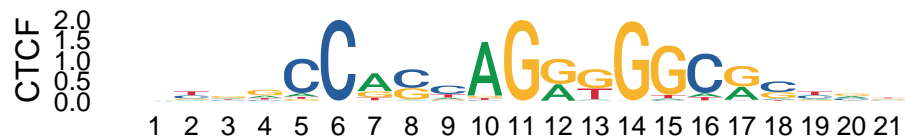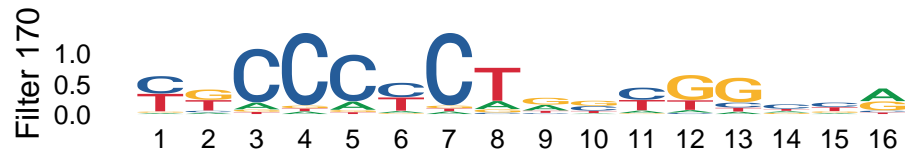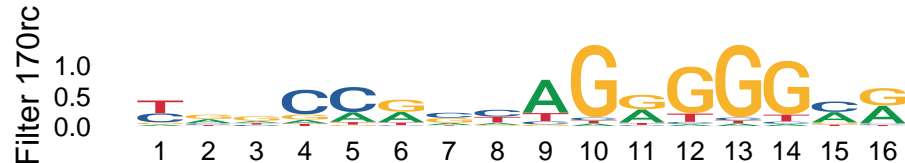

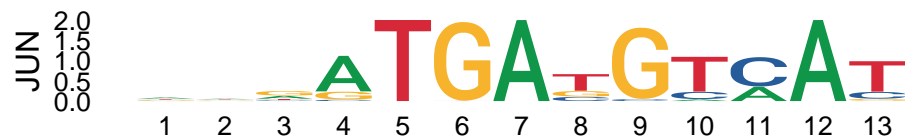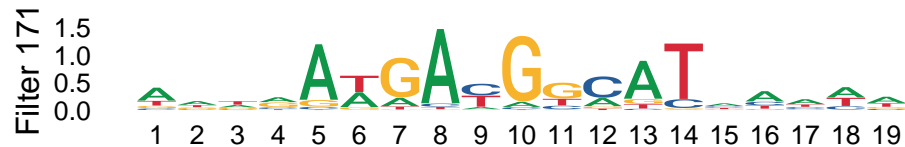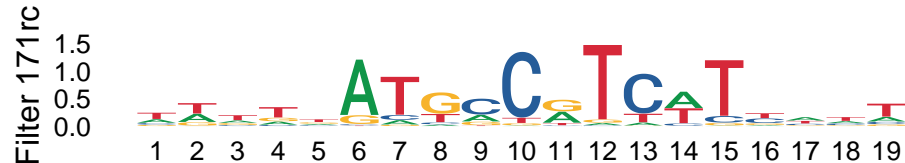

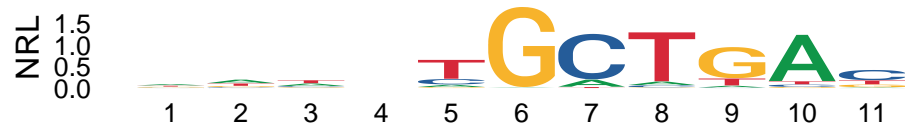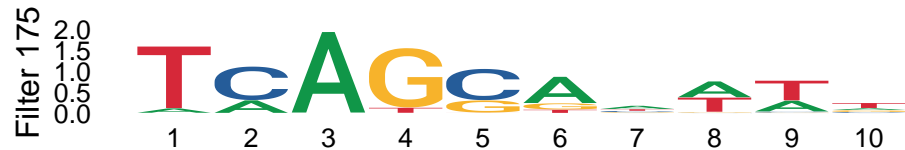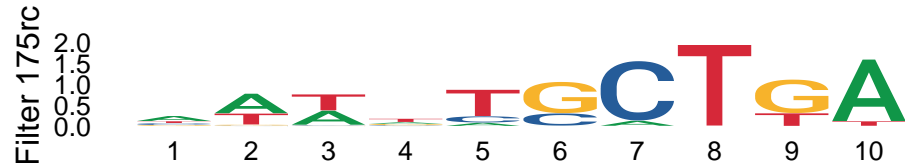

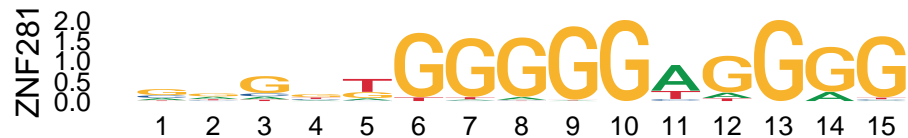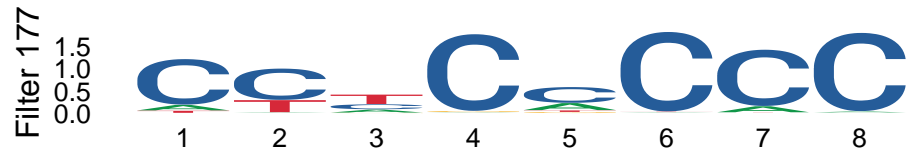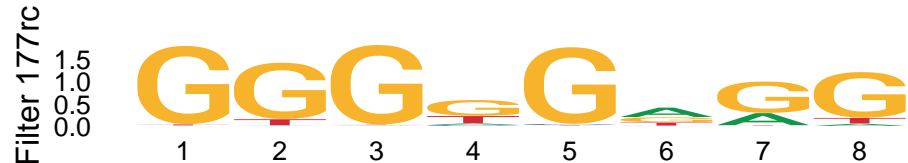

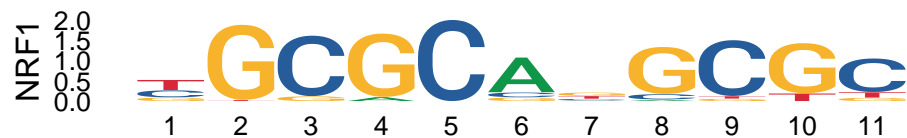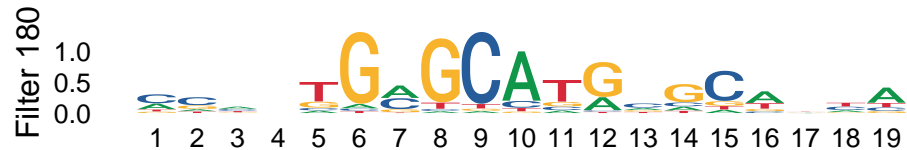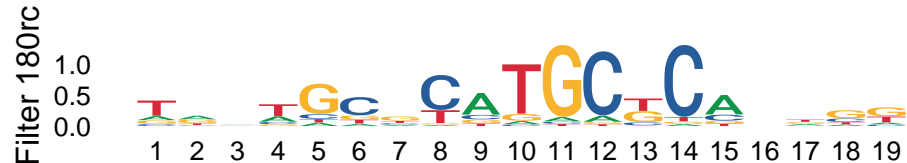

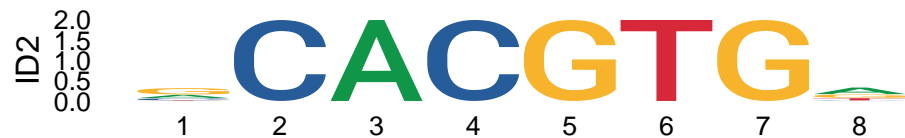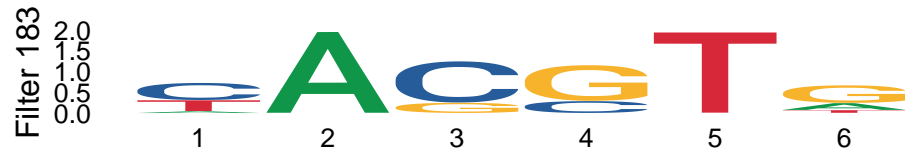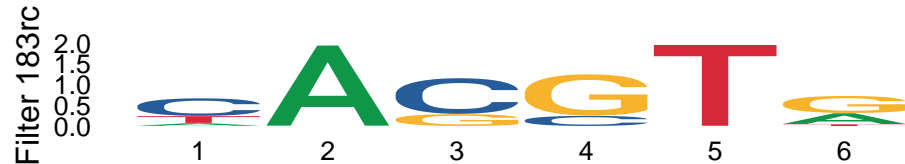

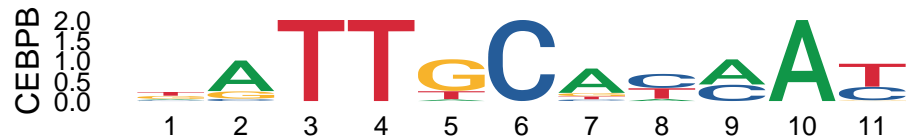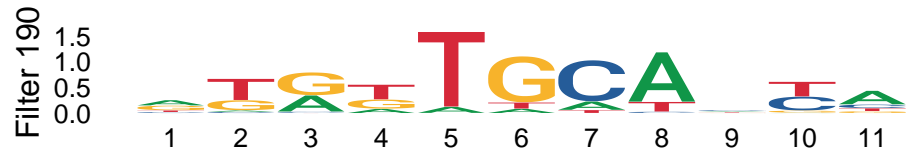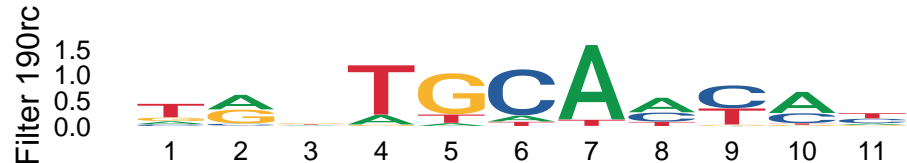

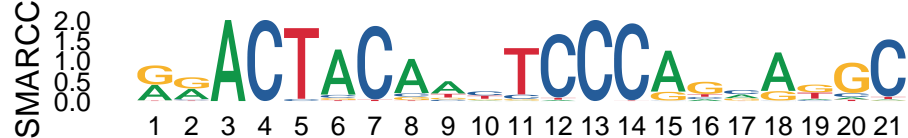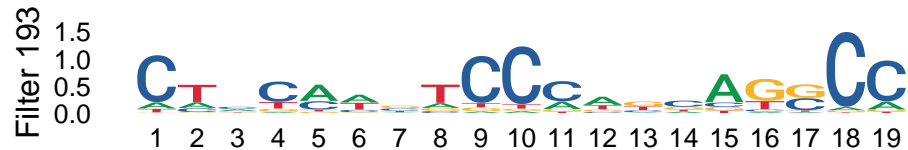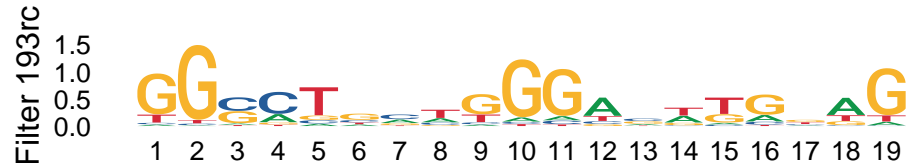

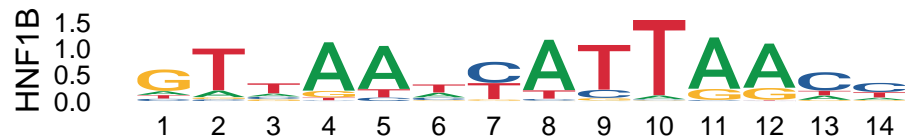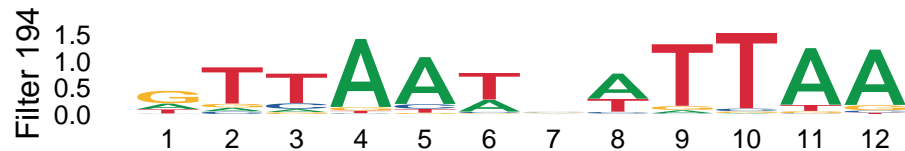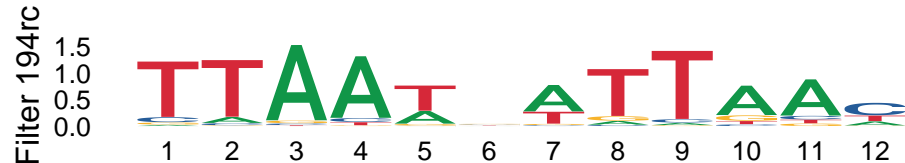

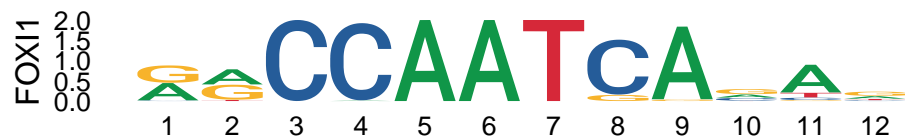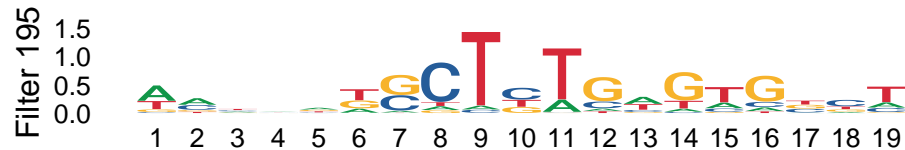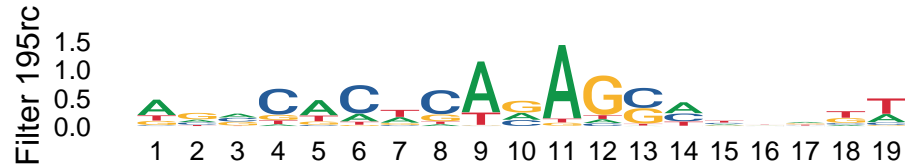

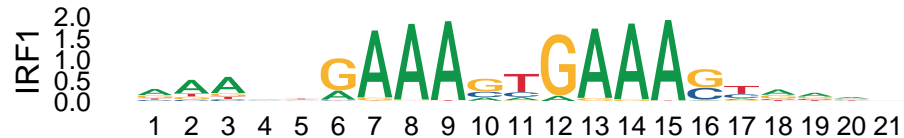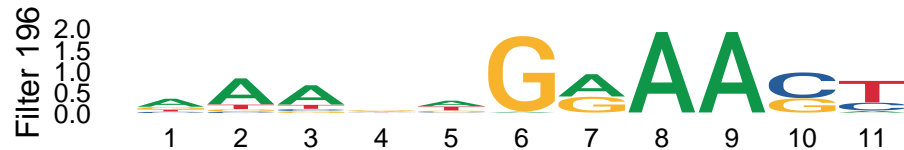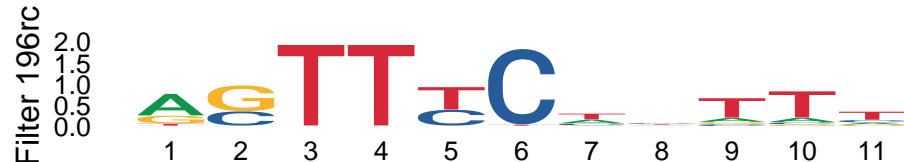

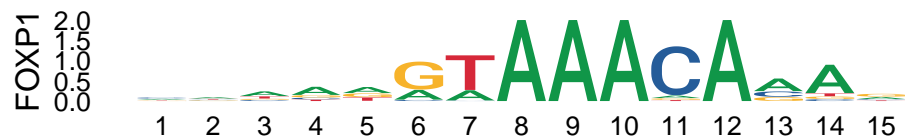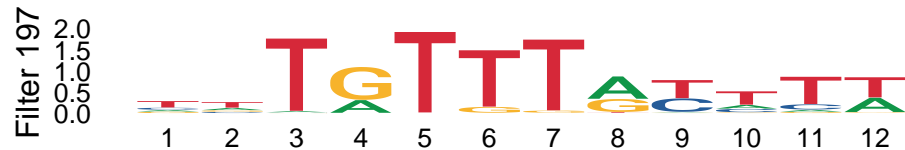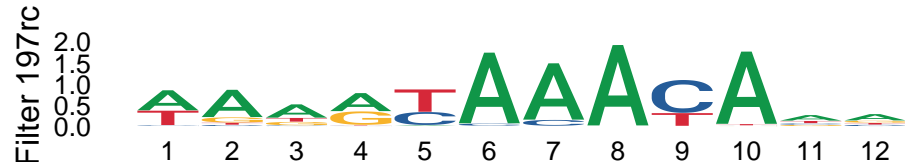

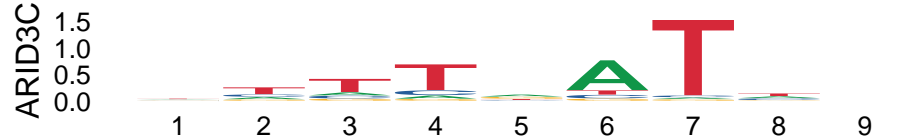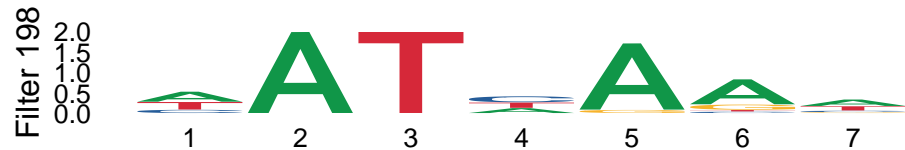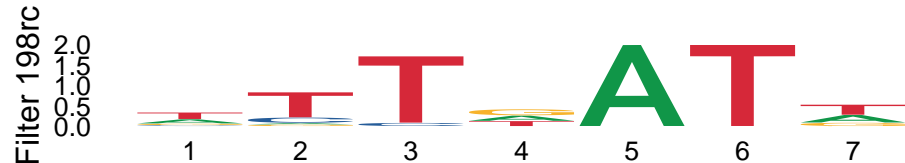

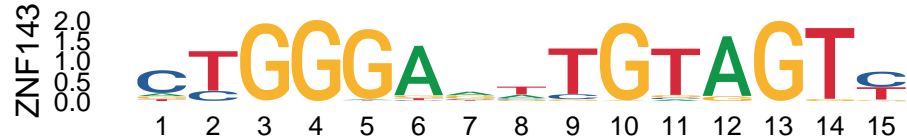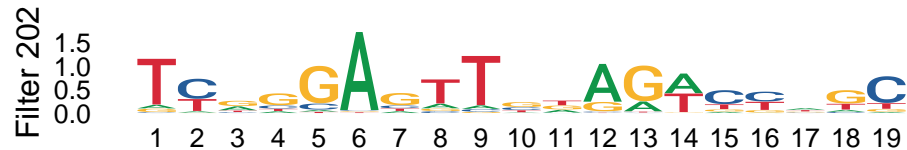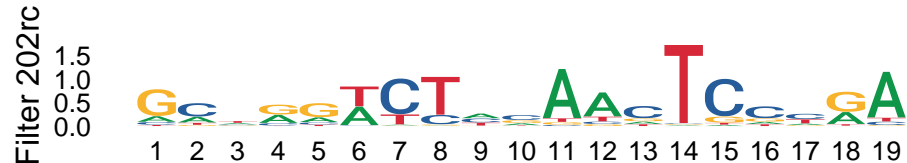

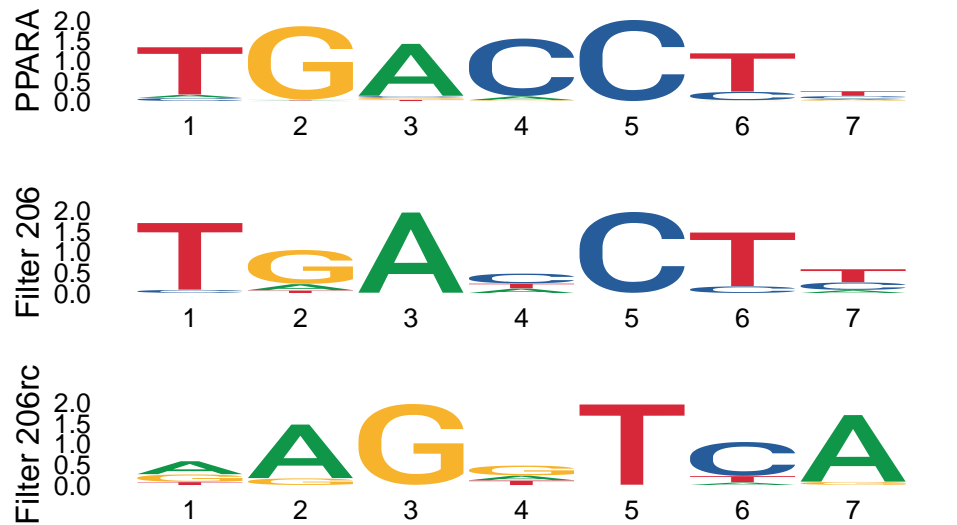

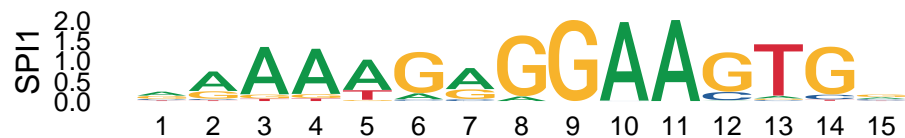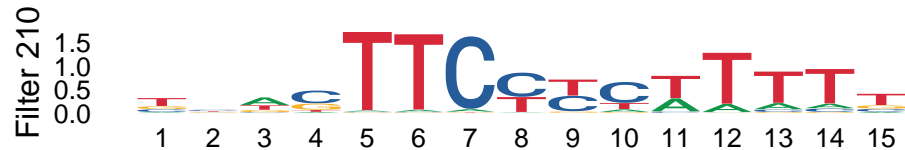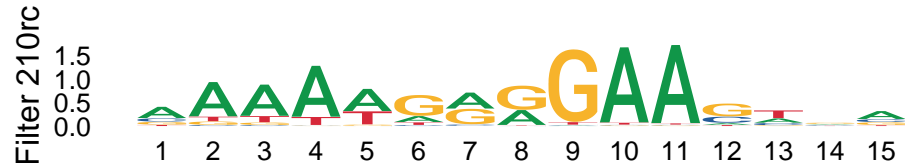

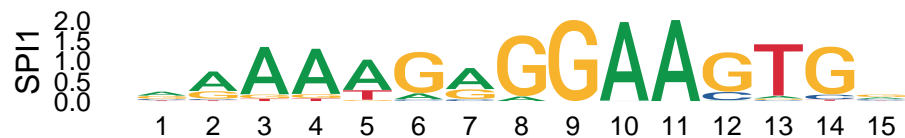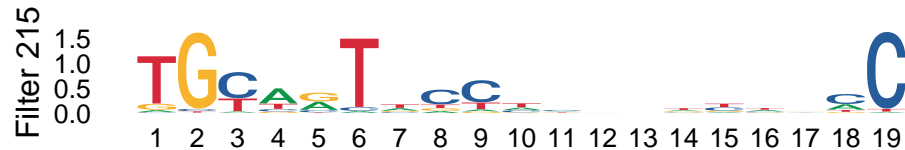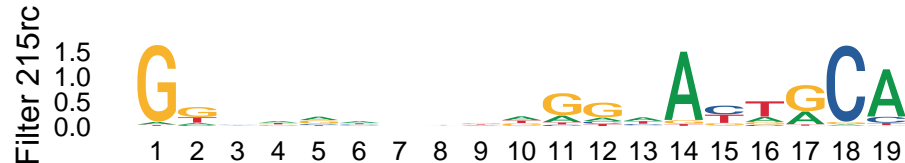

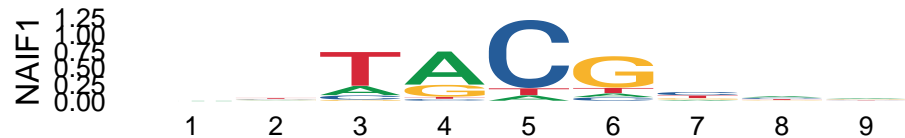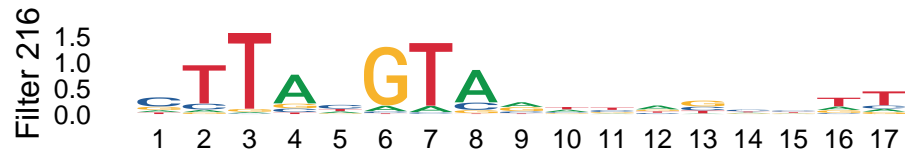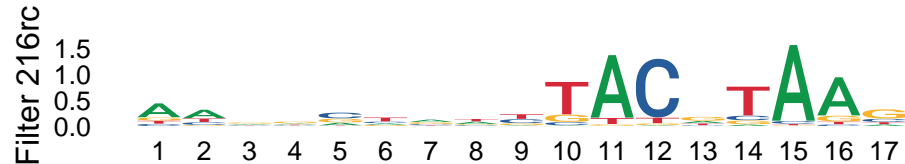

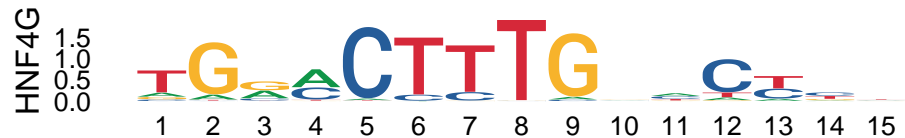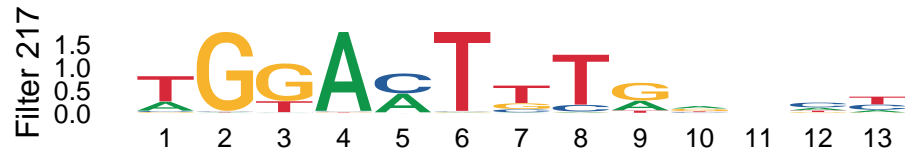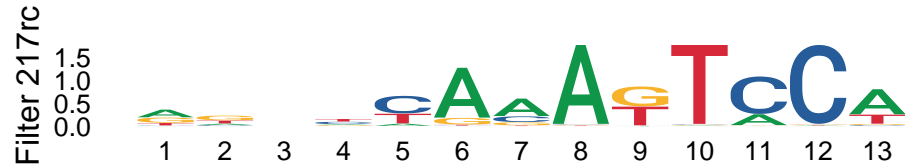

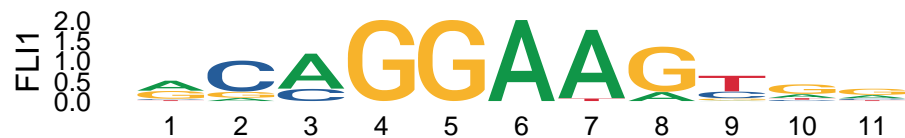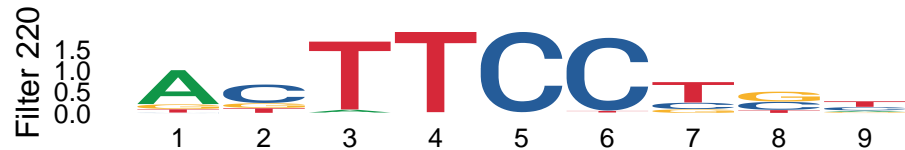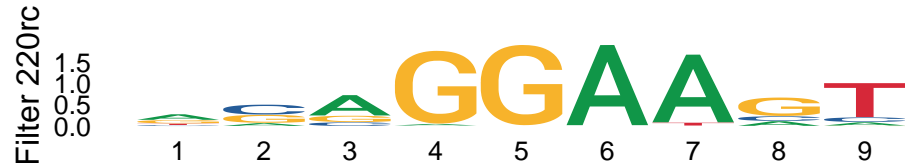

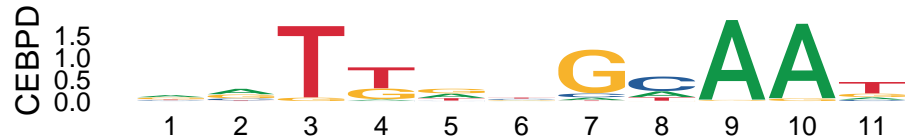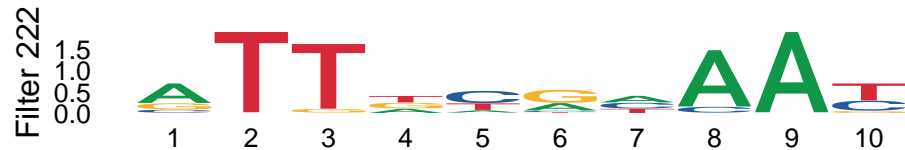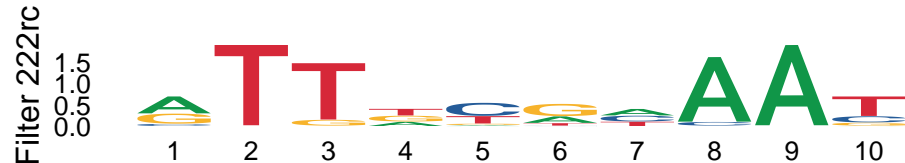





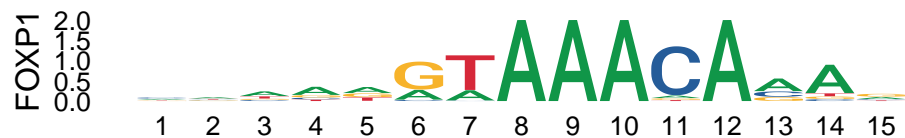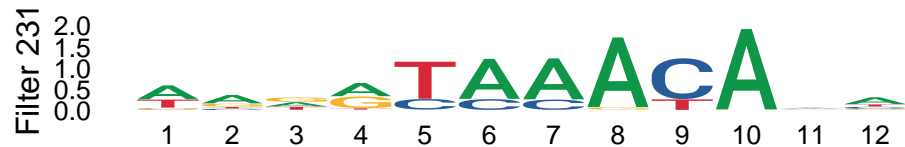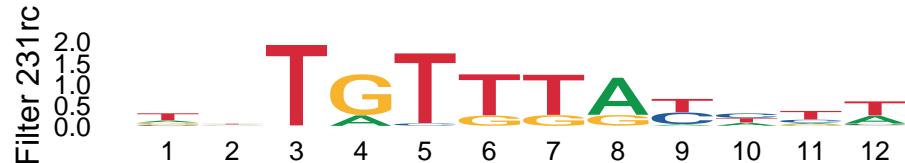

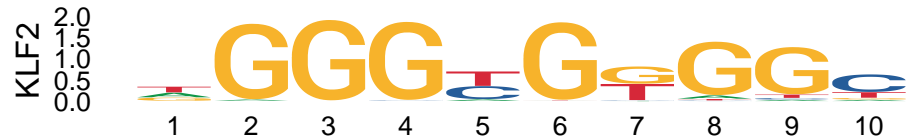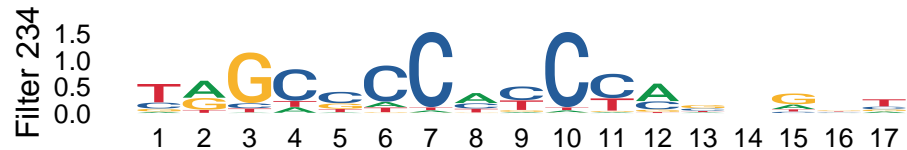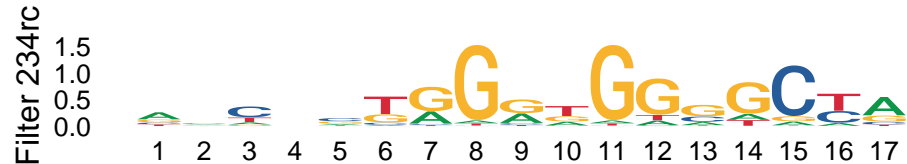

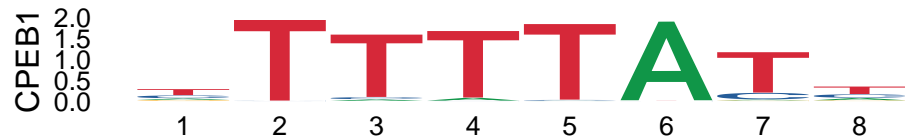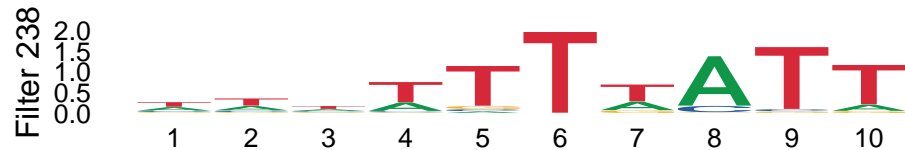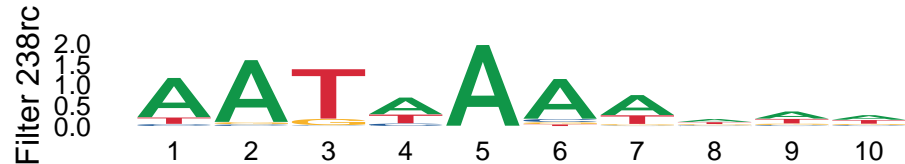

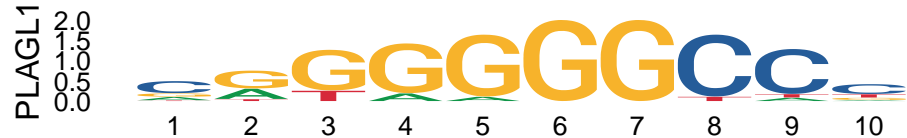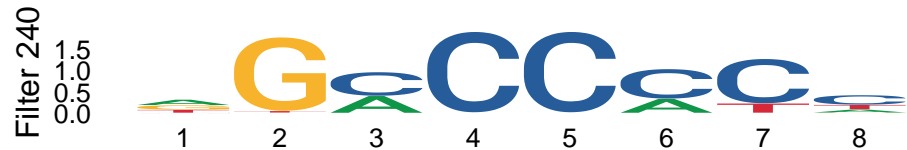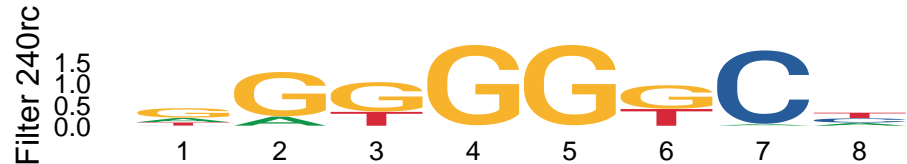

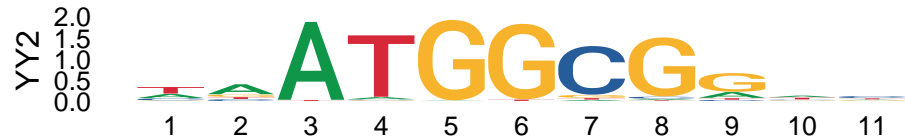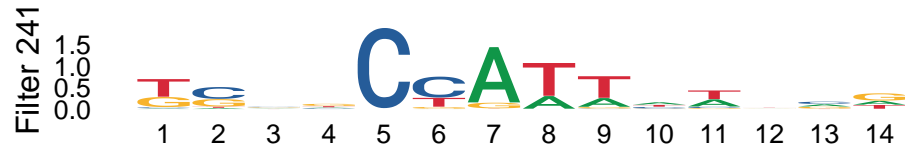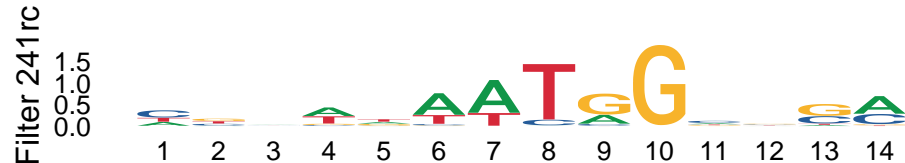

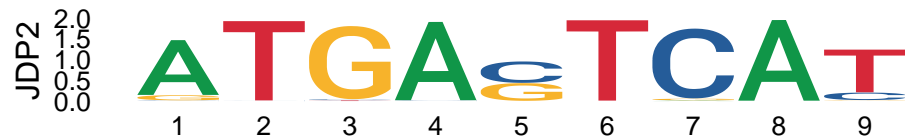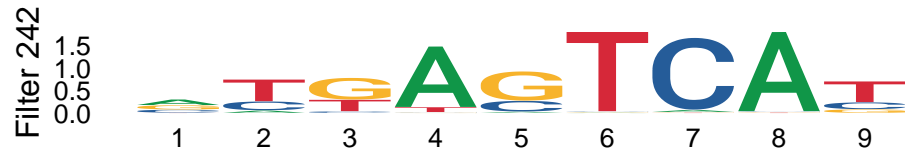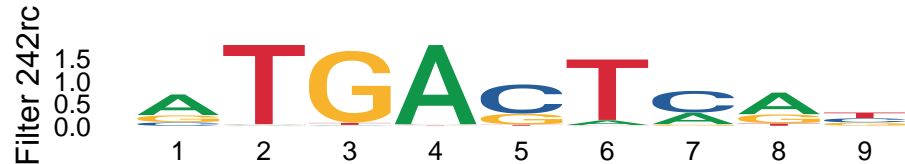

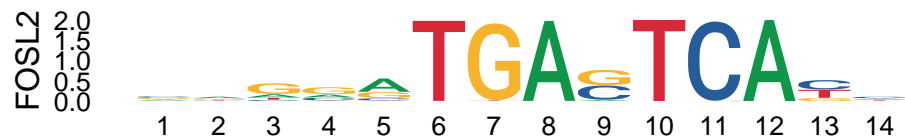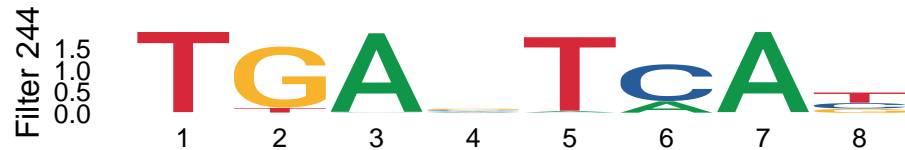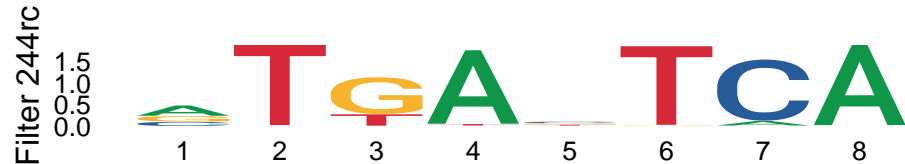

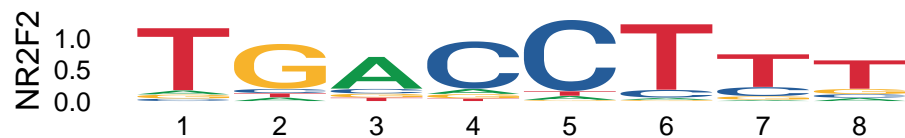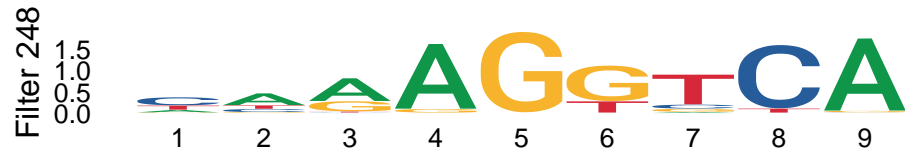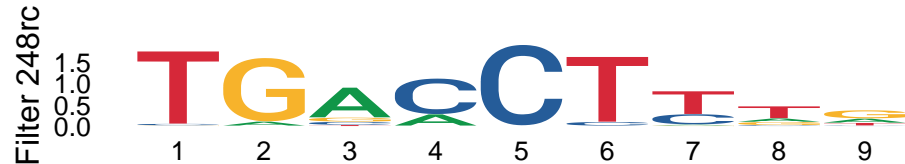

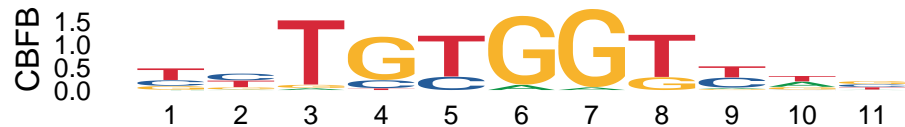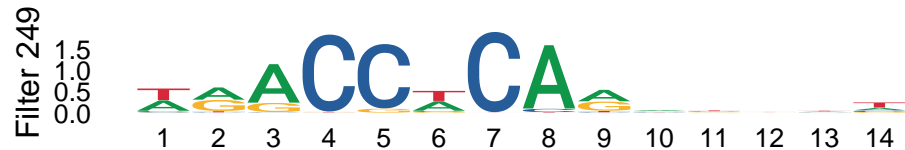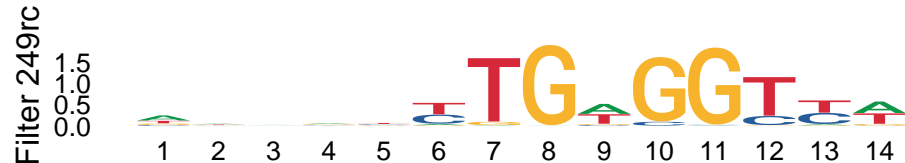

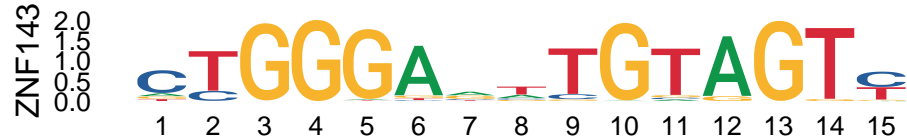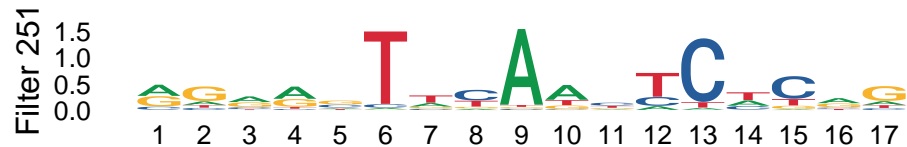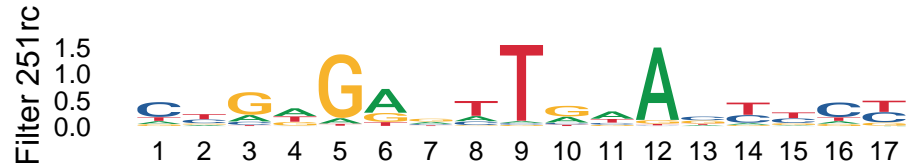

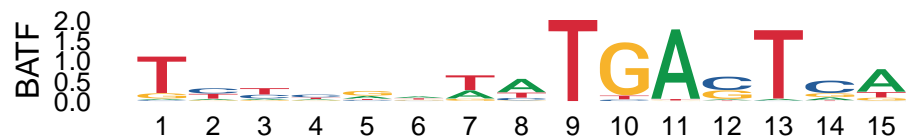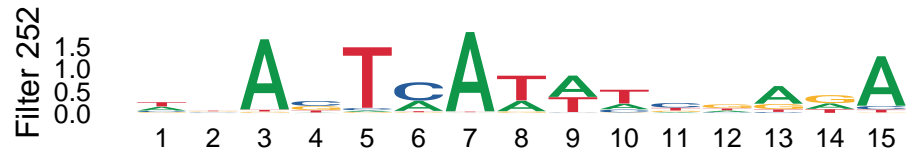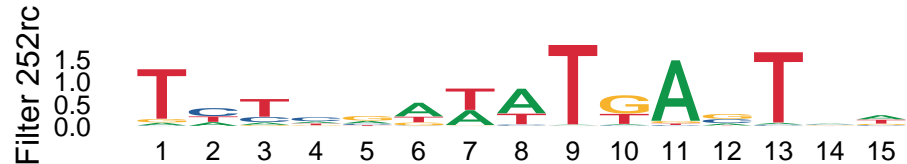

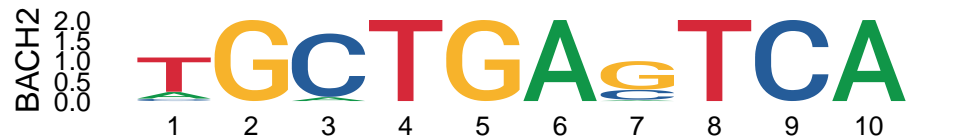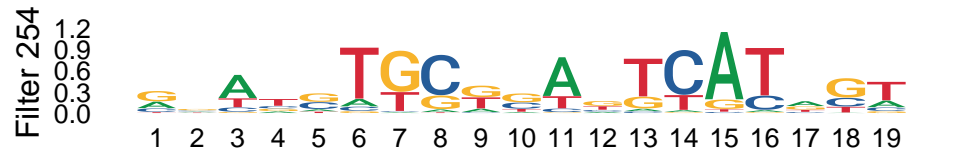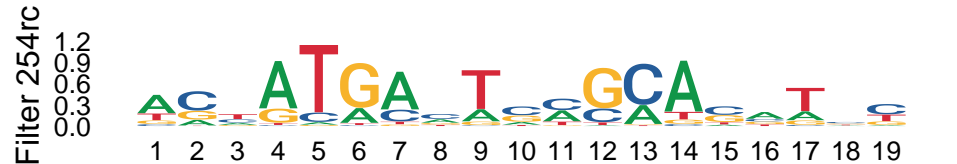

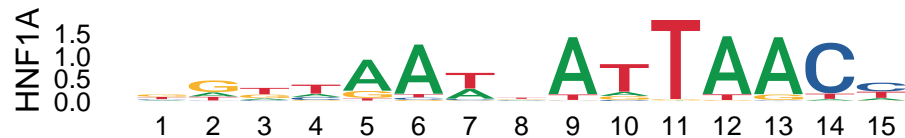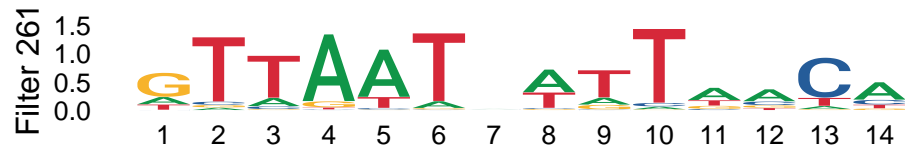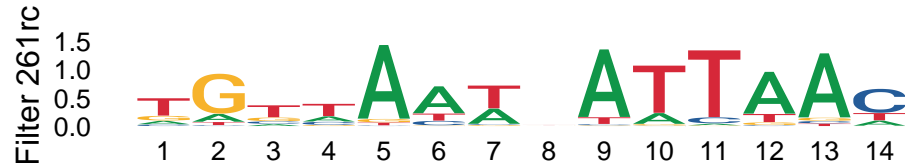

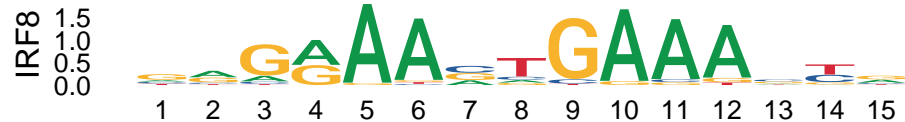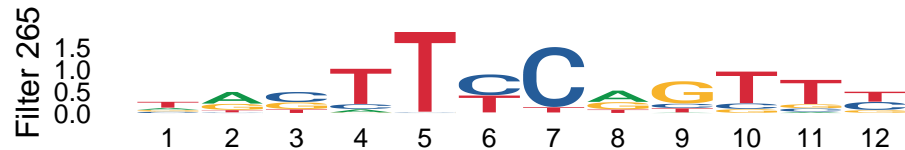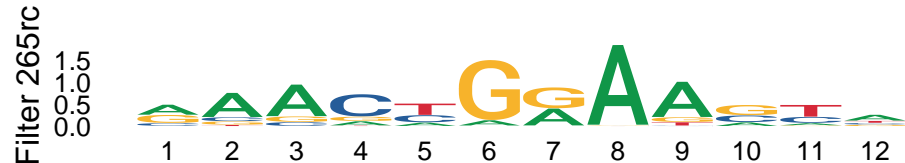

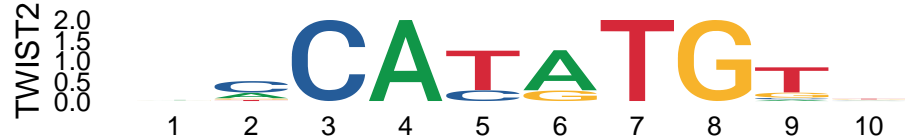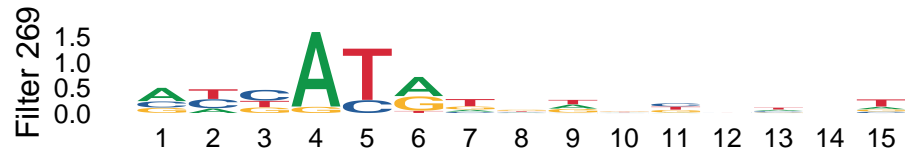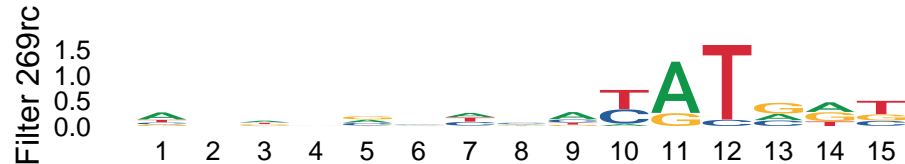

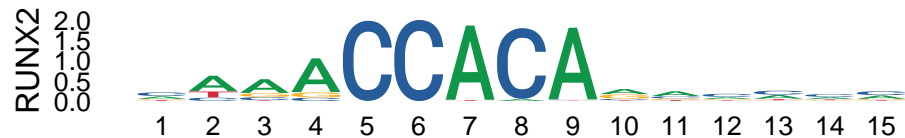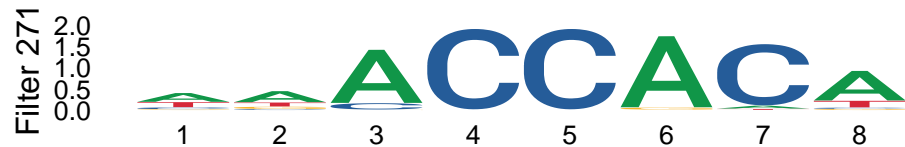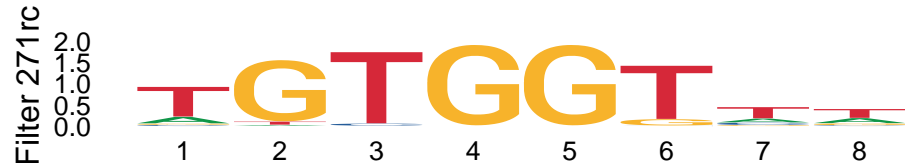

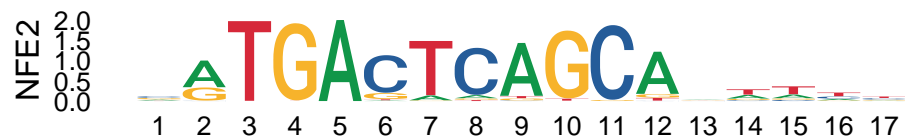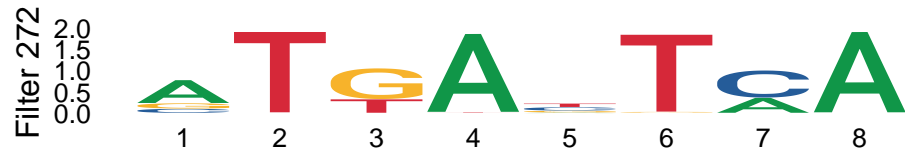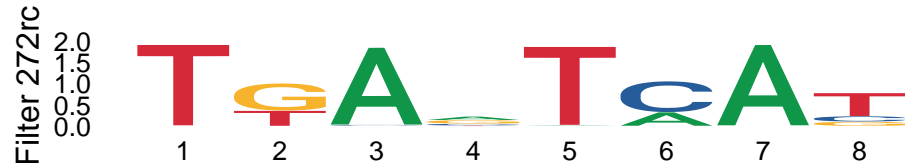

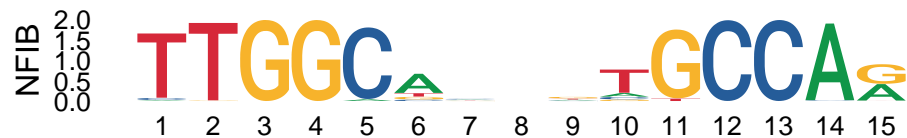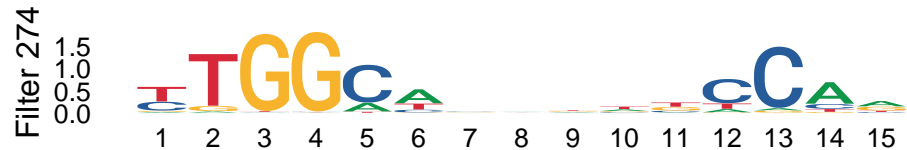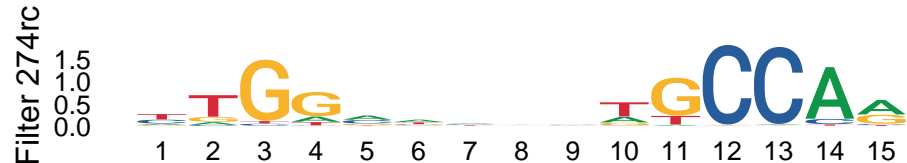

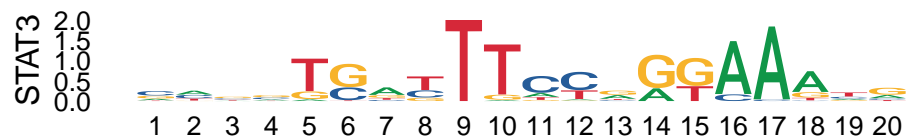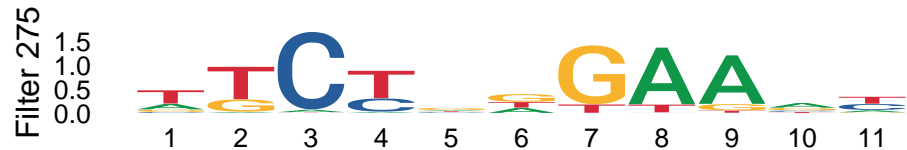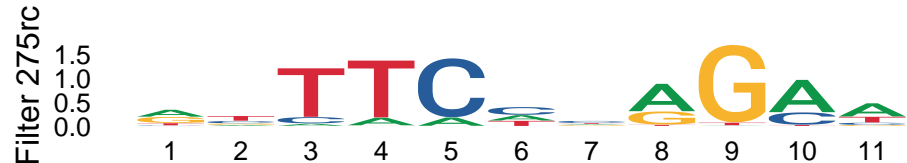

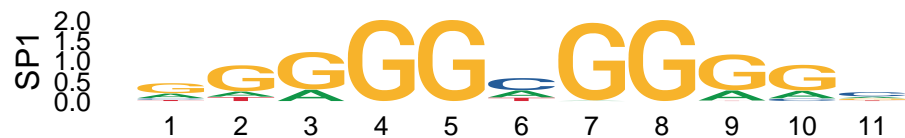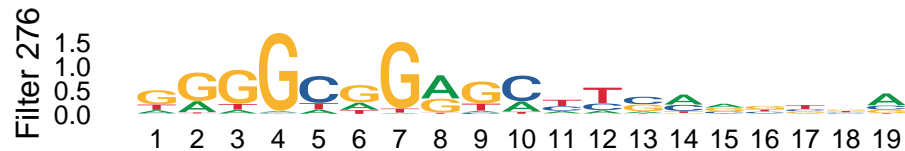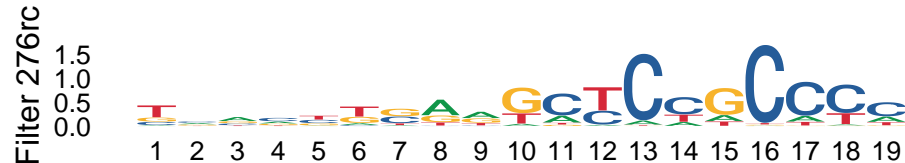

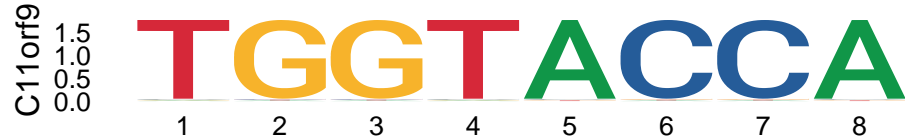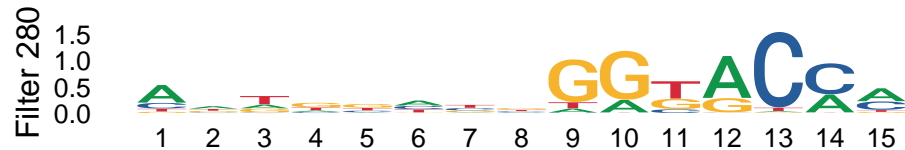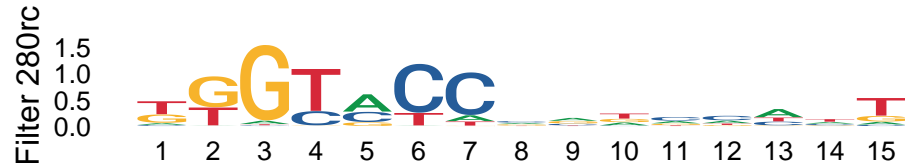

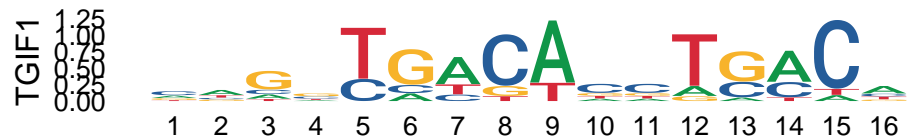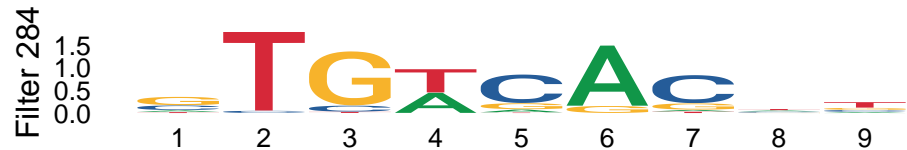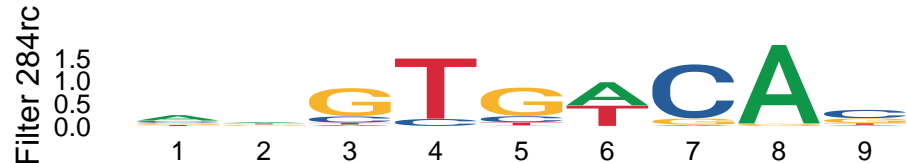

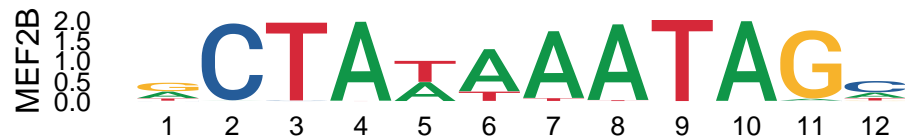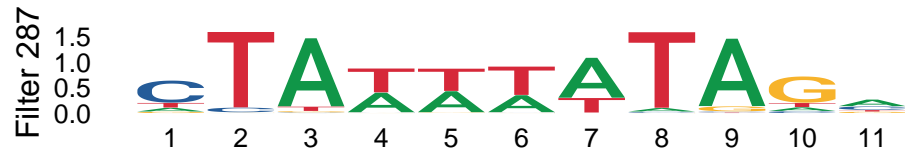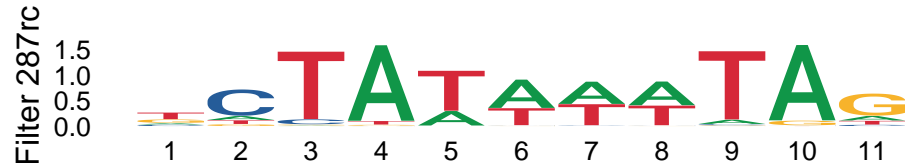

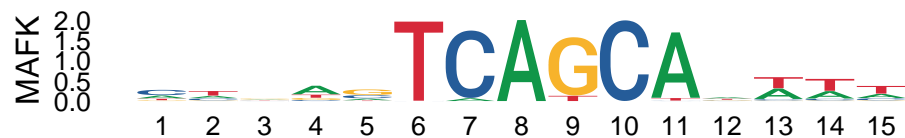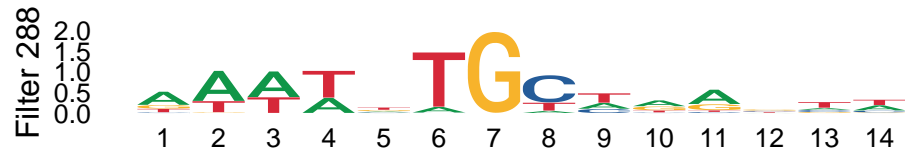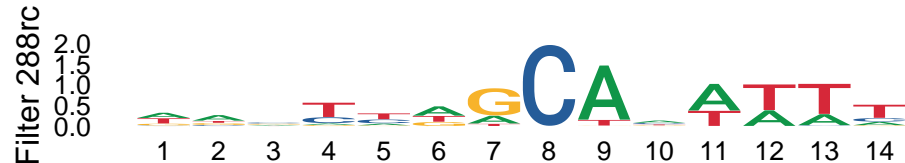

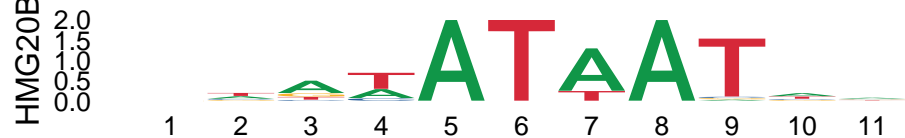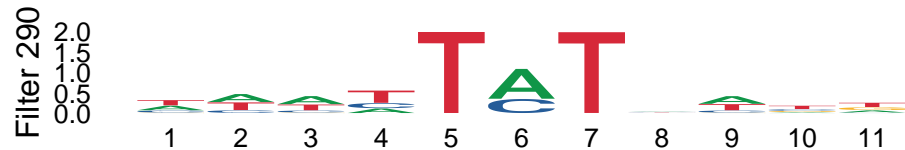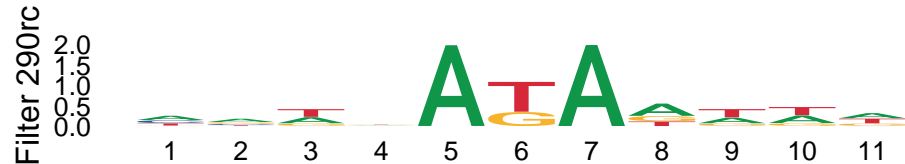

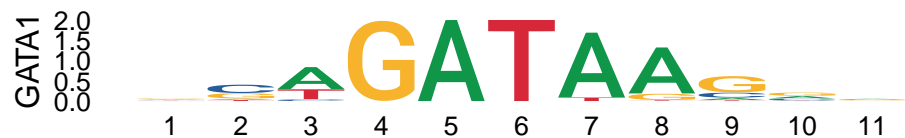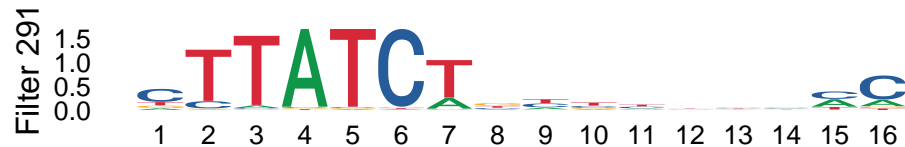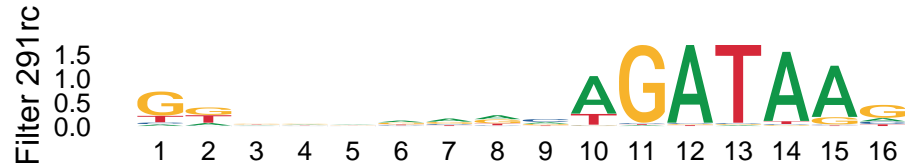

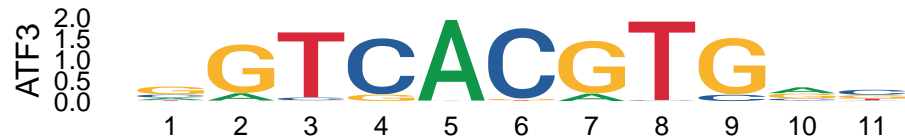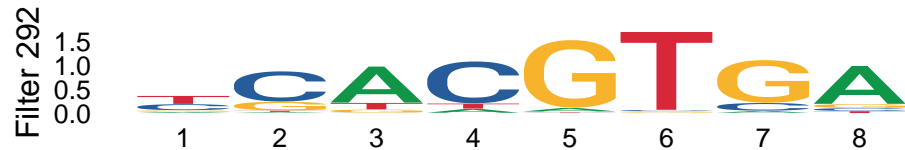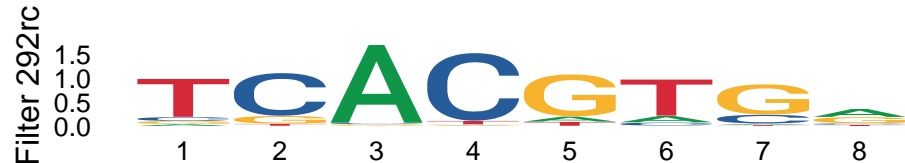

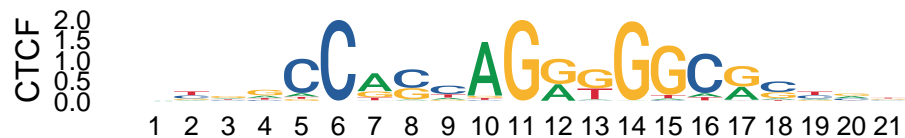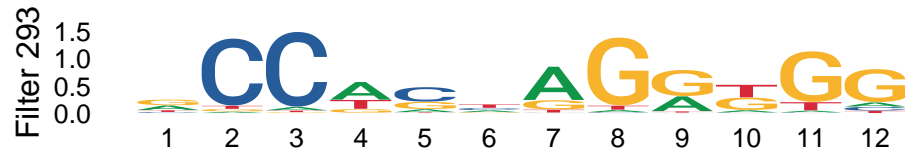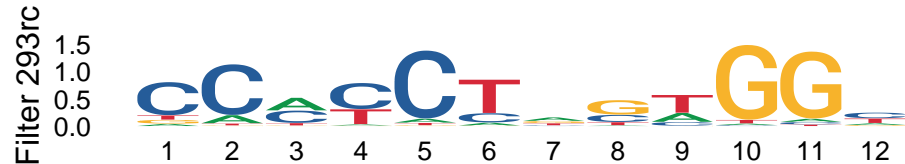

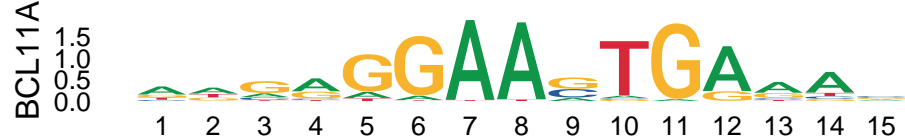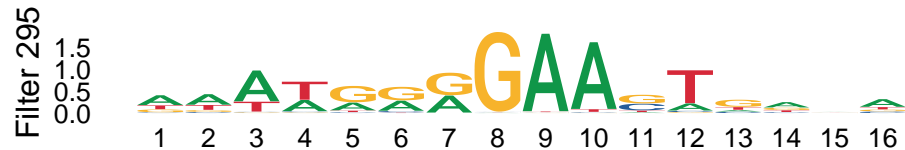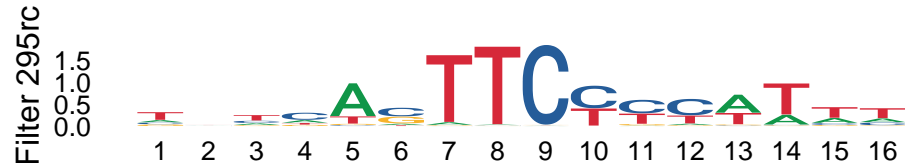

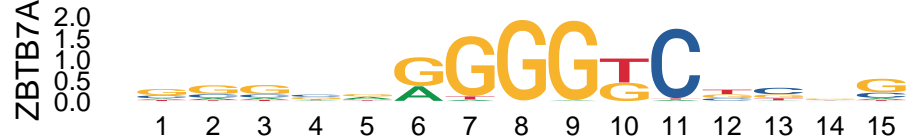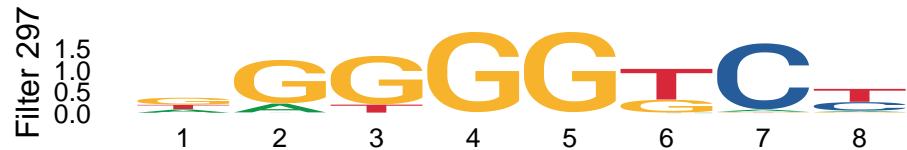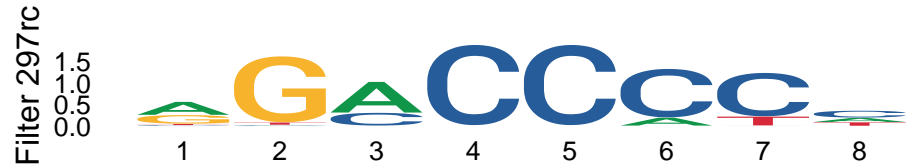

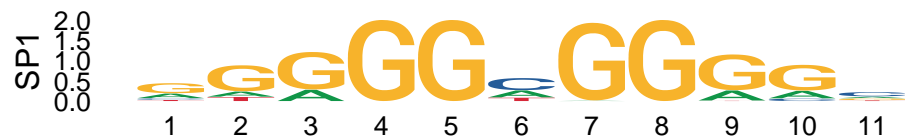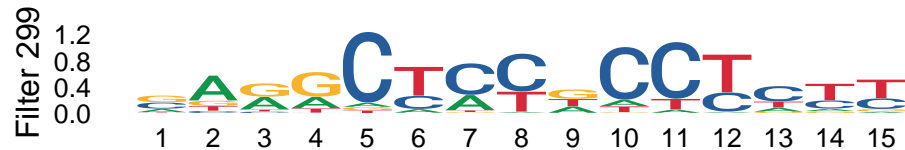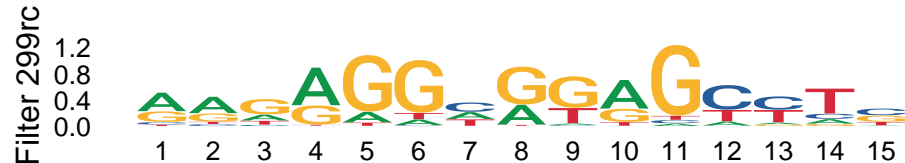

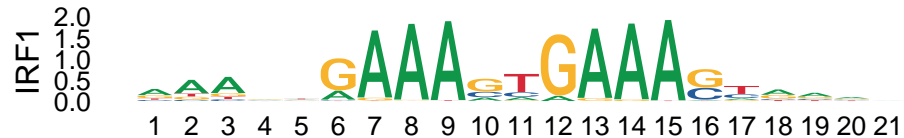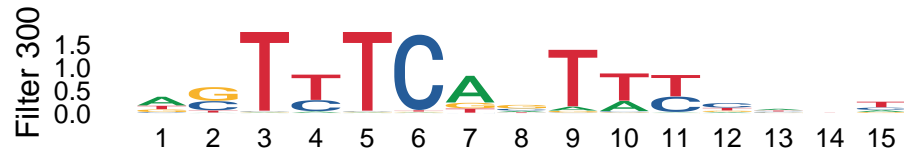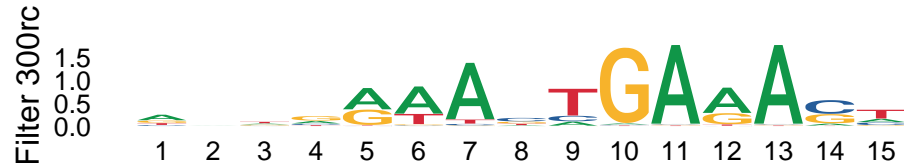

Supplement: gkaa1137_Supplemental_Files [file gkaa1137_supplemental_files.zip › Supplementary Figure S2.pdf]

A

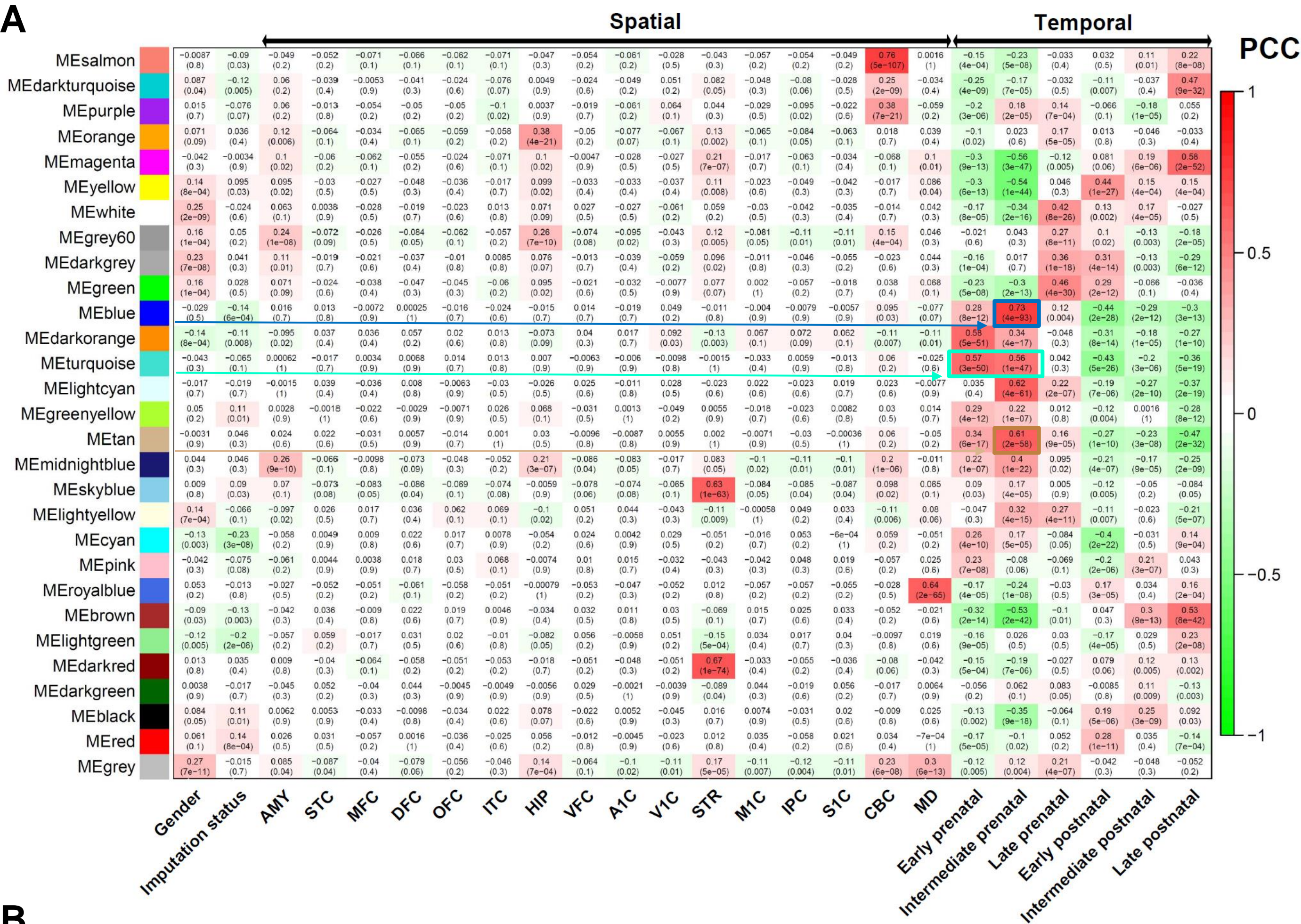

B

Tissue specific enrichment analysis for genes in each module

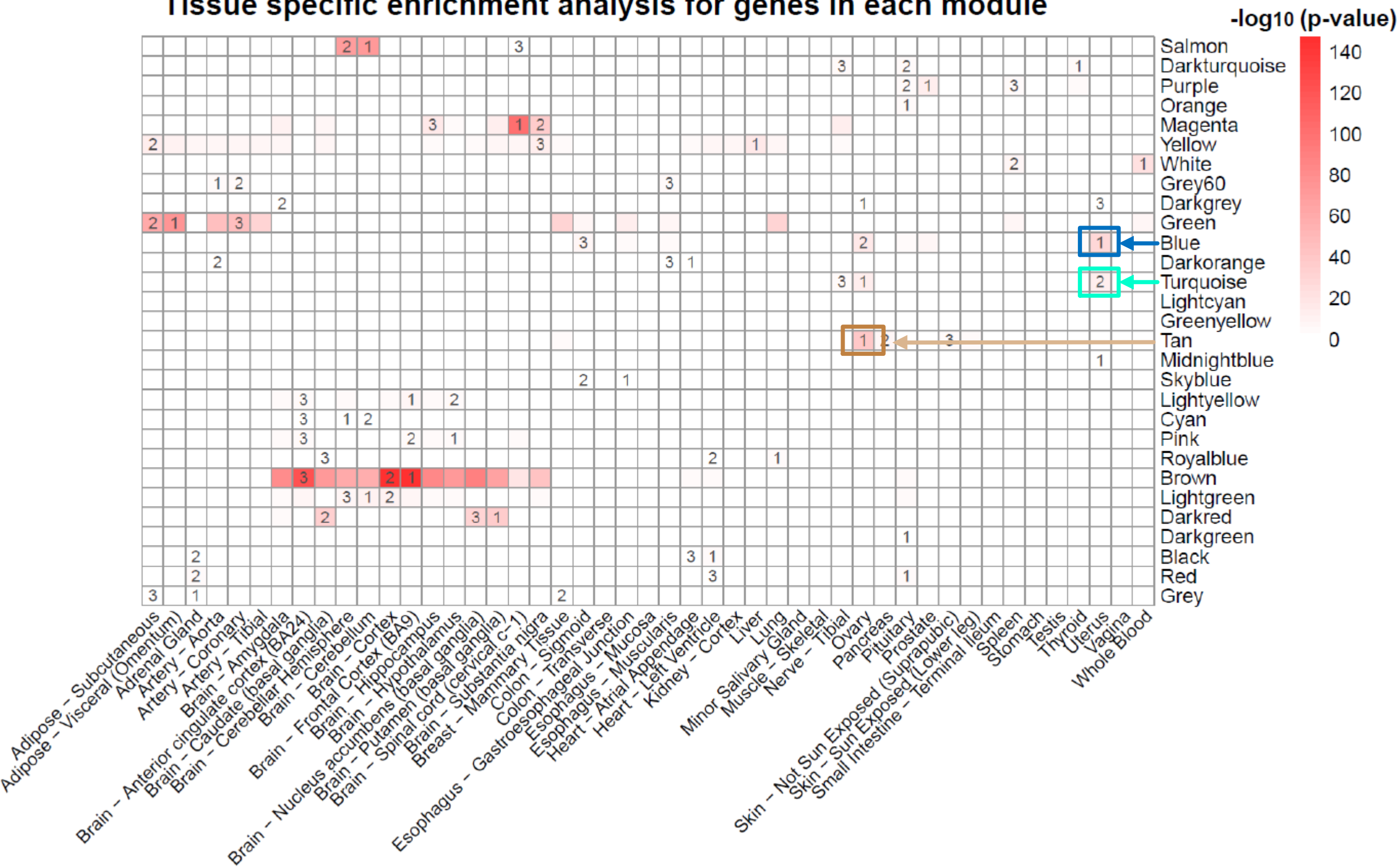

Supplement: gkaa1137_Supplemental_Files [file gkaa1137_supplemental_files.zip › Supplementary Figure S5.pdf]

Association between non-regulatory TAG and tissue-specific expressed genes

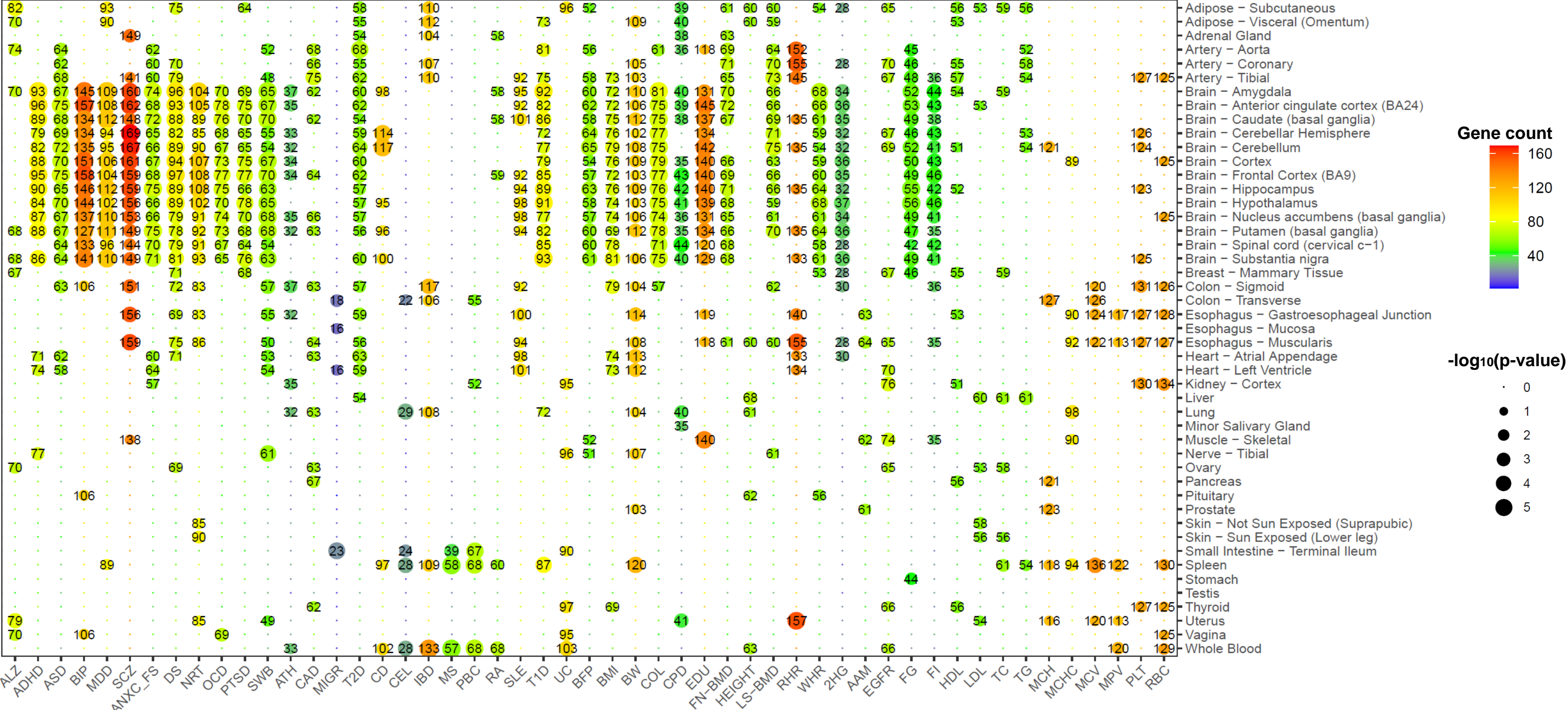

Supplement: gkaa1137_Supplemental_Files [file gkaa1137_supplemental_files.zip › Supplementary Figure S6.pdf]

A

rs11685464 G -&gt; C

FOX motif

TGA\_TCA\_T

Accessibility difference

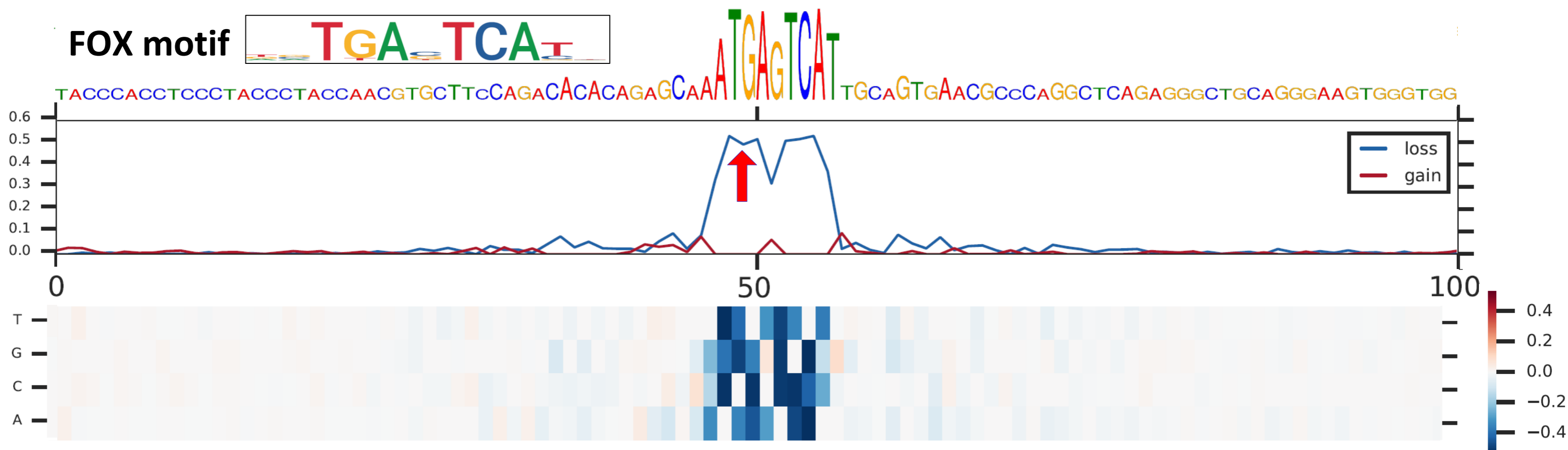

B

Accessibility difference

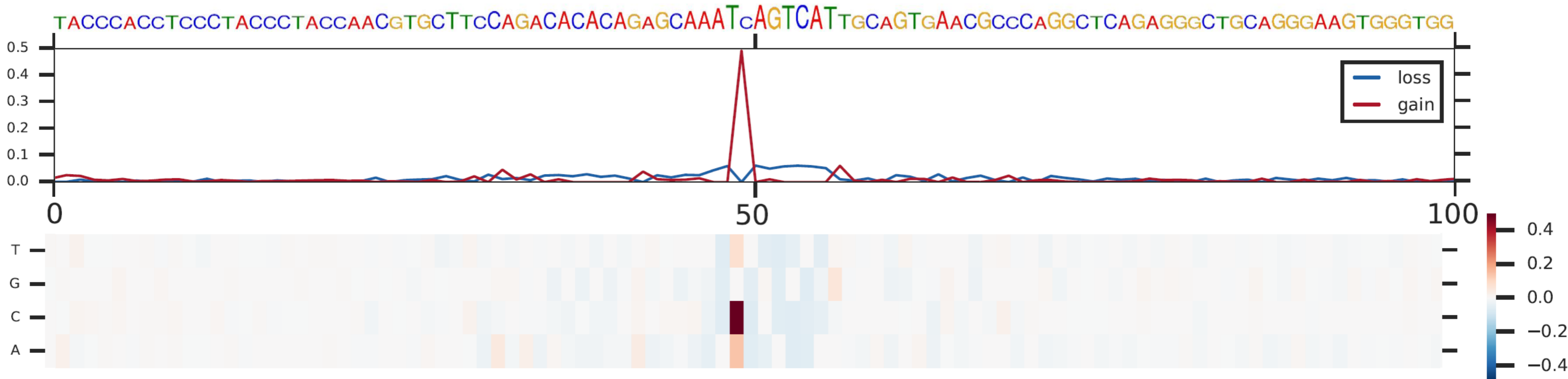

Supplement: gkaa1137_Supplemental_Files [file gkaa1137_supplemental_files.zip › Supplementary Figure S7.pdf]
